# Supplementary figures and images for: ATR promotes mTORC1 activity via de novo cholesterol synthesis (part 1 of 2)
Source: EMBO Rep. 2025 Jun 13;26(14):3574–93. doi: 10.1038/s44319-025-00451-3 (PMC12287318; doi:10.1038/s44319-025-00451-3)

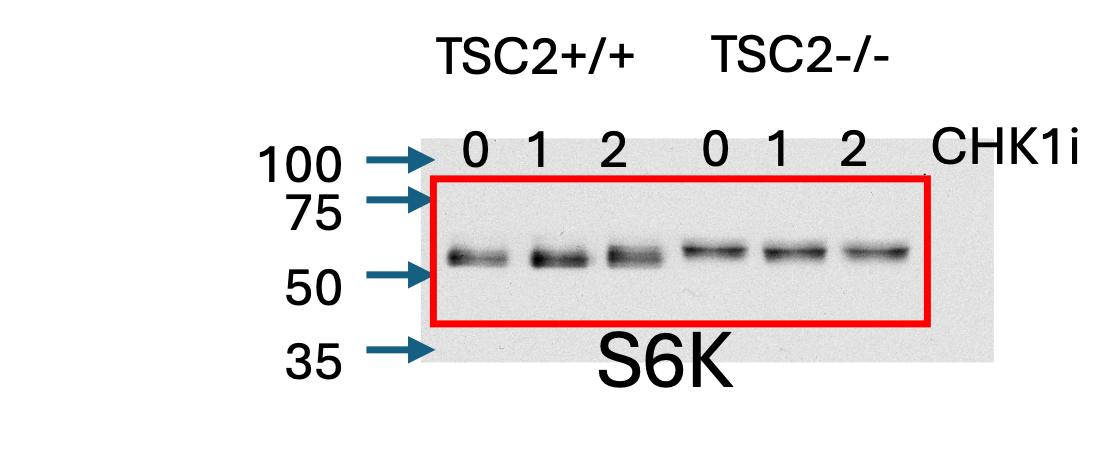

Supplement: Supplementary file 2 — Source data Fig. 1 [file 44319_2025_451_MOESM2_ESM.zip › Figure 1/Figure 1F/Western S6K.tif]

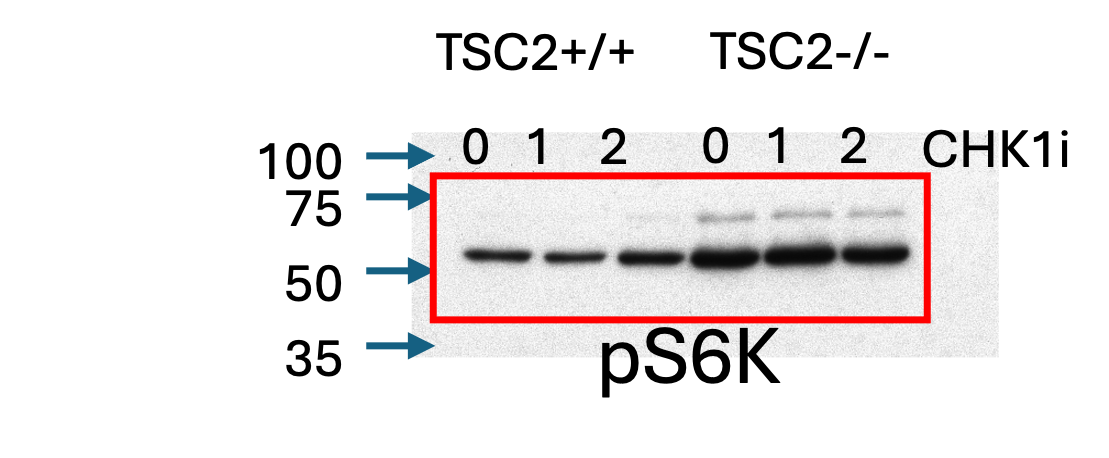

Supplement: Supplementary file 2 — Source data Fig. 1 [file 44319_2025_451_MOESM2_ESM.zip › Figure 1/Figure 1F/Western pS6K.tif]

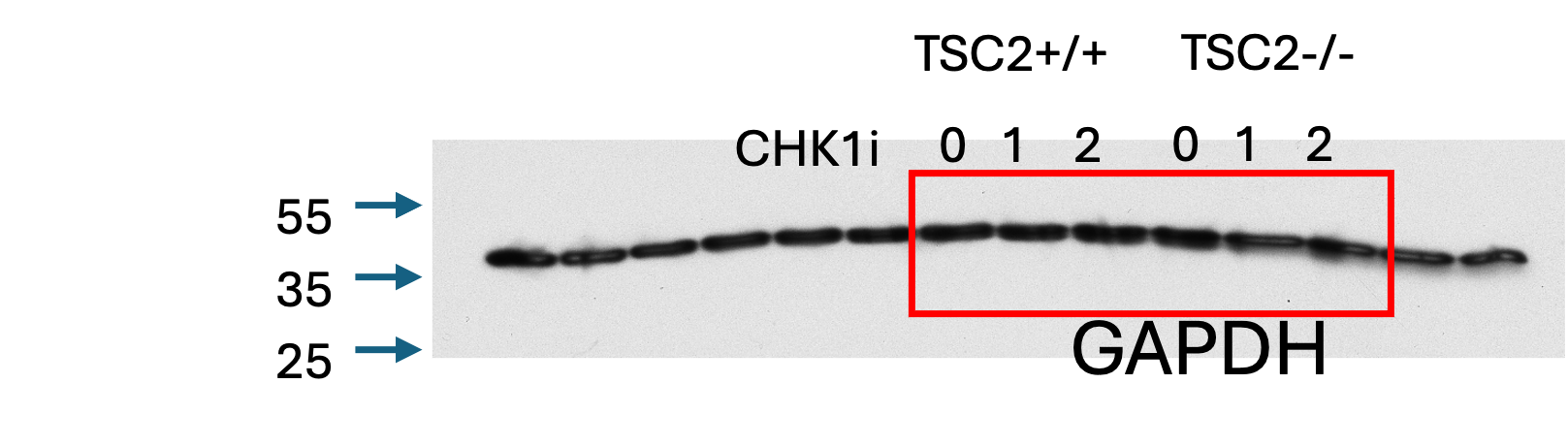

Supplement: Supplementary file 2 — Source data Fig. 1 [file 44319_2025_451_MOESM2_ESM.zip › Figure 1/Figure 1F/Western GAPDH.tif]

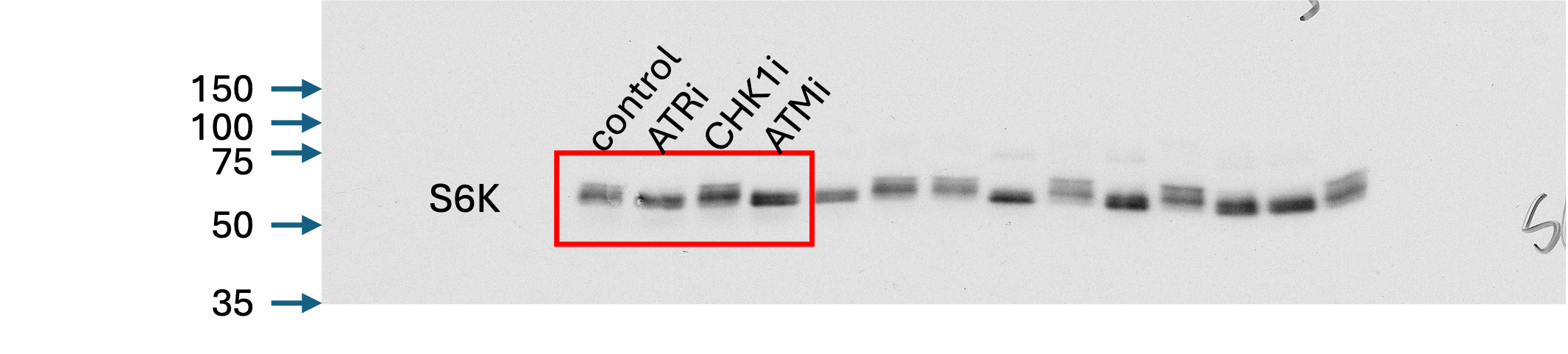

Supplement: Supplementary file 2 — Source data Fig. 1 [file 44319_2025_451_MOESM2_ESM.zip › Figure 1/Figure 1A/Western S6K.tif]

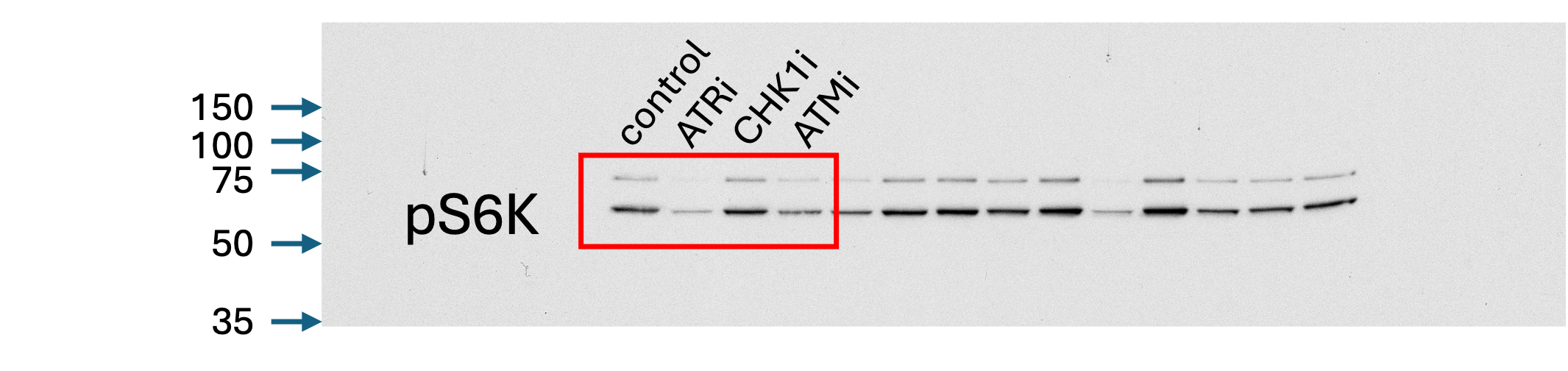

Supplement: Supplementary file 2 — Source data Fig. 1 [file 44319_2025_451_MOESM2_ESM.zip › Figure 1/Figure 1A/Western pS6K.tif]

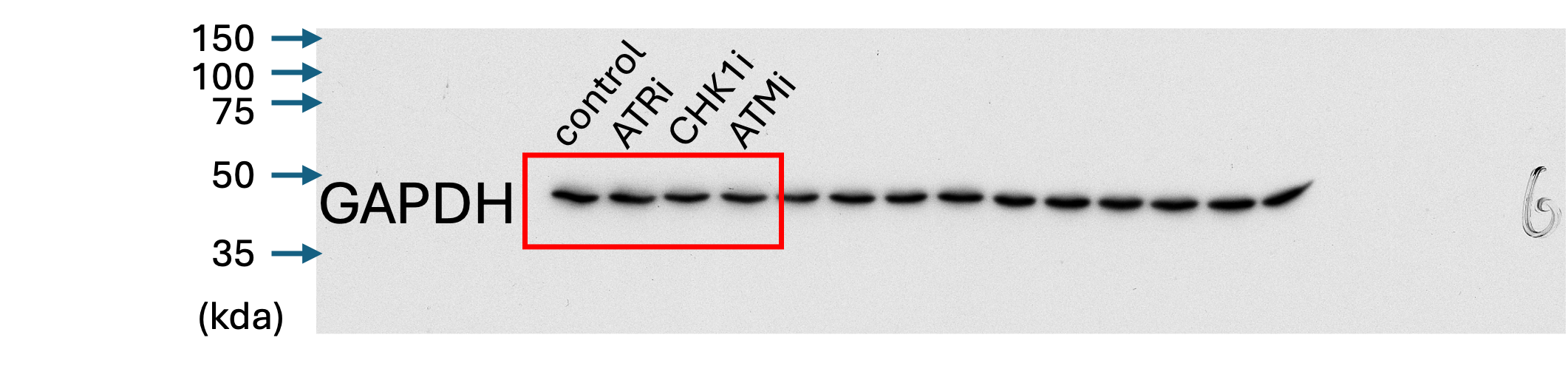

Supplement: Supplementary file 2 — Source data Fig. 1 [file 44319_2025_451_MOESM2_ESM.zip › Figure 1/Figure 1A/Western GAPDH.tif]

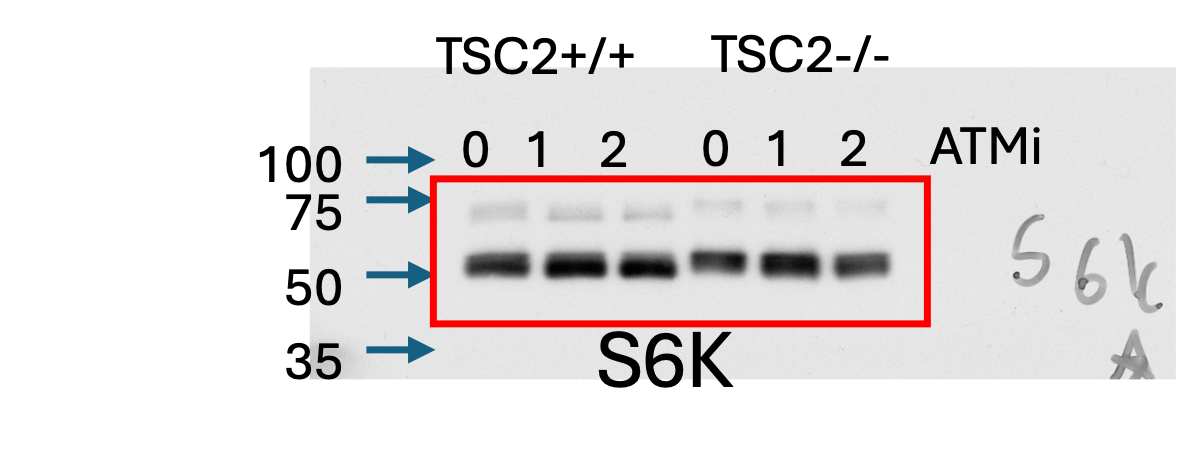

Supplement: Supplementary file 2 — Source data Fig. 1 [file 44319_2025_451_MOESM2_ESM.zip › Figure 1/Figure 1G/Western S6K.tif]

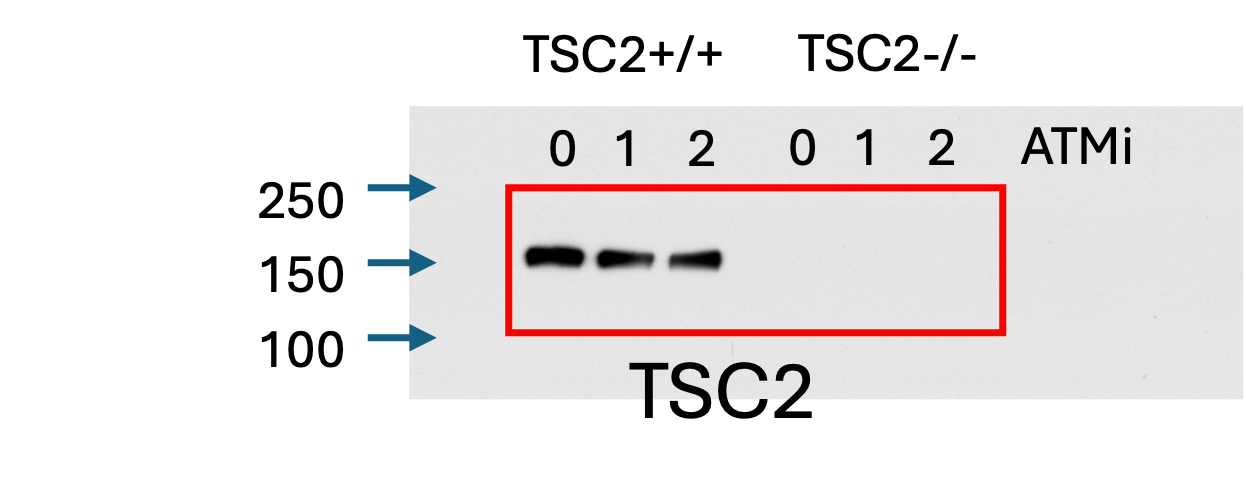

Supplement: Supplementary file 2 — Source data Fig. 1 [file 44319_2025_451_MOESM2_ESM.zip › Figure 1/Figure 1G/Western TSC2.tif]

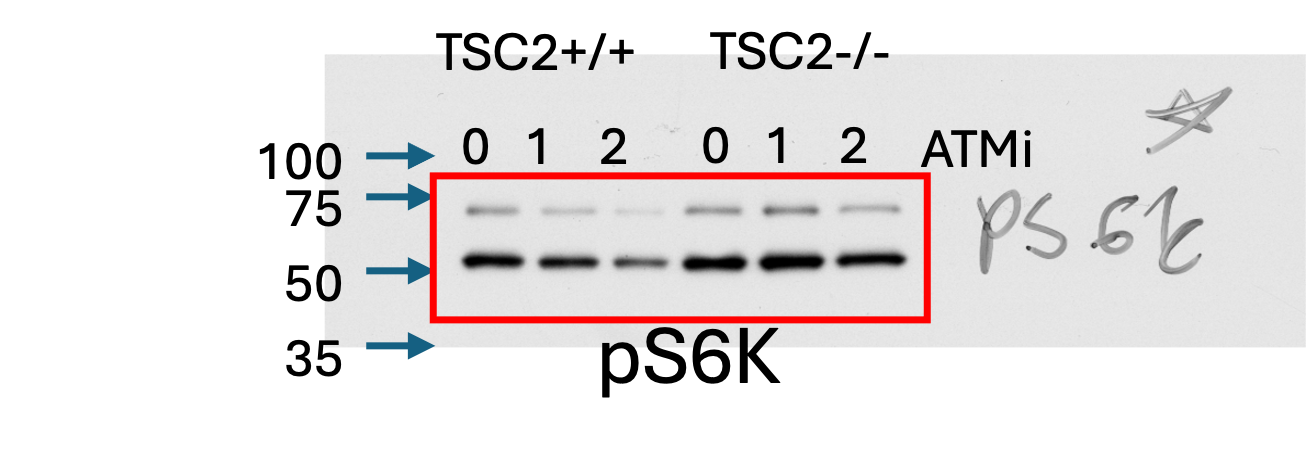

Supplement: Supplementary file 2 — Source data Fig. 1 [file 44319_2025_451_MOESM2_ESM.zip › Figure 1/Figure 1G/Western pS6K.tif]

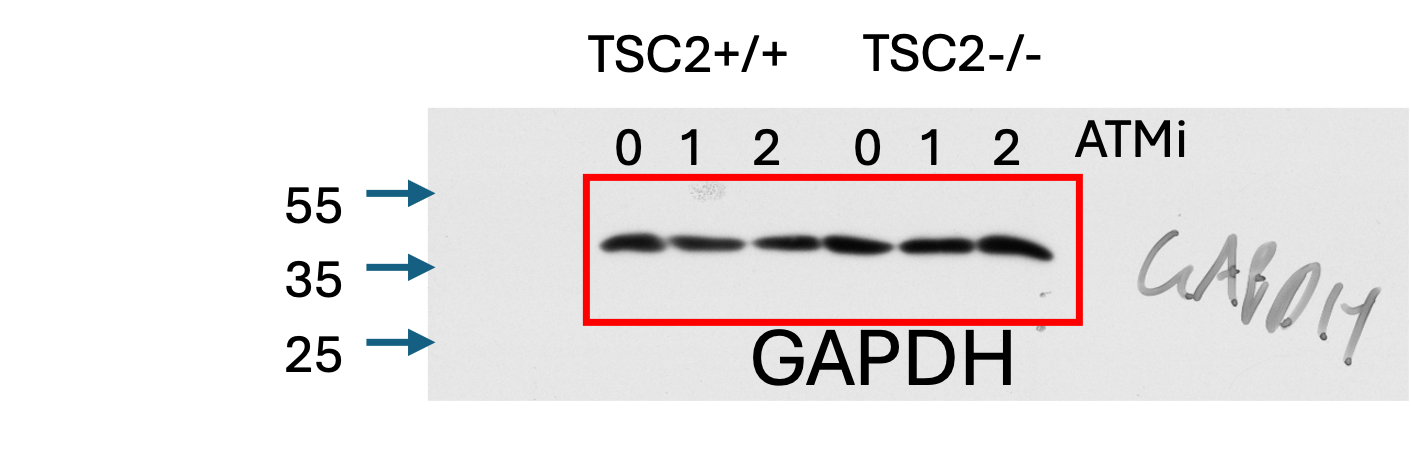

Supplement: Supplementary file 2 — Source data Fig. 1 [file 44319_2025_451_MOESM2_ESM.zip › Figure 1/Figure 1G/Western GAPDH.tif]

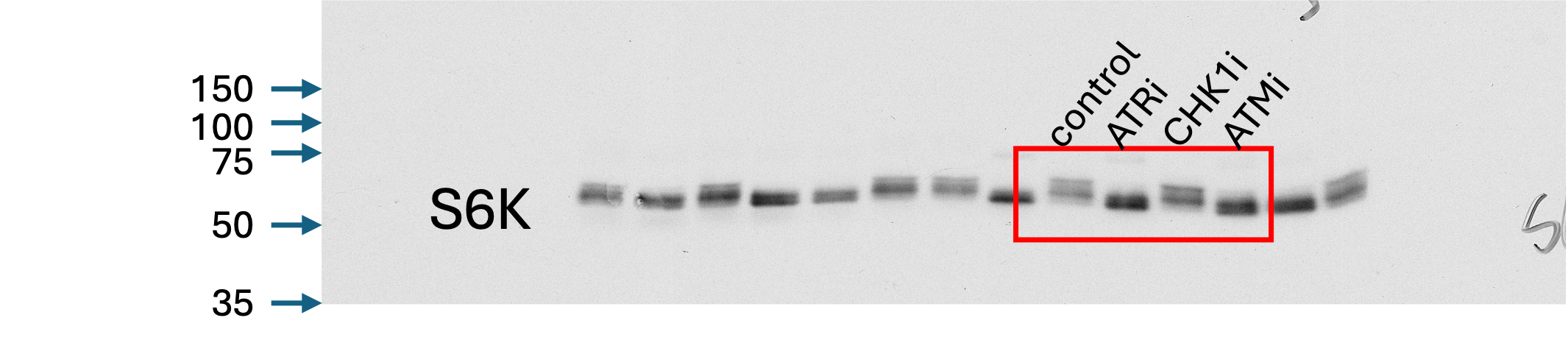

Supplement: Supplementary file 2 — Source data Fig. 1 [file 44319_2025_451_MOESM2_ESM.zip › Figure 1/Figure 1B/Western S6K.tif]

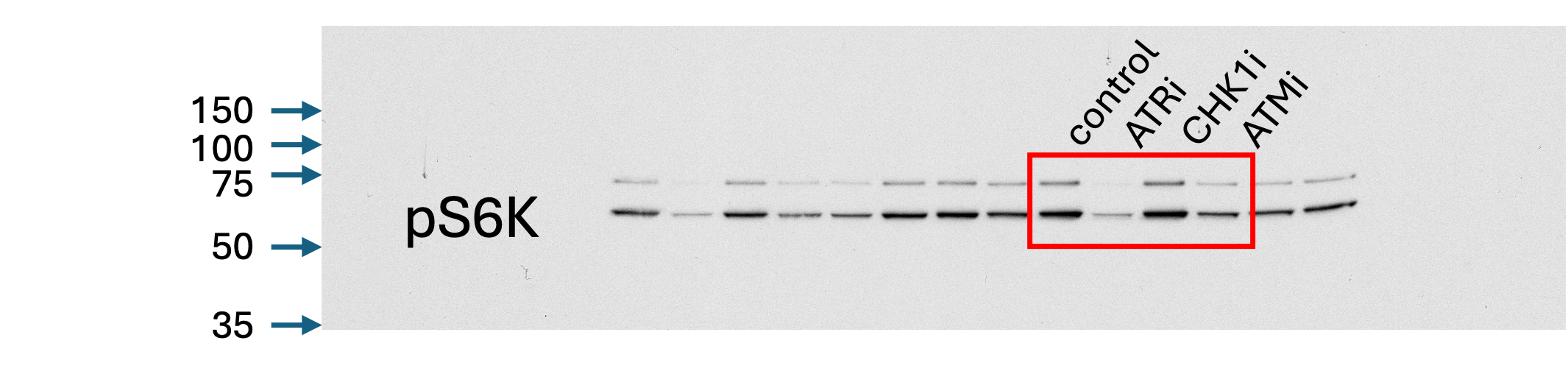

Supplement: Supplementary file 2 — Source data Fig. 1 [file 44319_2025_451_MOESM2_ESM.zip › Figure 1/Figure 1B/Western pS6K.tif]

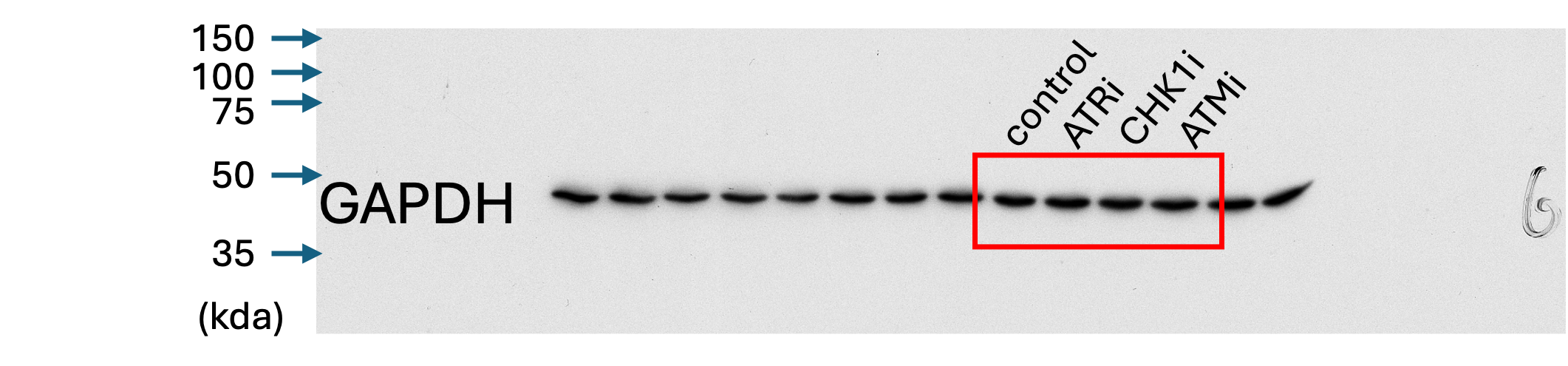

Supplement: Supplementary file 2 — Source data Fig. 1 [file 44319_2025_451_MOESM2_ESM.zip › Figure 1/Figure 1B/Western GAPDH.tif]

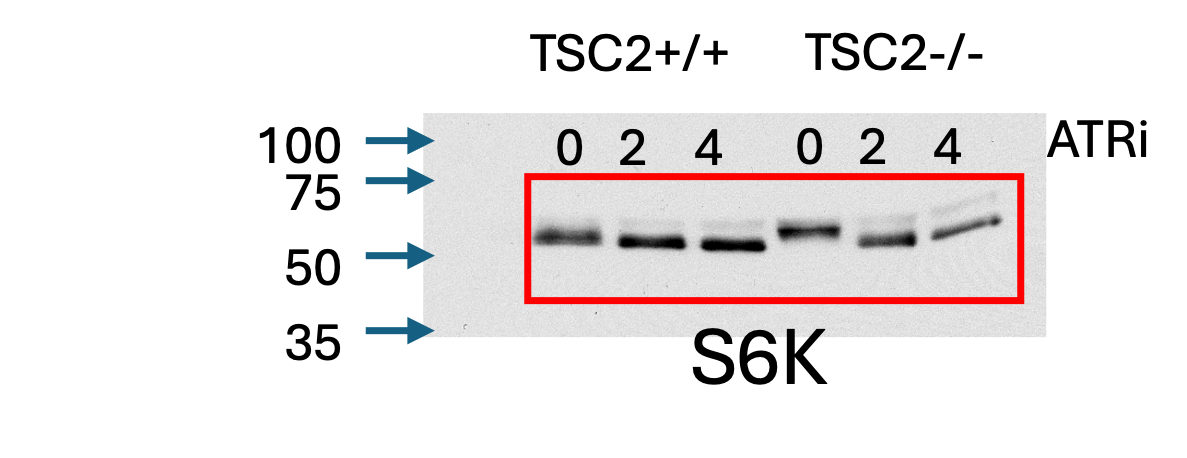

Supplement: Supplementary file 2 — Source data Fig. 1 [file 44319_2025_451_MOESM2_ESM.zip › Figure 1/Figure 1E/Western S6K.tif]

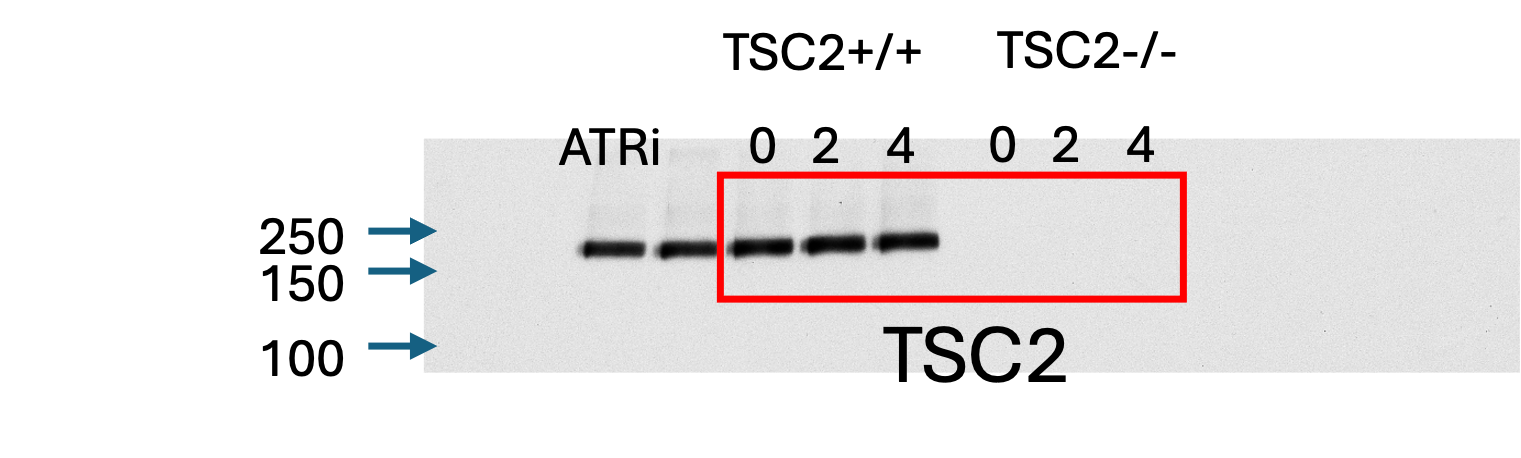

Supplement: Supplementary file 2 — Source data Fig. 1 [file 44319_2025_451_MOESM2_ESM.zip › Figure 1/Figure 1E/Western TSC2.tif]

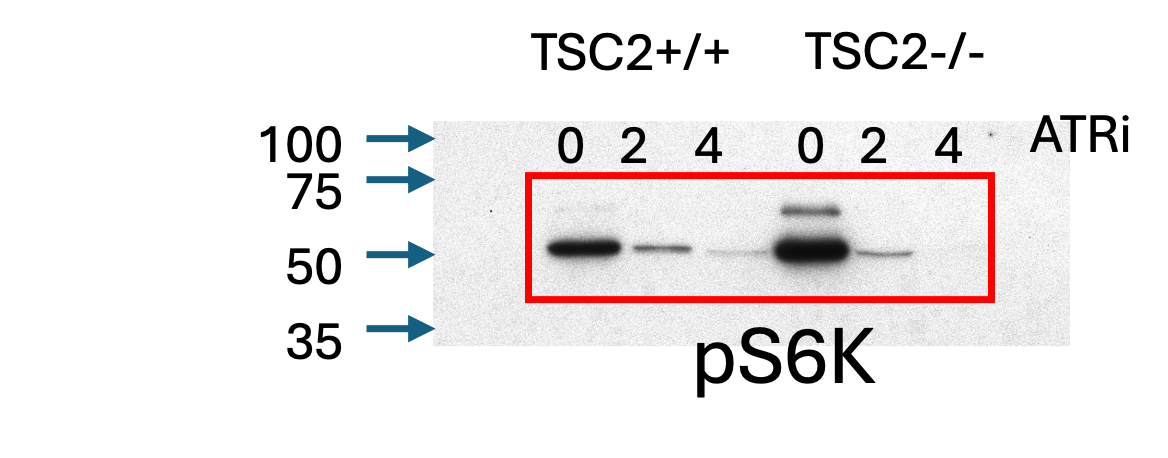

Supplement: Supplementary file 2 — Source data Fig. 1 [file 44319_2025_451_MOESM2_ESM.zip › Figure 1/Figure 1E/Western pS6K.tif]

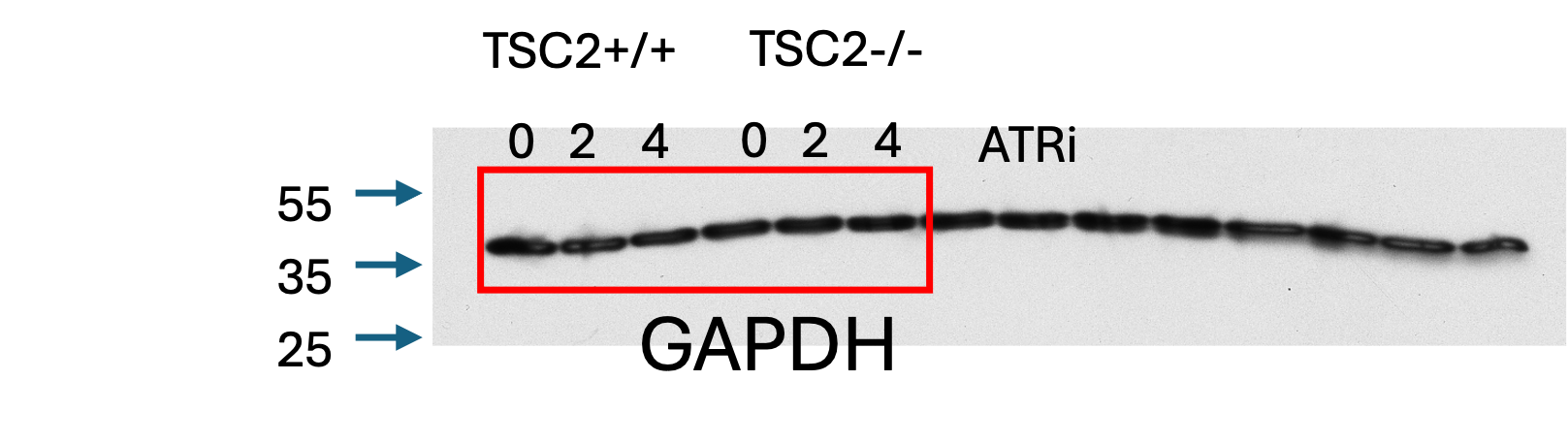

Supplement: Supplementary file 2 — Source data Fig. 1 [file 44319_2025_451_MOESM2_ESM.zip › Figure 1/Figure 1E/Western GAPDH.tif]

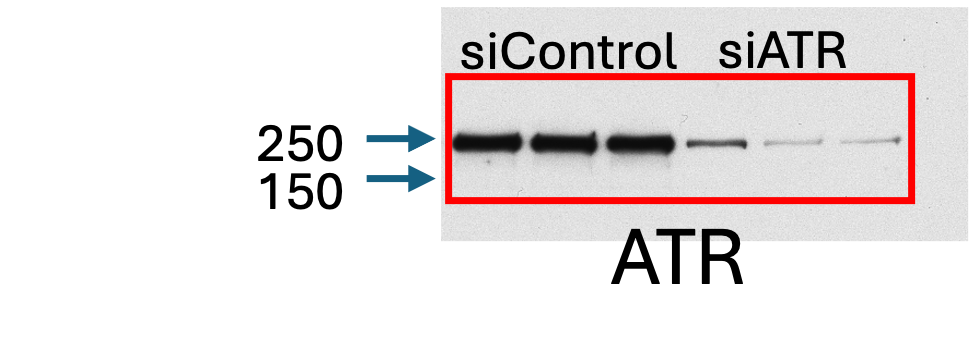

Supplement: Supplementary file 2 — Source data Fig. 1 [file 44319_2025_451_MOESM2_ESM.zip › Figure 1/Figure 1D/Western ATR.tif]

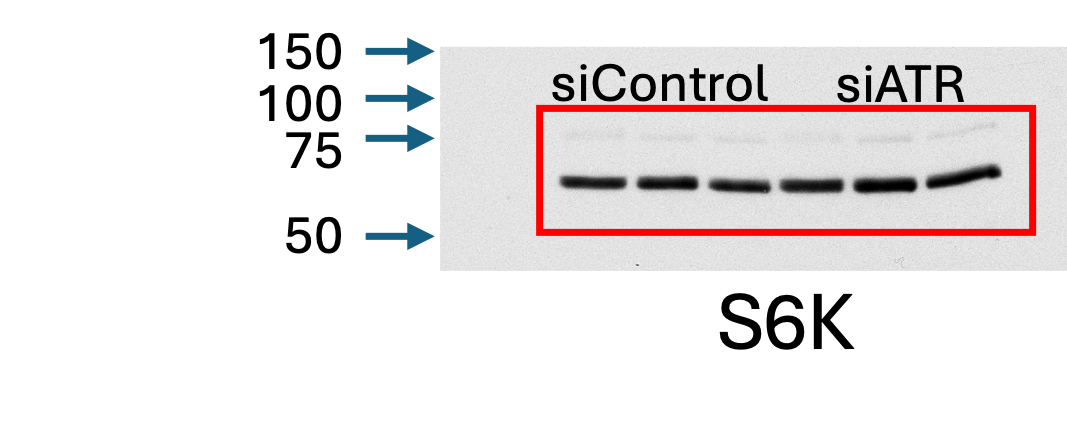

Supplement: Supplementary file 2 — Source data Fig. 1 [file 44319_2025_451_MOESM2_ESM.zip › Figure 1/Figure 1D/Western S6K.tif]

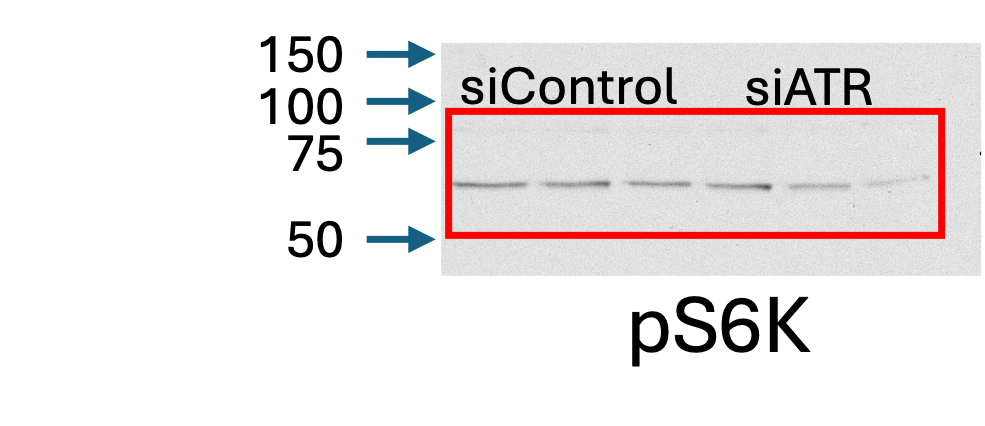

Supplement: Supplementary file 2 — Source data Fig. 1 [file 44319_2025_451_MOESM2_ESM.zip › Figure 1/Figure 1D/Western pS6K.tif]

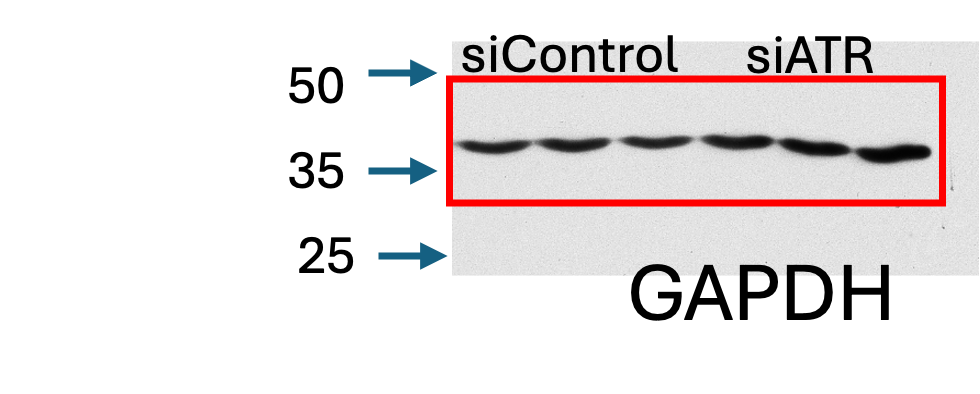

Supplement: Supplementary file 2 — Source data Fig. 1 [file 44319_2025_451_MOESM2_ESM.zip › Figure 1/Figure 1D/Western GAPDH.tif]

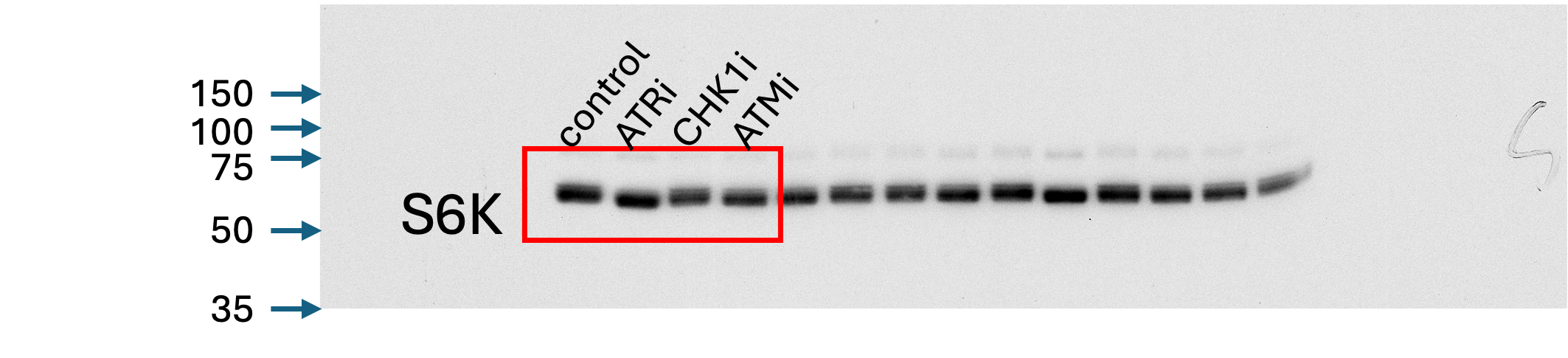

Supplement: Supplementary file 2 — Source data Fig. 1 [file 44319_2025_451_MOESM2_ESM.zip › Figure 1/Figure 1C/Western S6K.tif]

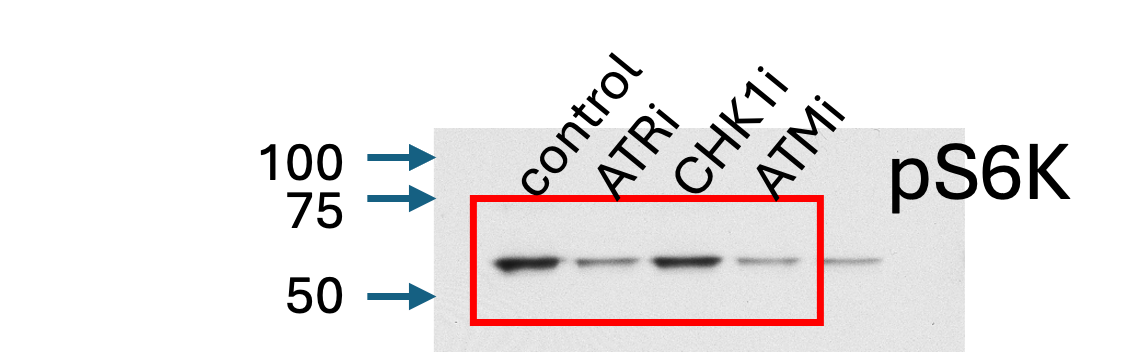

Supplement: Supplementary file 2 — Source data Fig. 1 [file 44319_2025_451_MOESM2_ESM.zip › Figure 1/Figure 1C/Western pS6K.tif]

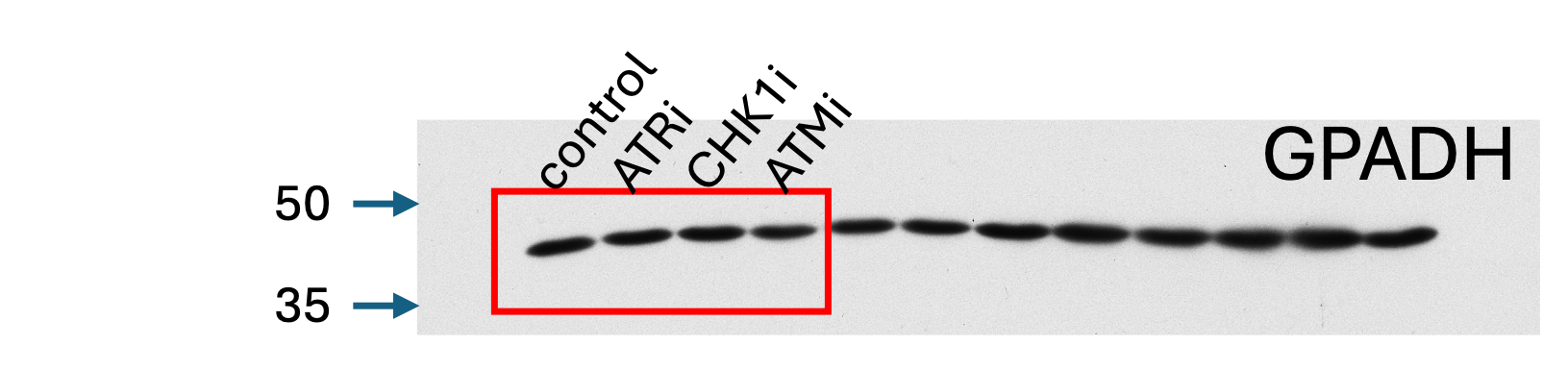

Supplement: Supplementary file 2 — Source data Fig. 1 [file 44319_2025_451_MOESM2_ESM.zip › Figure 1/Figure 1C/Western GAPDH.tif]

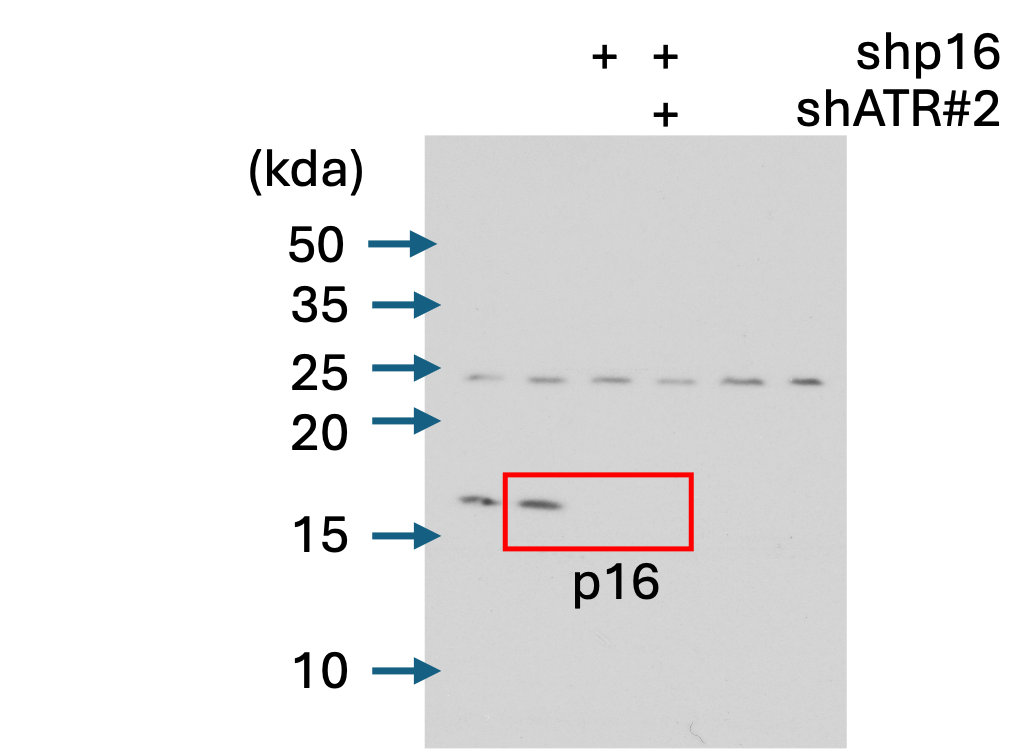

Supplement: Supplementary file 3 — Source data Fig. 2 [file 44319_2025_451_MOESM3_ESM.zip › Figure 2/Figure 2A/western shATR#2 p16.tif]

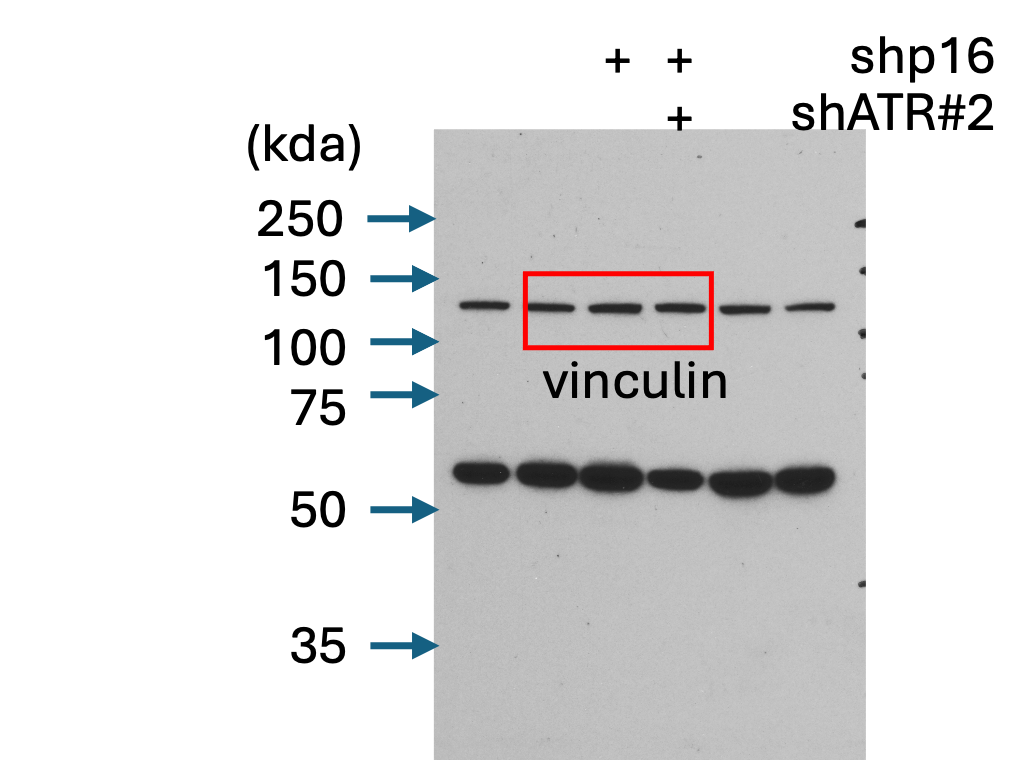

Supplement: Supplementary file 3 — Source data Fig. 2 [file 44319_2025_451_MOESM3_ESM.zip › Figure 2/Figure 2A/western shATR#2 vinculin.tif]

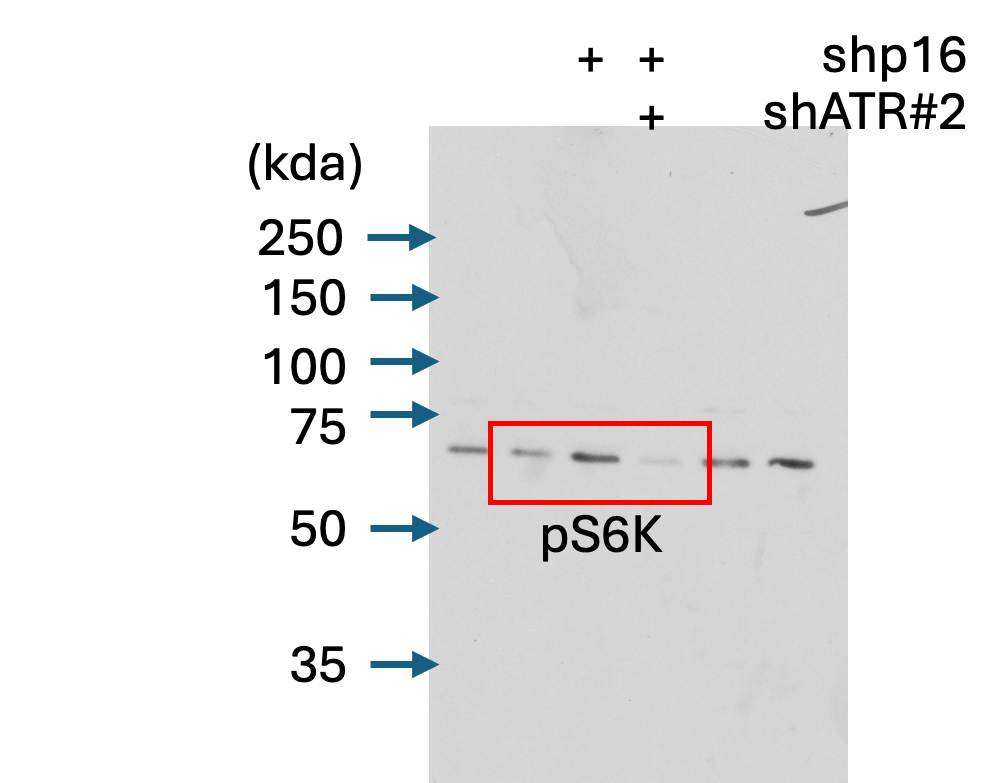

Supplement: Supplementary file 3 — Source data Fig. 2 [file 44319_2025_451_MOESM3_ESM.zip › Figure 2/Figure 2A/western shATR#2 pS6K.tif]

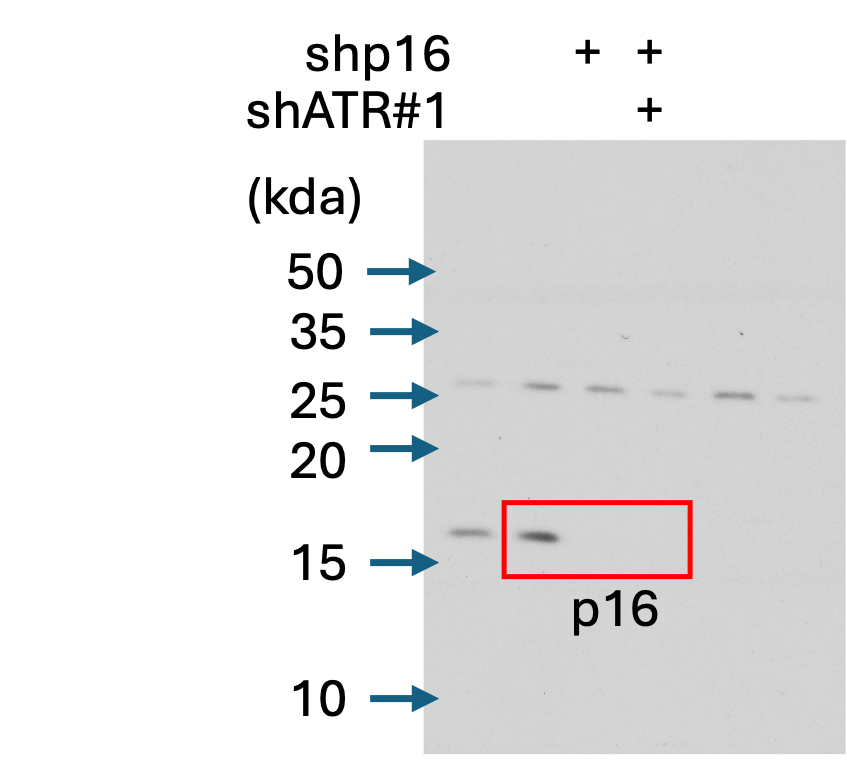

Supplement: Supplementary file 3 — Source data Fig. 2 [file 44319_2025_451_MOESM3_ESM.zip › Figure 2/Figure 2A/western shATR#1 p16.tif]

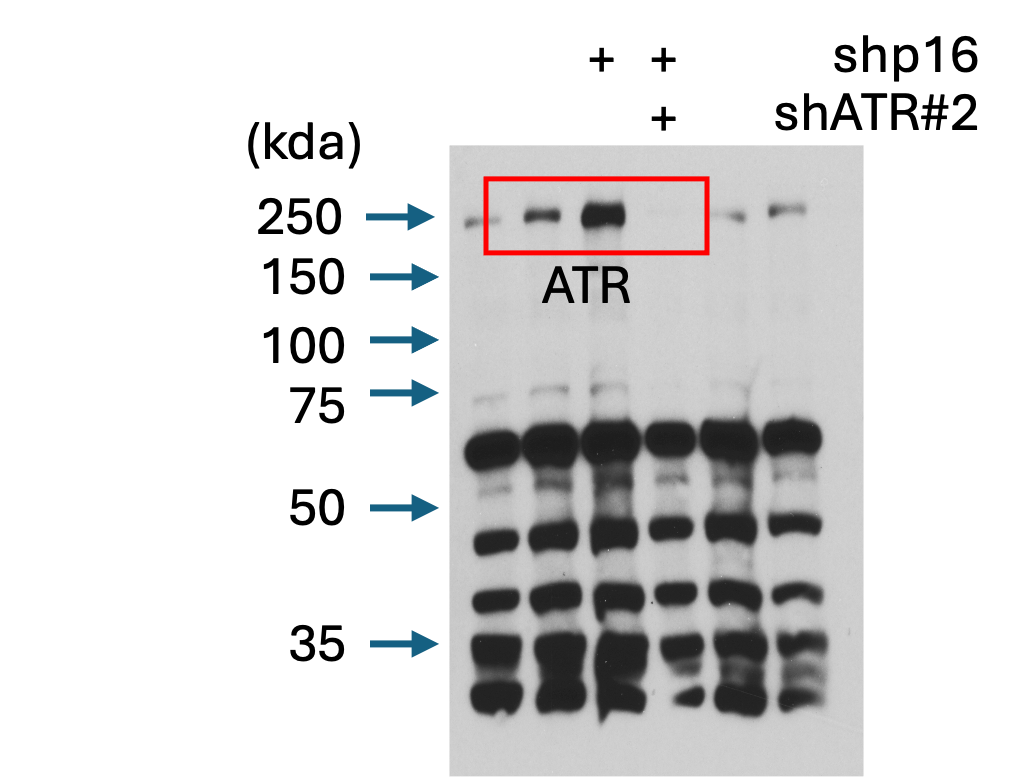

Supplement: Supplementary file 3 — Source data Fig. 2 [file 44319_2025_451_MOESM3_ESM.zip › Figure 2/Figure 2A/western shATR#2 ATR.tif]

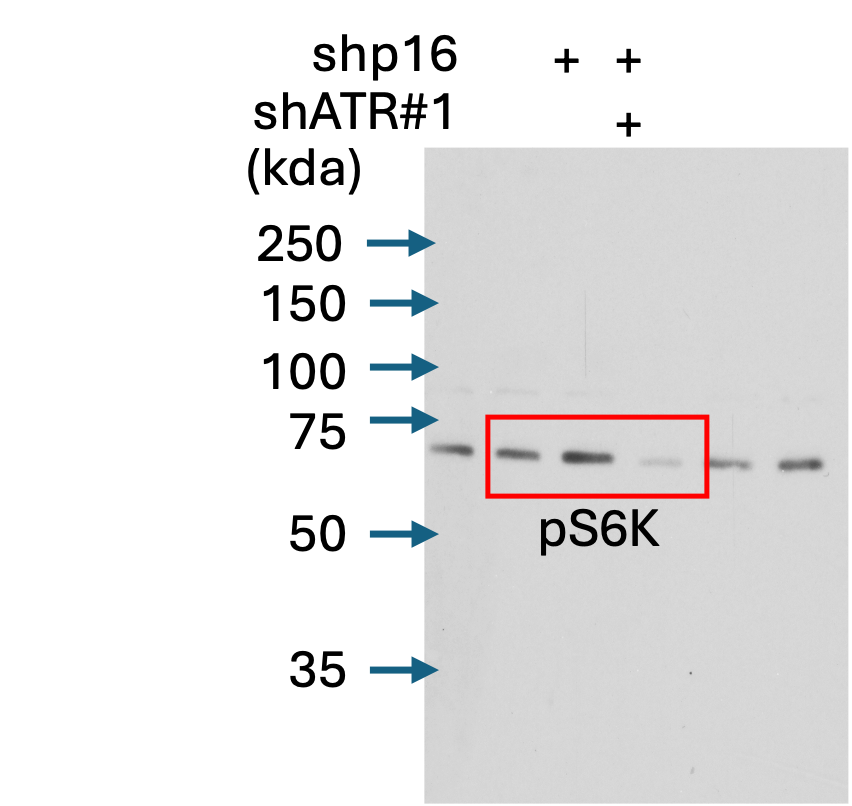

Supplement: Supplementary file 3 — Source data Fig. 2 [file 44319_2025_451_MOESM3_ESM.zip › Figure 2/Figure 2A/western shATR#1 pS6K.tif]

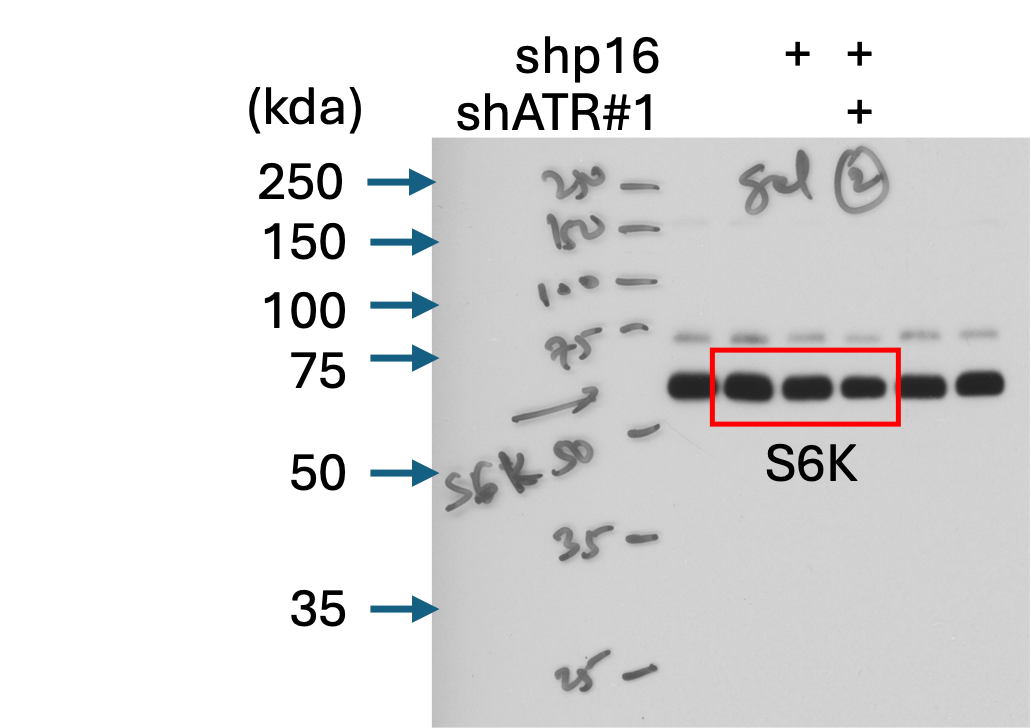

Supplement: Supplementary file 3 — Source data Fig. 2 [file 44319_2025_451_MOESM3_ESM.zip › Figure 2/Figure 2A/western shATR#1 S6K.tif]

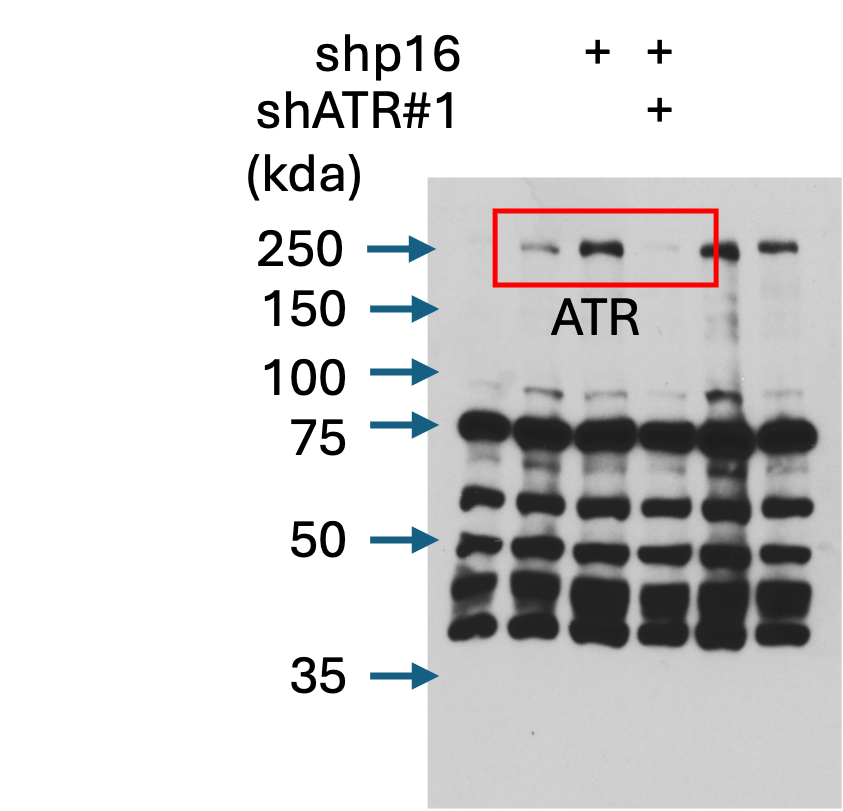

Supplement: Supplementary file 3 — Source data Fig. 2 [file 44319_2025_451_MOESM3_ESM.zip › Figure 2/Figure 2A/western shATR#1 ATR.tif]

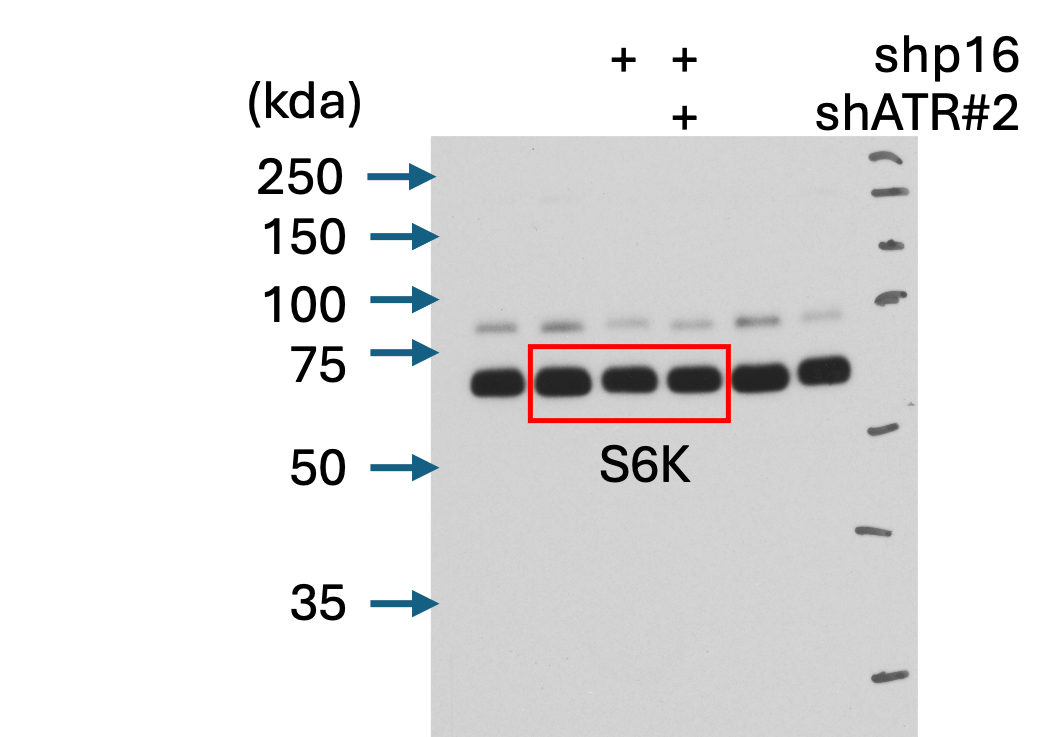

Supplement: Supplementary file 3 — Source data Fig. 2 [file 44319_2025_451_MOESM3_ESM.zip › Figure 2/Figure 2A/western shATR#2 S6K.tif]

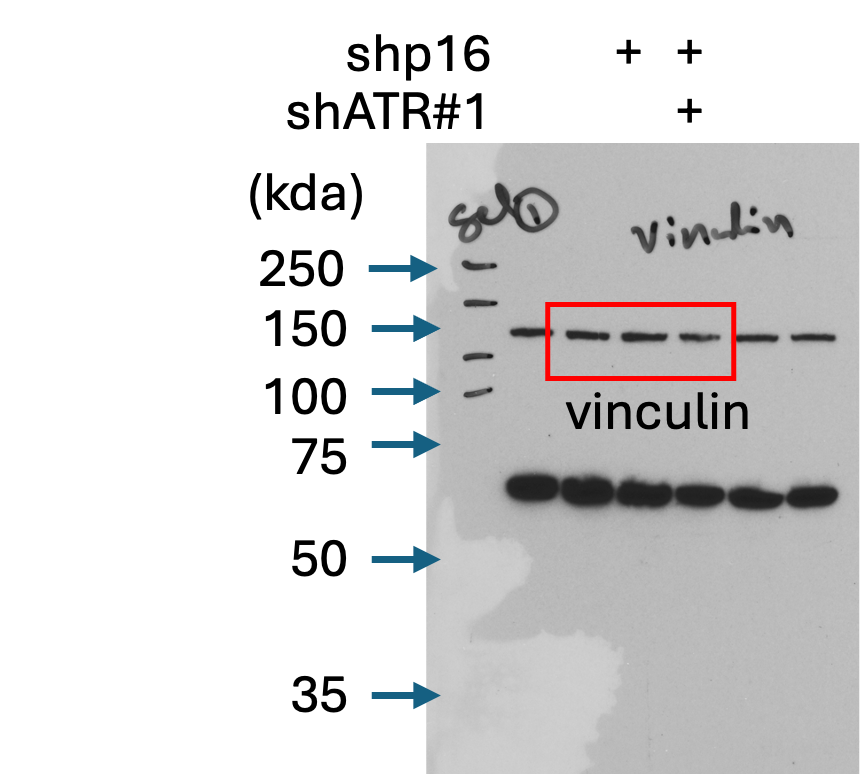

Supplement: Supplementary file 3 — Source data Fig. 2 [file 44319_2025_451_MOESM3_ESM.zip › Figure 2/Figure 2A/western shATR#1 vinculin.tif]

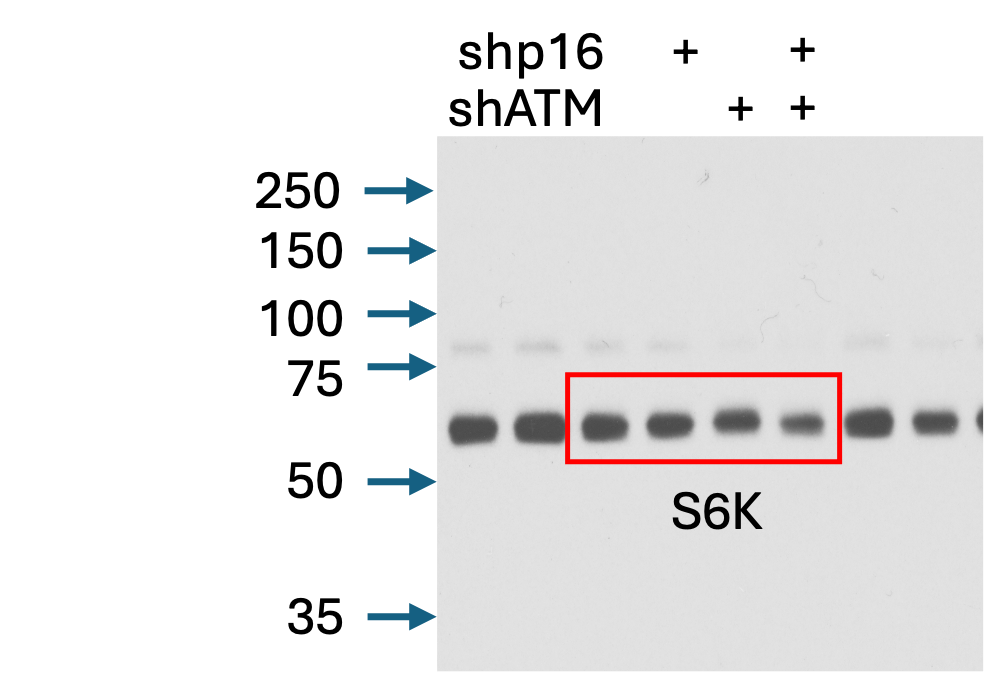

Supplement: Supplementary file 3 — Source data Fig. 2 [file 44319_2025_451_MOESM3_ESM.zip › Figure 2/Figure 2C/western S6K.tif]

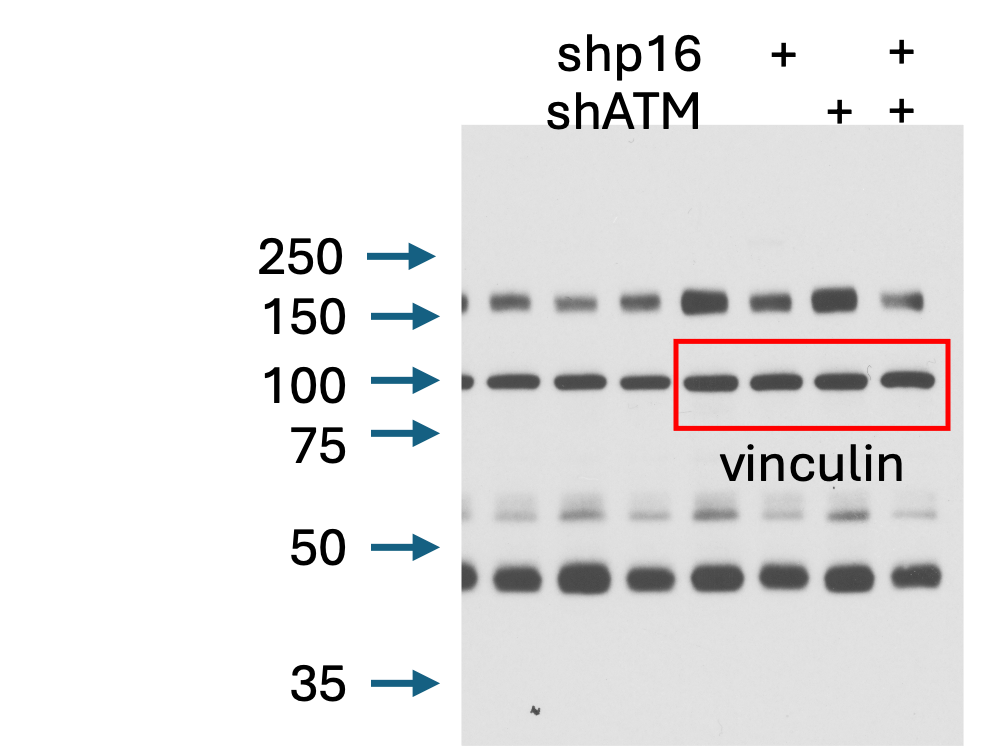

Supplement: Supplementary file 3 — Source data Fig. 2 [file 44319_2025_451_MOESM3_ESM.zip › Figure 2/Figure 2C/western vinculin.tif]

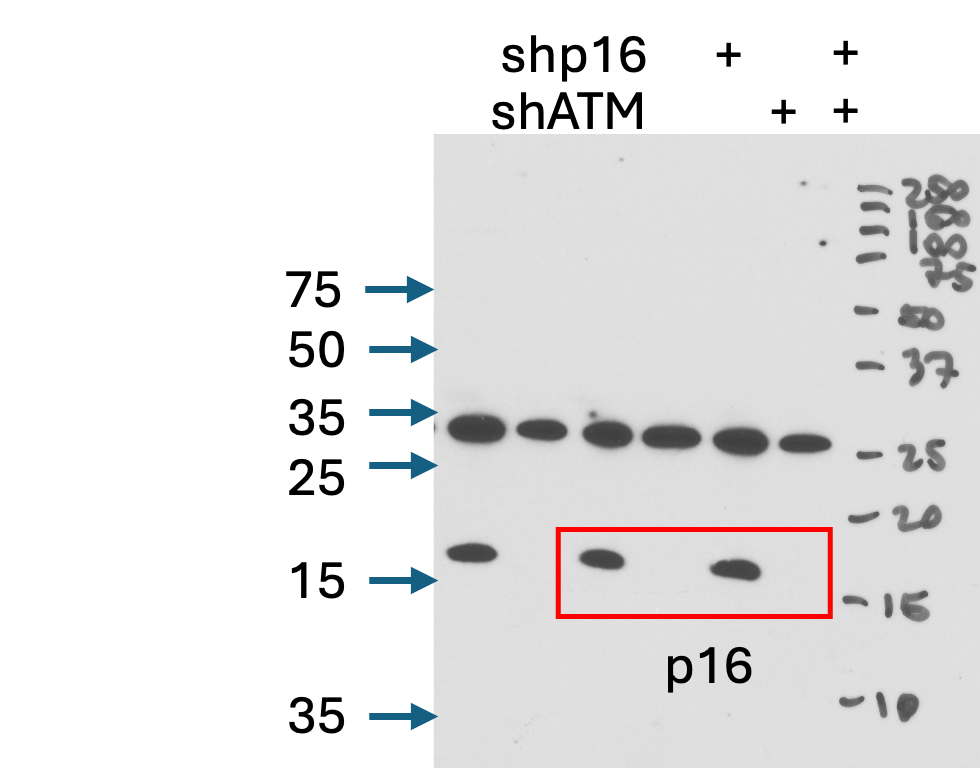

Supplement: Supplementary file 3 — Source data Fig. 2 [file 44319_2025_451_MOESM3_ESM.zip › Figure 2/Figure 2C/western p16.tif]

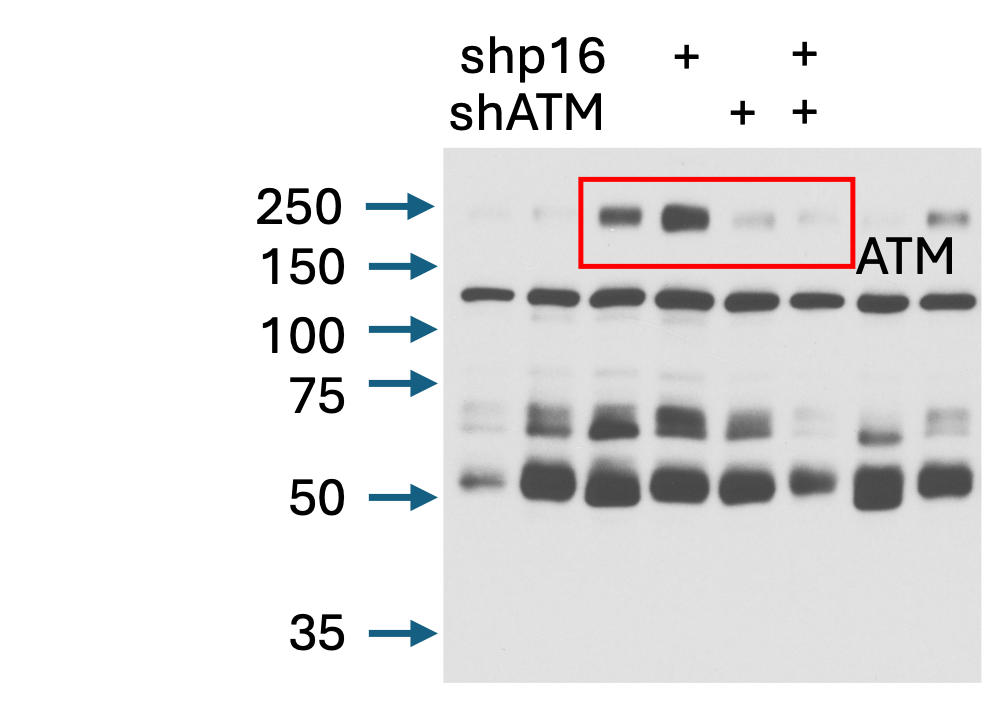

Supplement: Supplementary file 3 — Source data Fig. 2 [file 44319_2025_451_MOESM3_ESM.zip › Figure 2/Figure 2C/western ATM.tif]

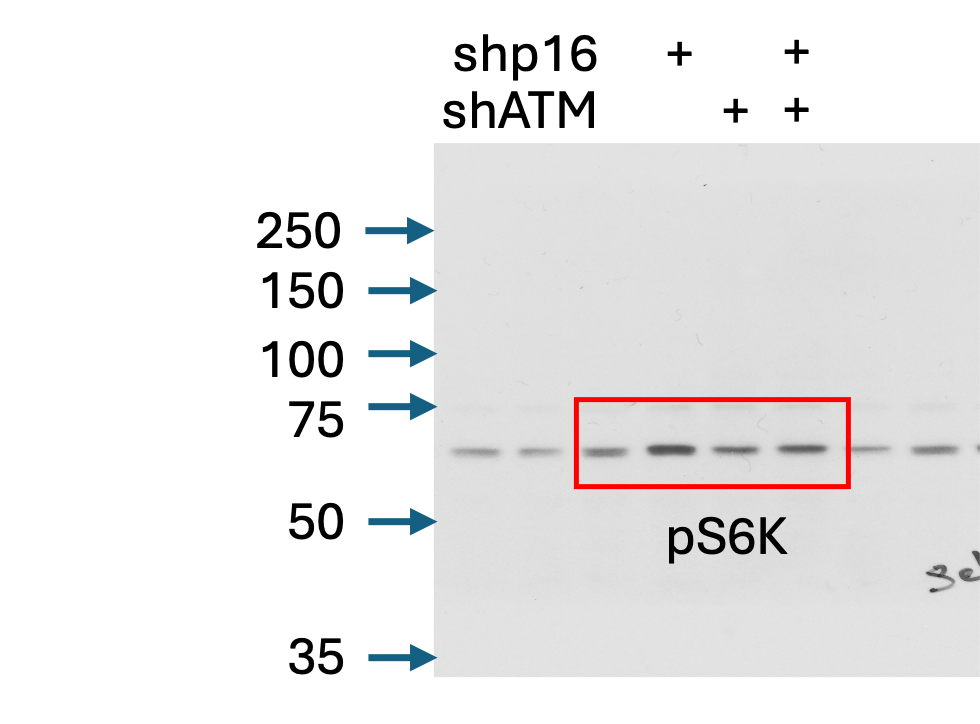

Supplement: Supplementary file 3 — Source data Fig. 2 [file 44319_2025_451_MOESM3_ESM.zip › Figure 2/Figure 2C/western pS6K.tif]

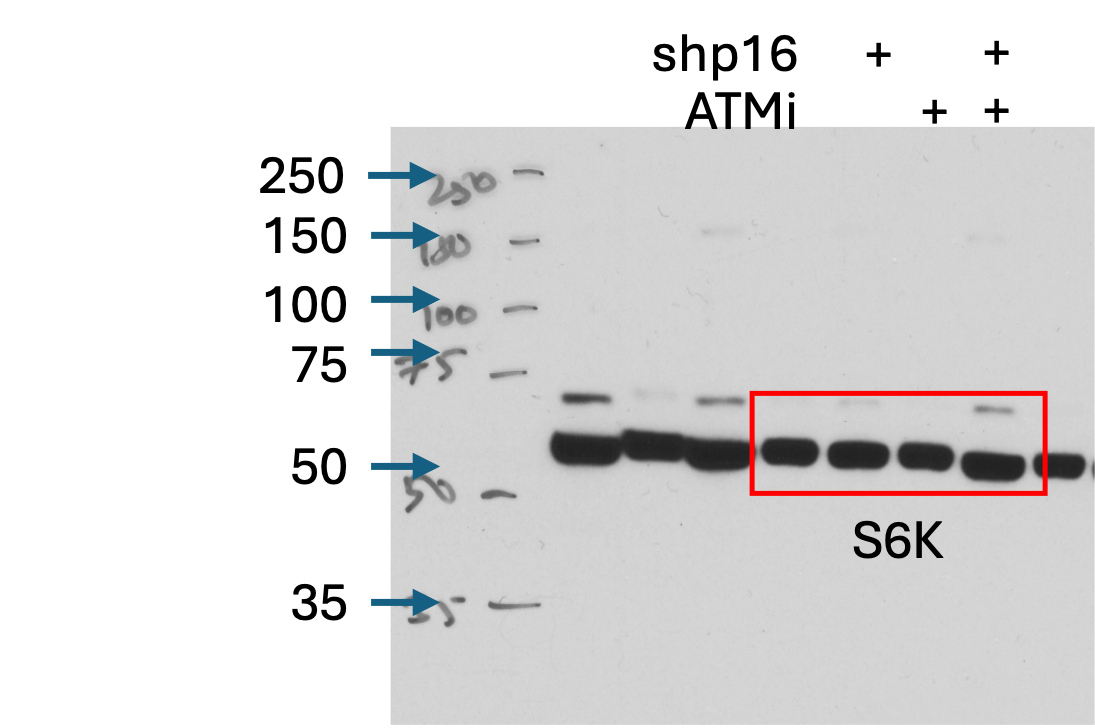

Supplement: Supplementary file 3 — Source data Fig. 2 [file 44319_2025_451_MOESM3_ESM.zip › Figure 2/Figure 2D/western S6K.tif]

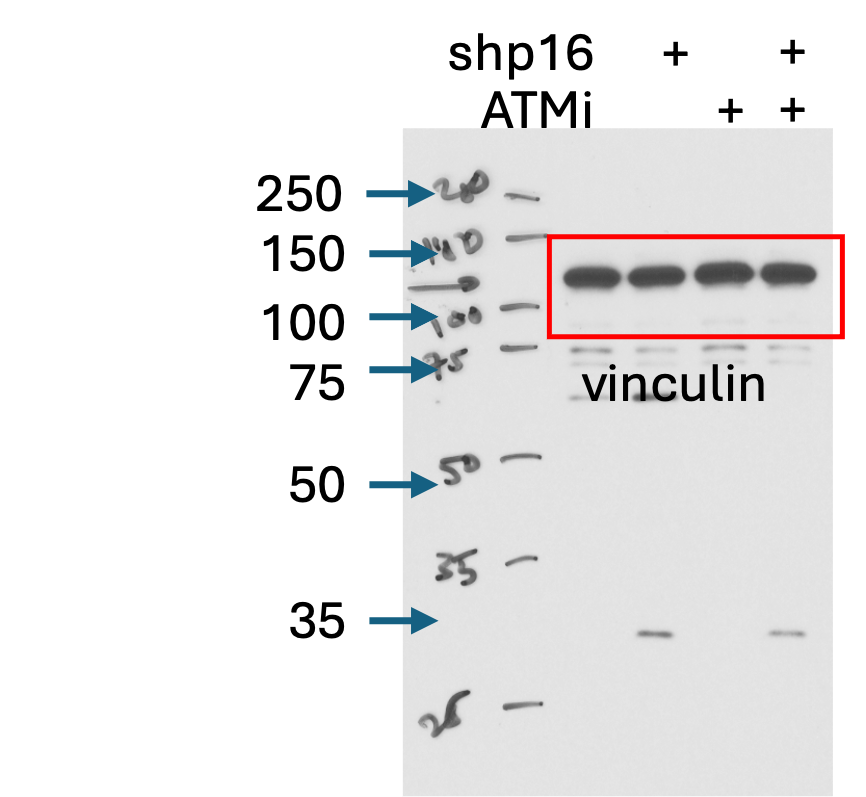

Supplement: Supplementary file 3 — Source data Fig. 2 [file 44319_2025_451_MOESM3_ESM.zip › Figure 2/Figure 2D/western vinculin.tif]

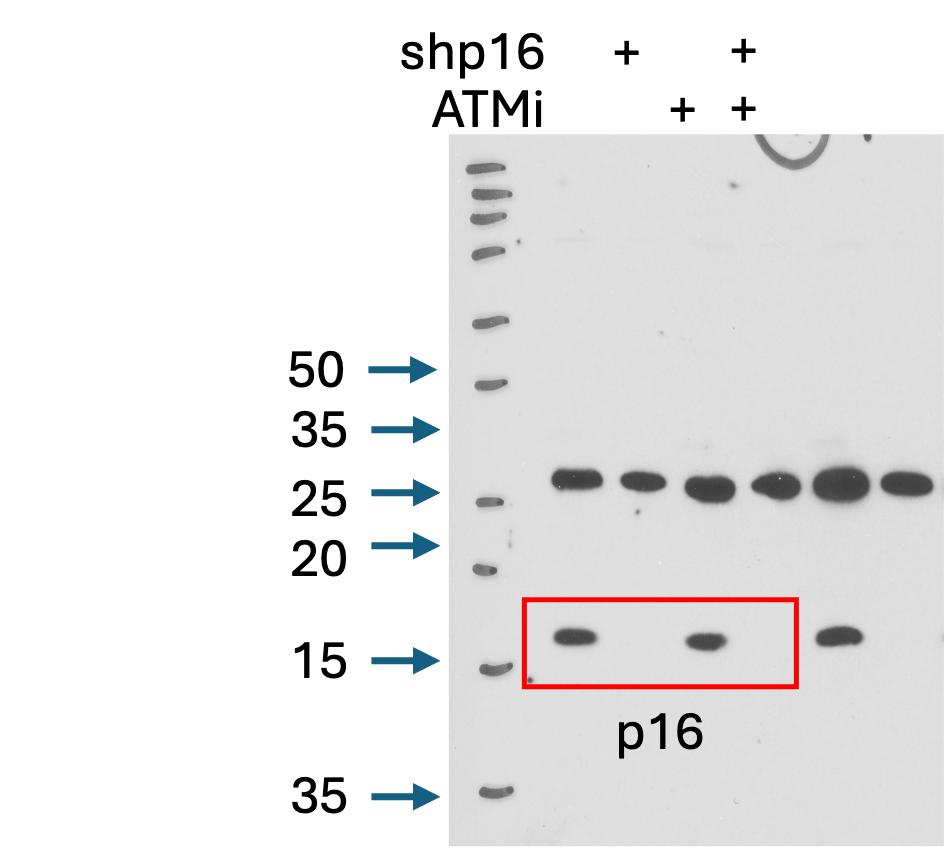

Supplement: Supplementary file 3 — Source data Fig. 2 [file 44319_2025_451_MOESM3_ESM.zip › Figure 2/Figure 2D/western p16.tif]

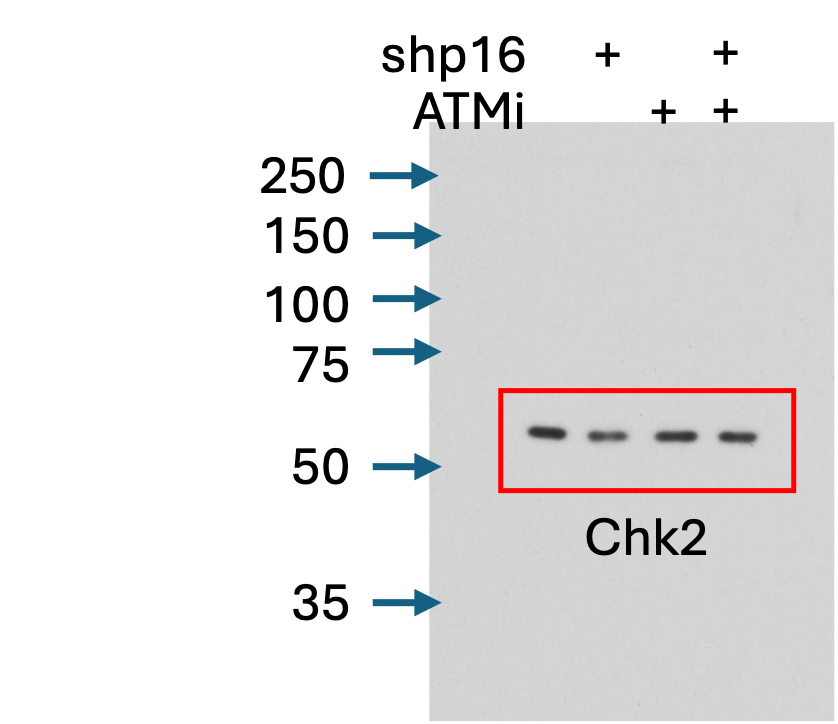

Supplement: Supplementary file 3 — Source data Fig. 2 [file 44319_2025_451_MOESM3_ESM.zip › Figure 2/Figure 2D/western Chk2.tif]

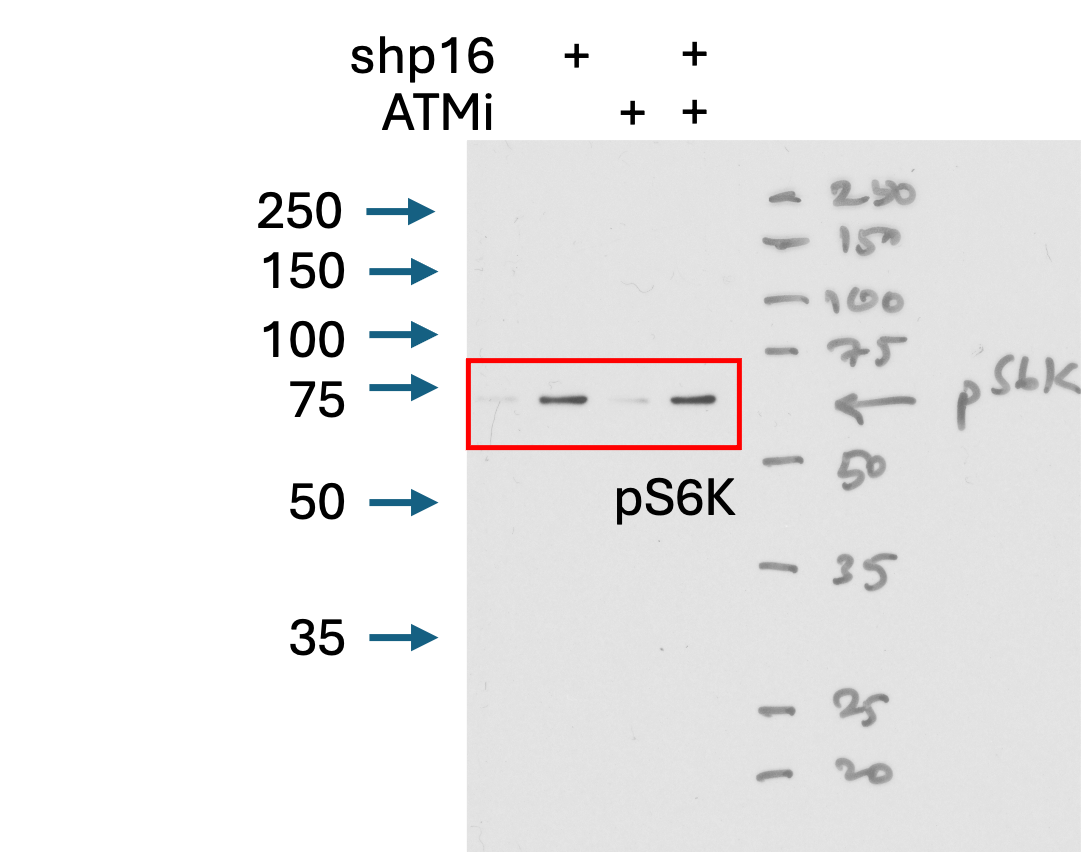

Supplement: Supplementary file 3 — Source data Fig. 2 [file 44319_2025_451_MOESM3_ESM.zip › Figure 2/Figure 2D/western pS6K.tif]

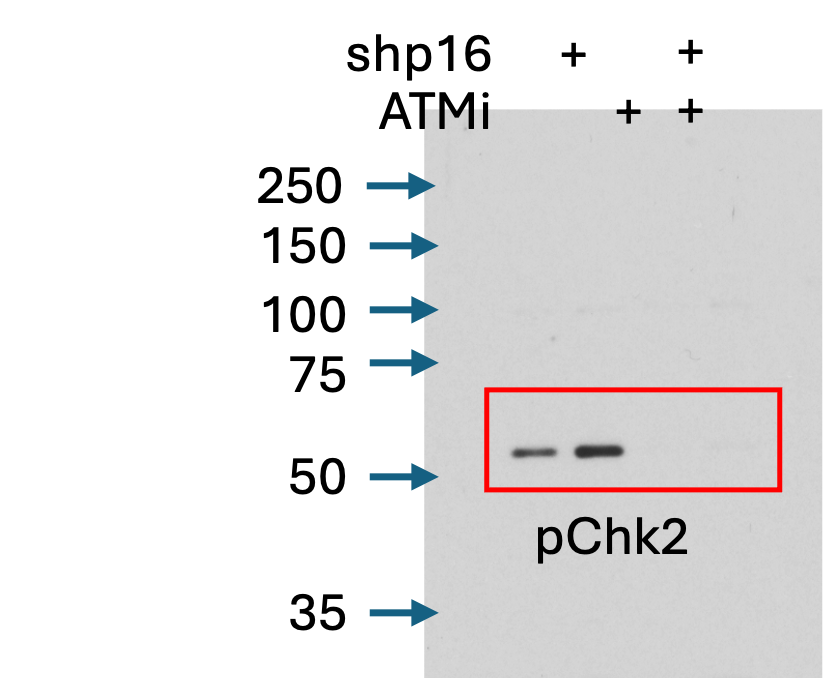

Supplement: Supplementary file 3 — Source data Fig. 2 [file 44319_2025_451_MOESM3_ESM.zip › Figure 2/Figure 2D/western pChk2.tif]

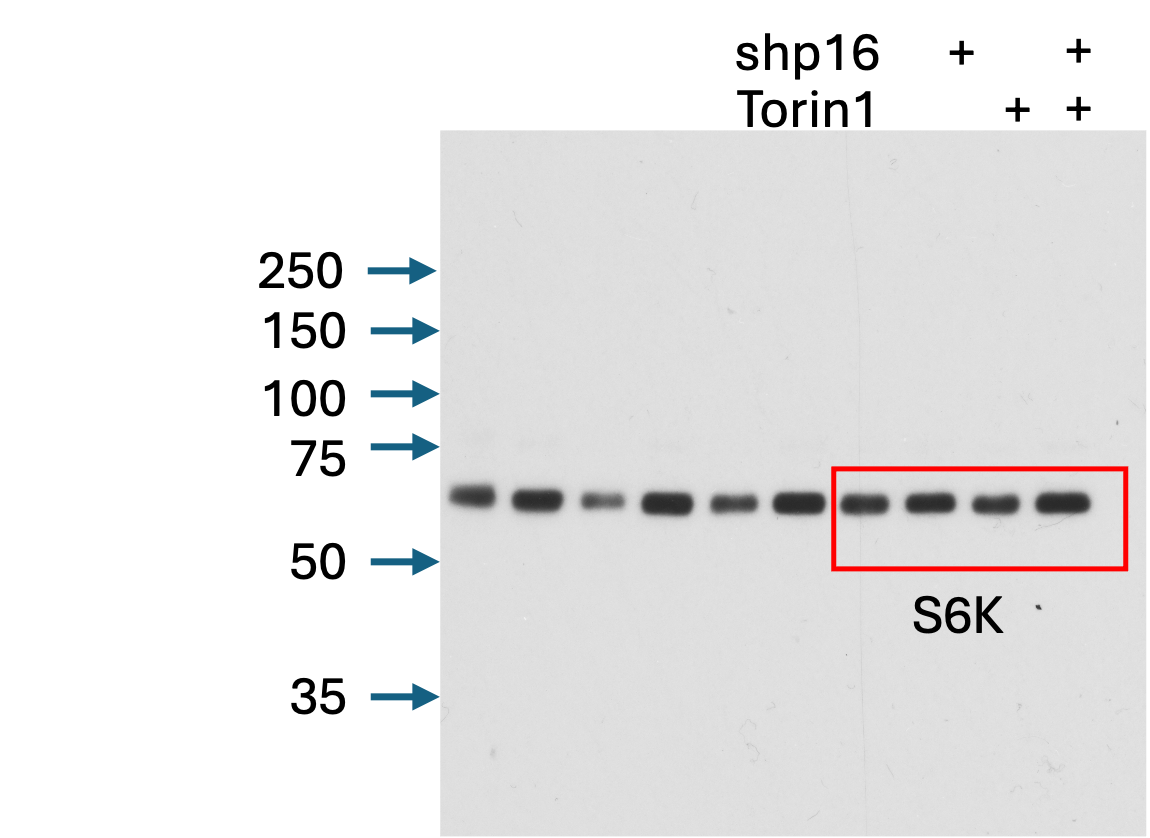

Supplement: Supplementary file 3 — Source data Fig. 2 [file 44319_2025_451_MOESM3_ESM.zip › Figure 2/Figure 2E/western S6K.tif]

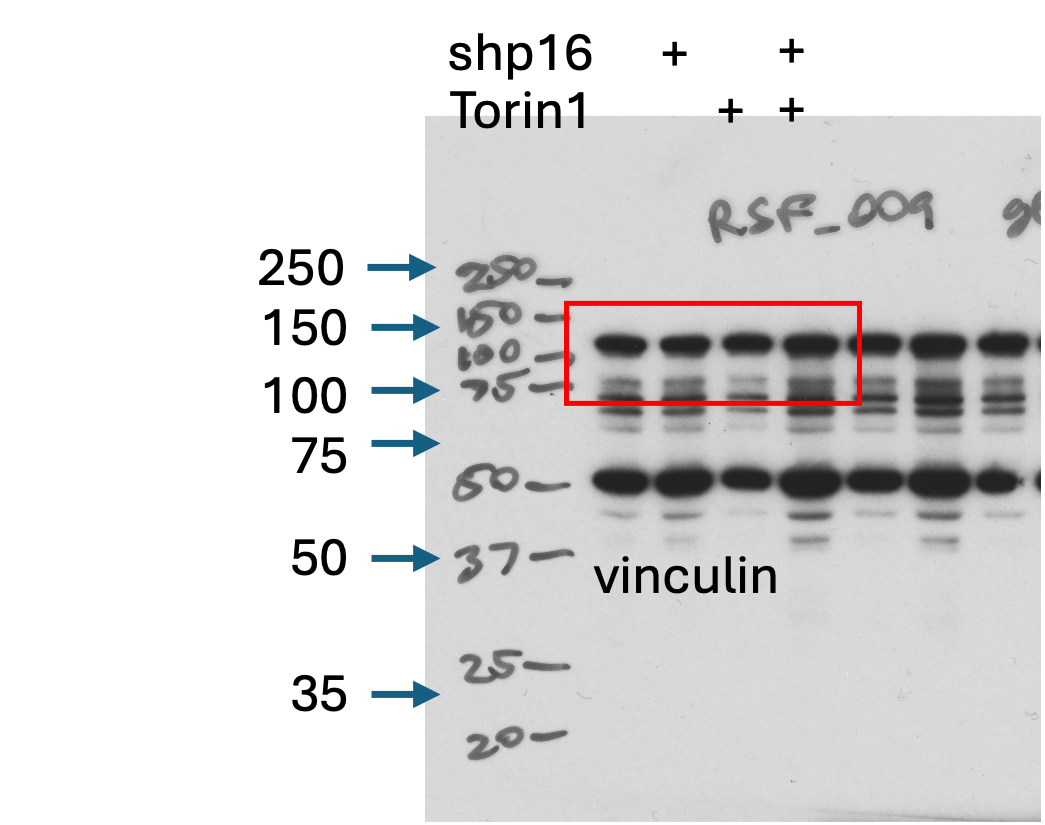

Supplement: Supplementary file 3 — Source data Fig. 2 [file 44319_2025_451_MOESM3_ESM.zip › Figure 2/Figure 2E/western vinculin.tif]

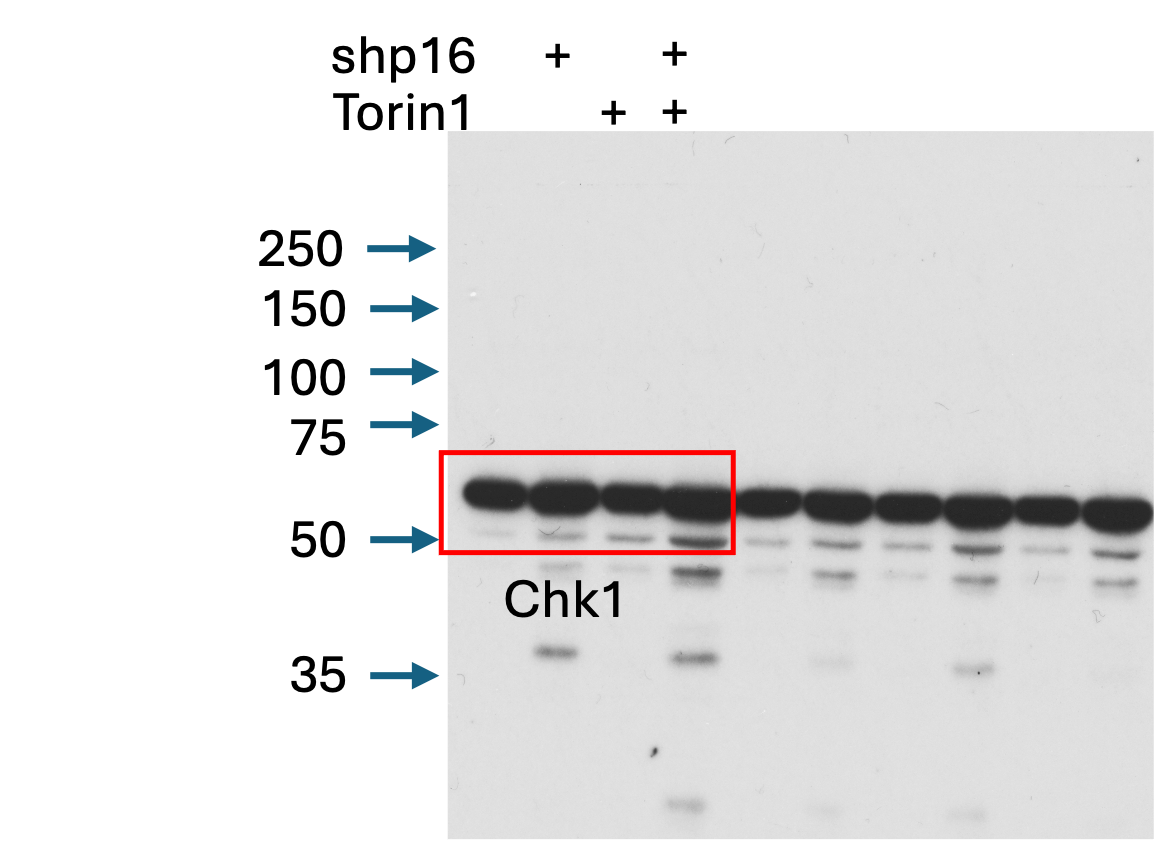

Supplement: Supplementary file 3 — Source data Fig. 2 [file 44319_2025_451_MOESM3_ESM.zip › Figure 2/Figure 2E/western Chk1.tif]

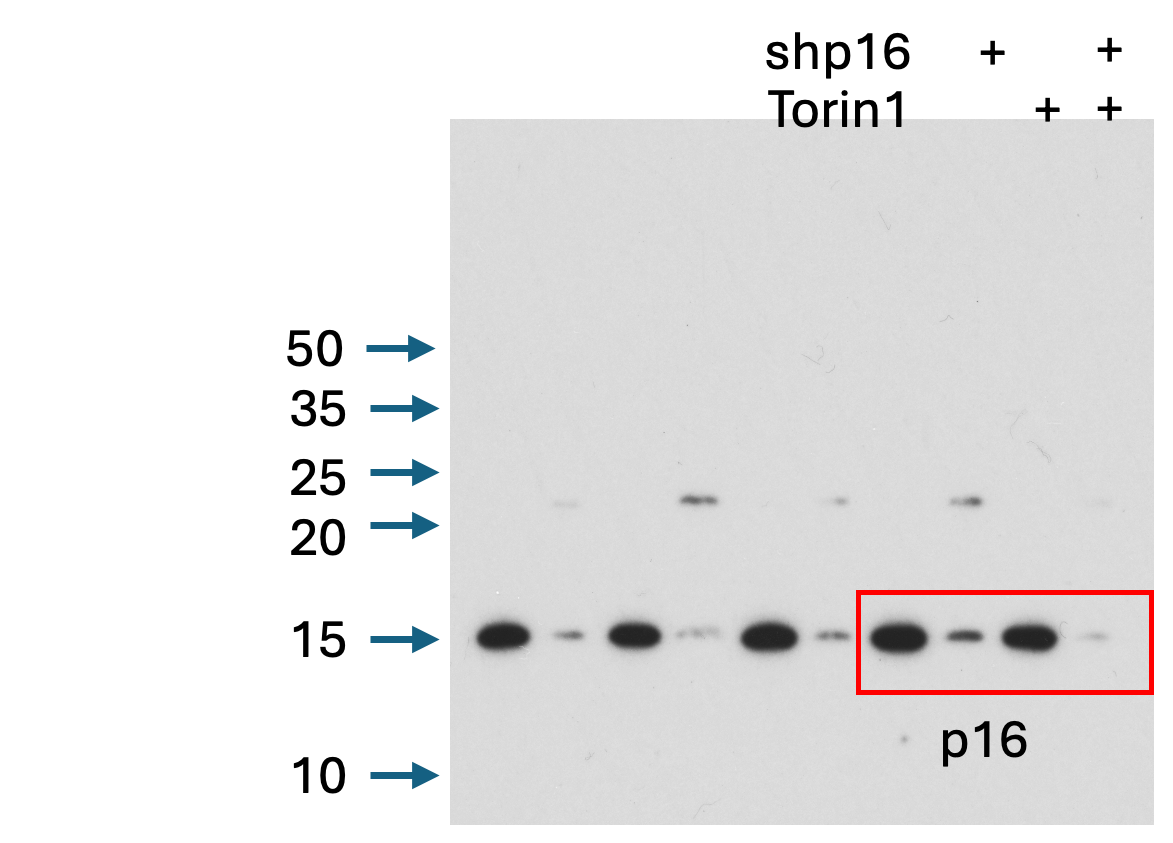

Supplement: Supplementary file 3 — Source data Fig. 2 [file 44319_2025_451_MOESM3_ESM.zip › Figure 2/Figure 2E/western p16.tif]

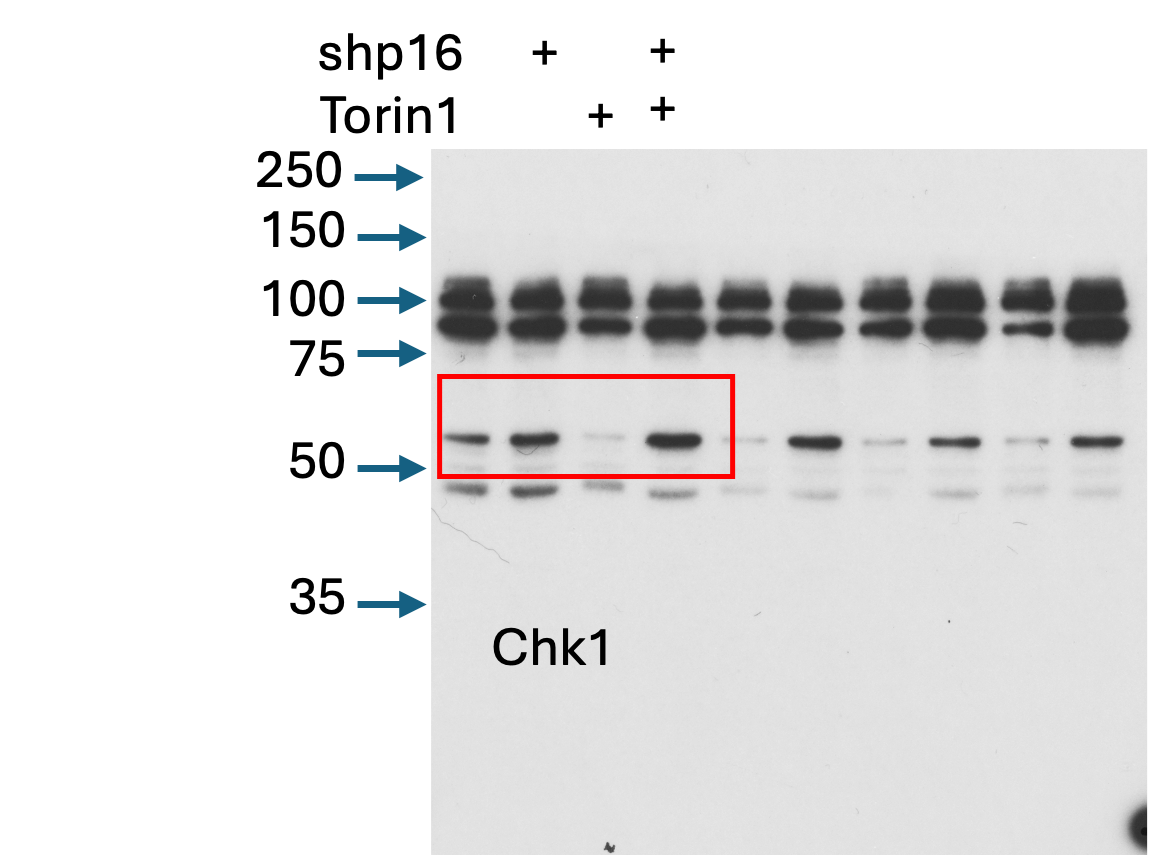

Supplement: Supplementary file 3 — Source data Fig. 2 [file 44319_2025_451_MOESM3_ESM.zip › Figure 2/Figure 2E/western pChk1.tif]

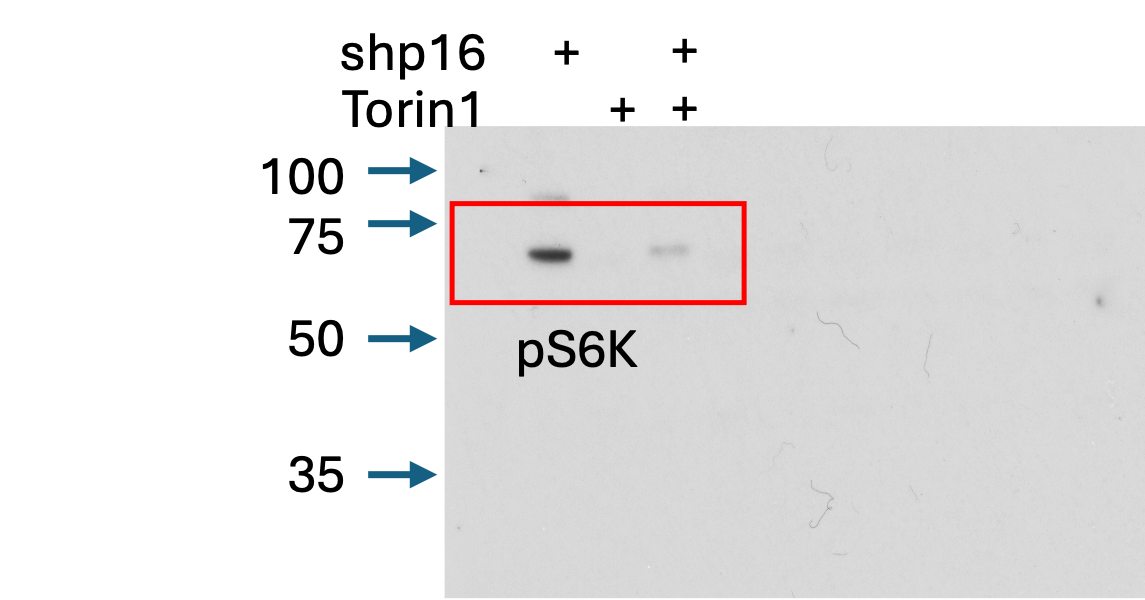

Supplement: Supplementary file 3 — Source data Fig. 2 [file 44319_2025_451_MOESM3_ESM.zip › Figure 2/Figure 2E/western pS6K.tif]

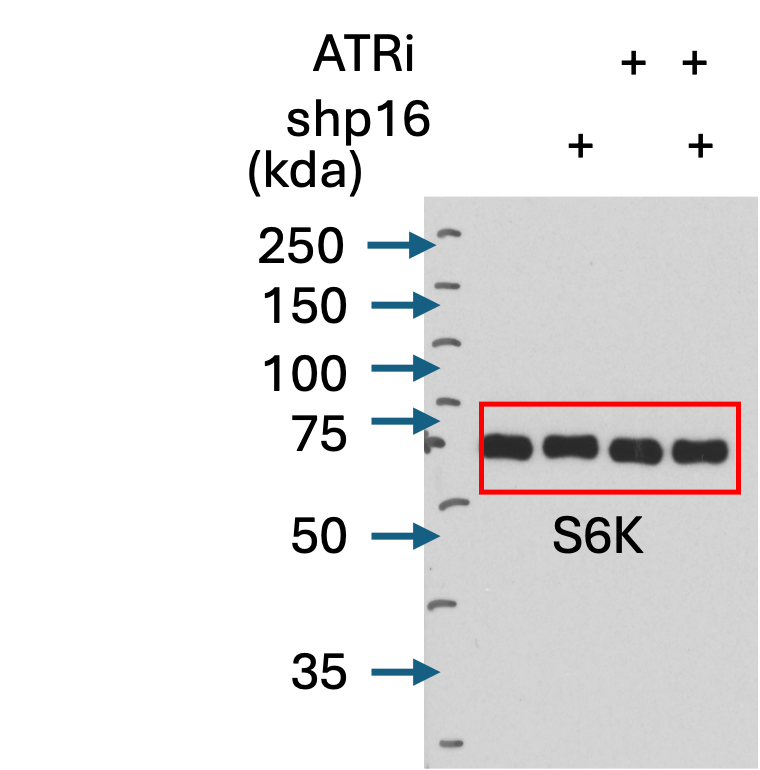

Supplement: Supplementary file 3 — Source data Fig. 2 [file 44319_2025_451_MOESM3_ESM.zip › Figure 2/Figure 2B/western S6K.tif]

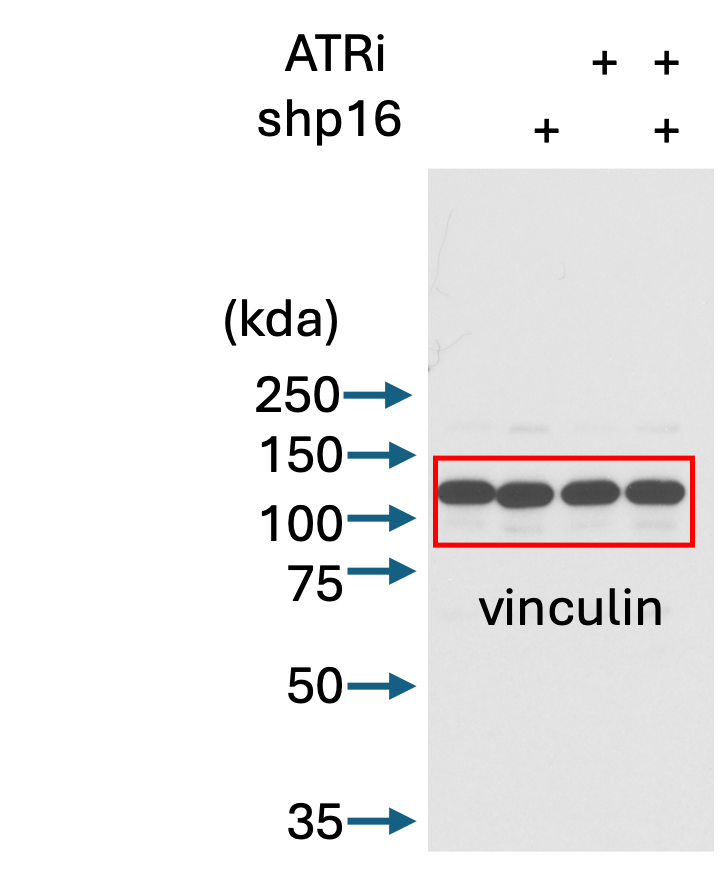

Supplement: Supplementary file 3 — Source data Fig. 2 [file 44319_2025_451_MOESM3_ESM.zip › Figure 2/Figure 2B/western vinculin.tif]

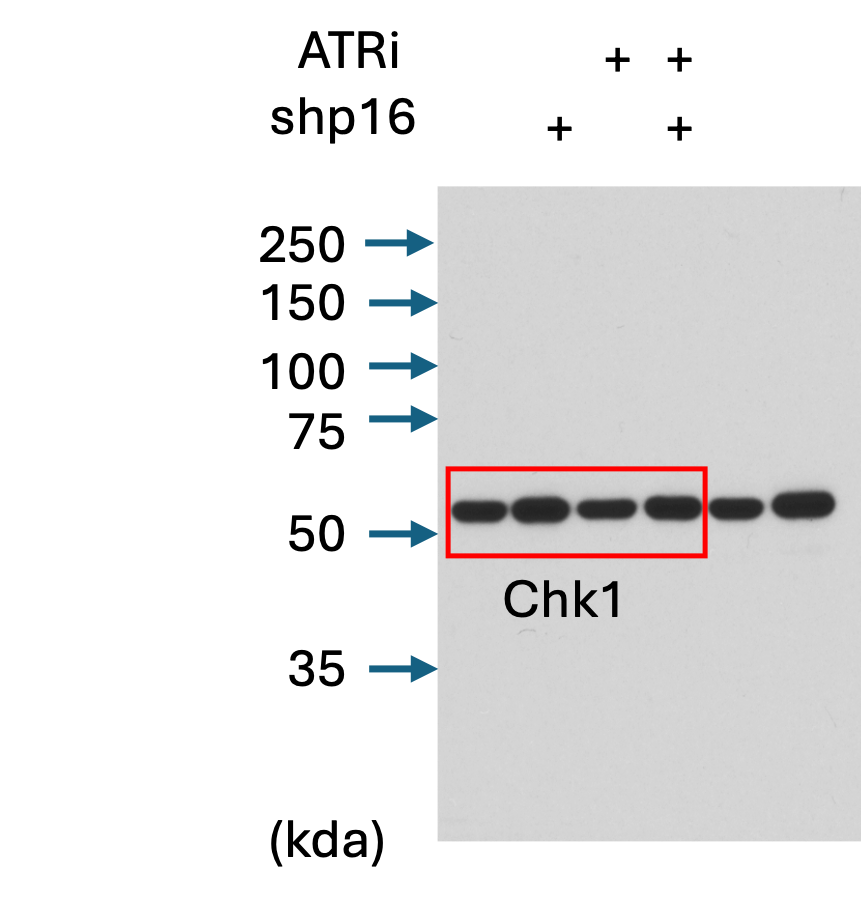

Supplement: Supplementary file 3 — Source data Fig. 2 [file 44319_2025_451_MOESM3_ESM.zip › Figure 2/Figure 2B/western Chk1.tif]

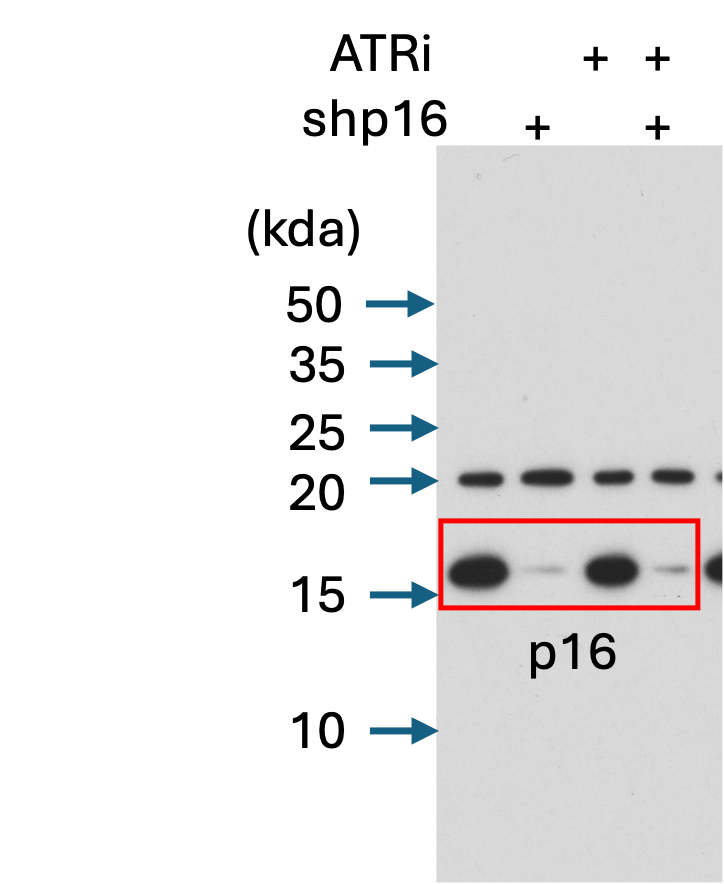

Supplement: Supplementary file 3 — Source data Fig. 2 [file 44319_2025_451_MOESM3_ESM.zip › Figure 2/Figure 2B/western p16.tif]

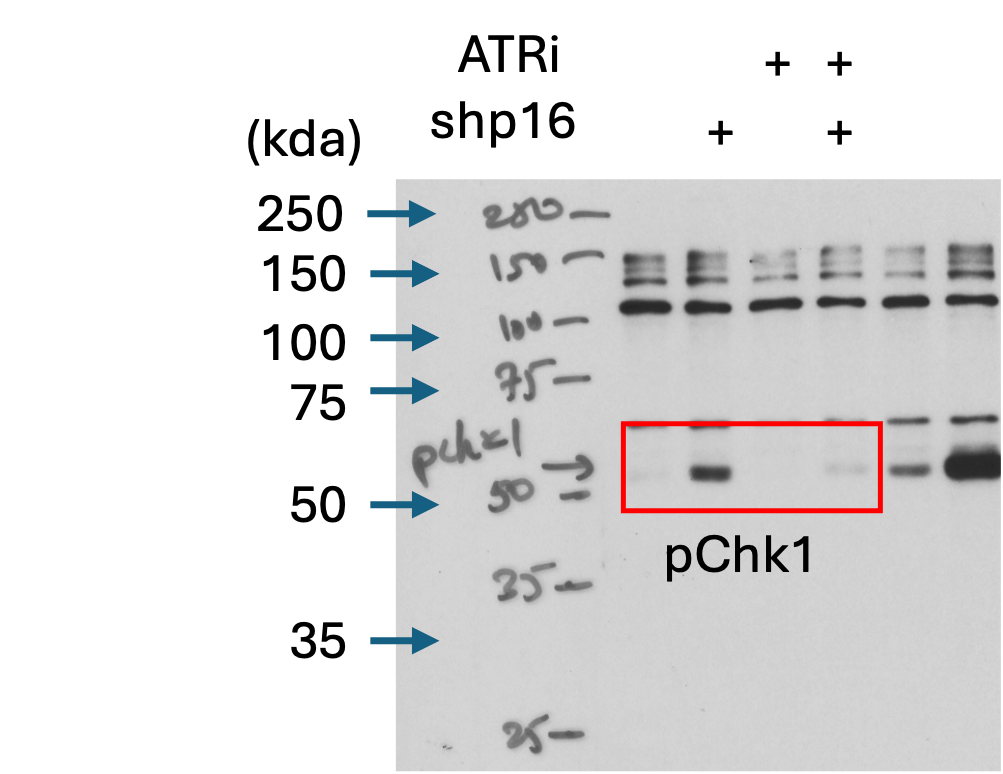

Supplement: Supplementary file 3 — Source data Fig. 2 [file 44319_2025_451_MOESM3_ESM.zip › Figure 2/Figure 2B/western pChk1.tif]

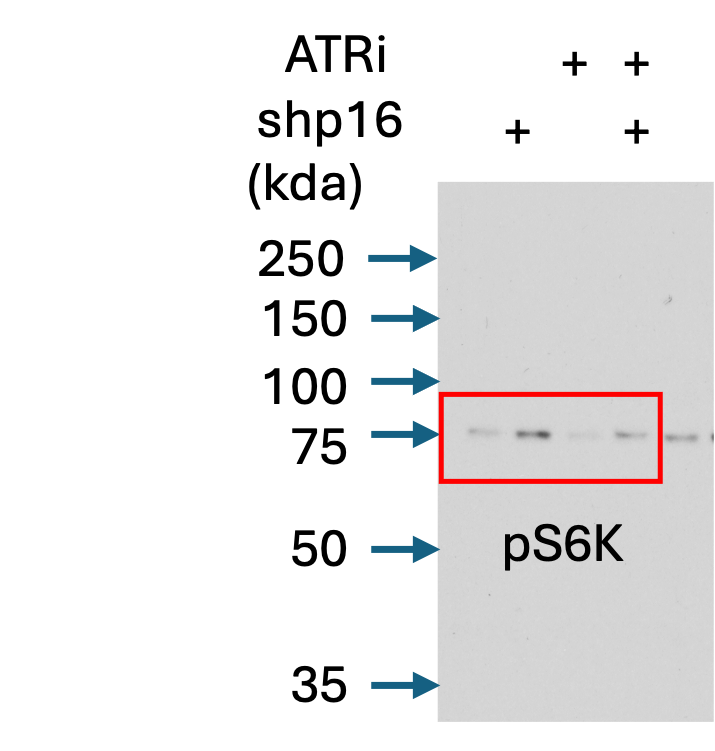

Supplement: Supplementary file 3 — Source data Fig. 2 [file 44319_2025_451_MOESM3_ESM.zip › Figure 2/Figure 2B/western pS6K.tif]

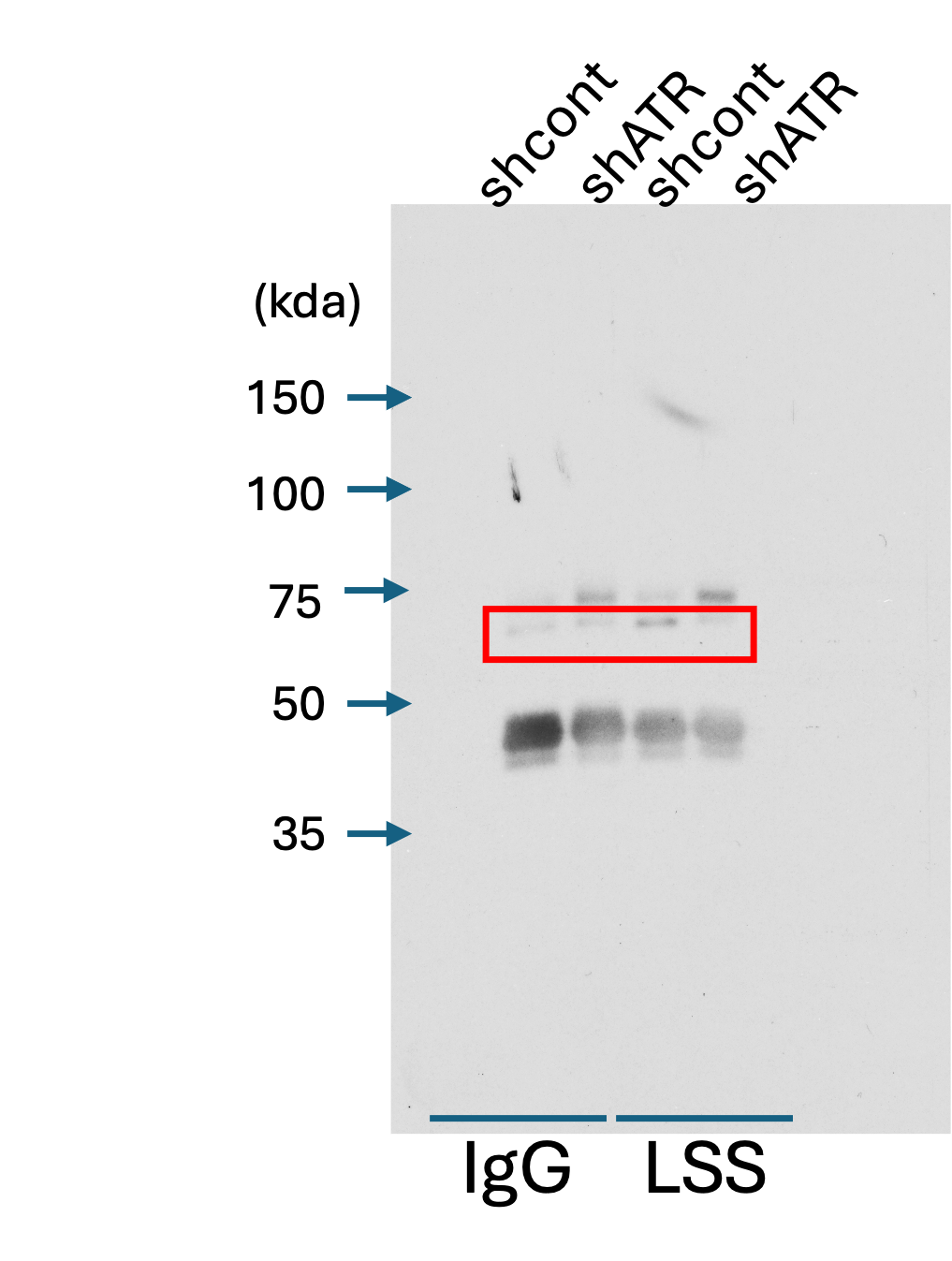

Supplement: Supplementary file 4 — Source data Fig. 3 [file 44319_2025_451_MOESM4_ESM.zip › Figure 3/Figure 3H/western ATM:ATR substrate.tif]

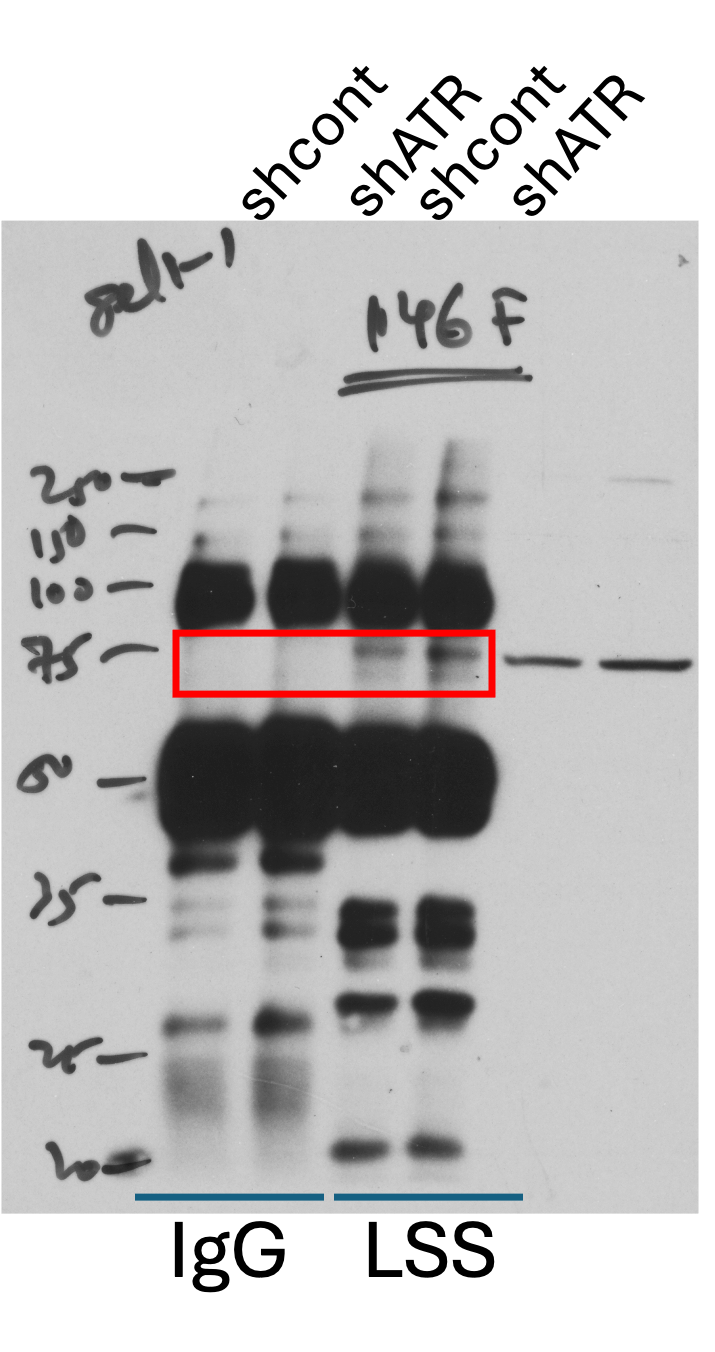

Supplement: Supplementary file 4 — Source data Fig. 3 [file 44319_2025_451_MOESM4_ESM.zip › Figure 3/Figure 3H/western LSS.tif]

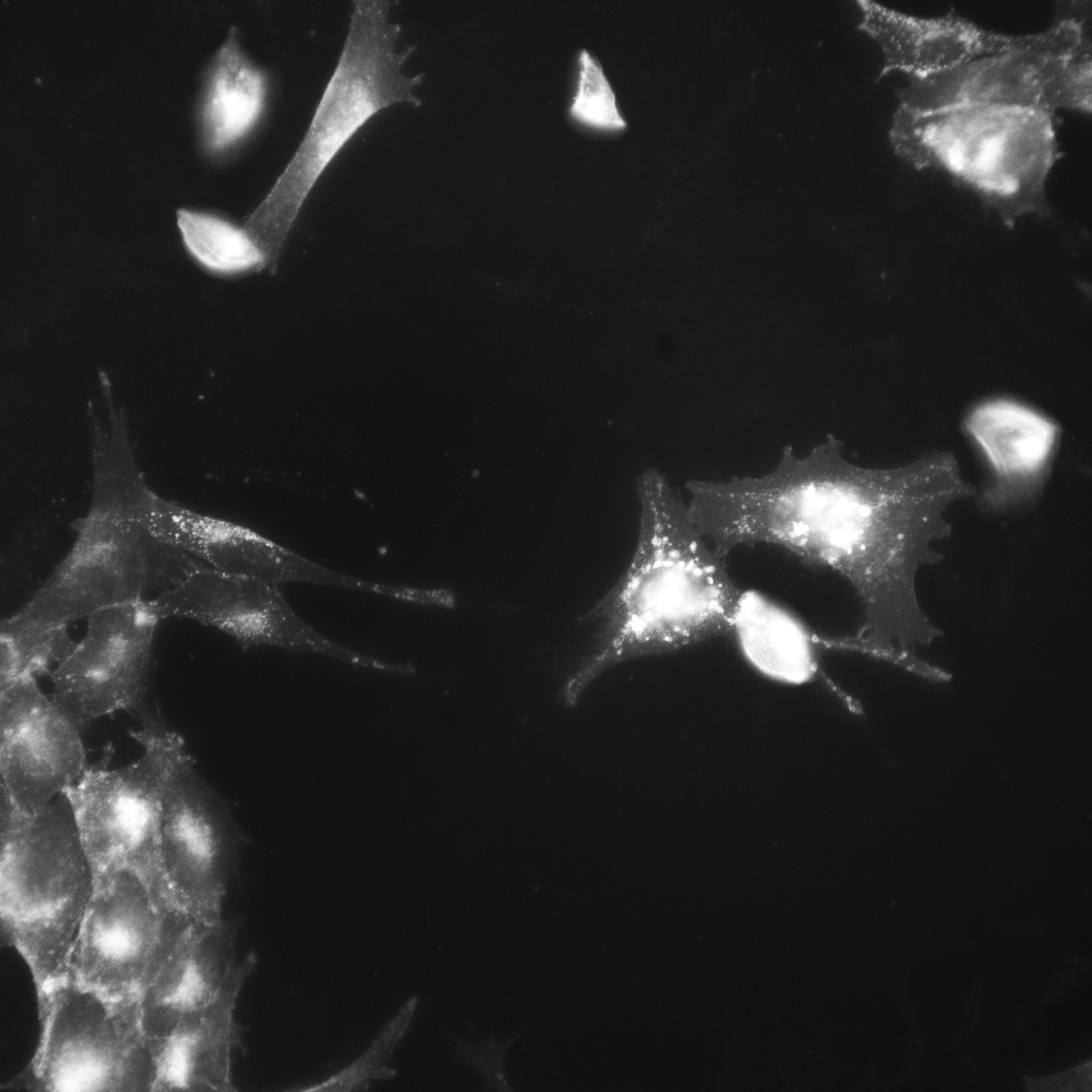

Supplement: Supplementary file 4 — Source data Fig. 3 [file 44319_2025_451_MOESM4_ESM.zip › Figure 3/Figure 3F/shp16.tif]

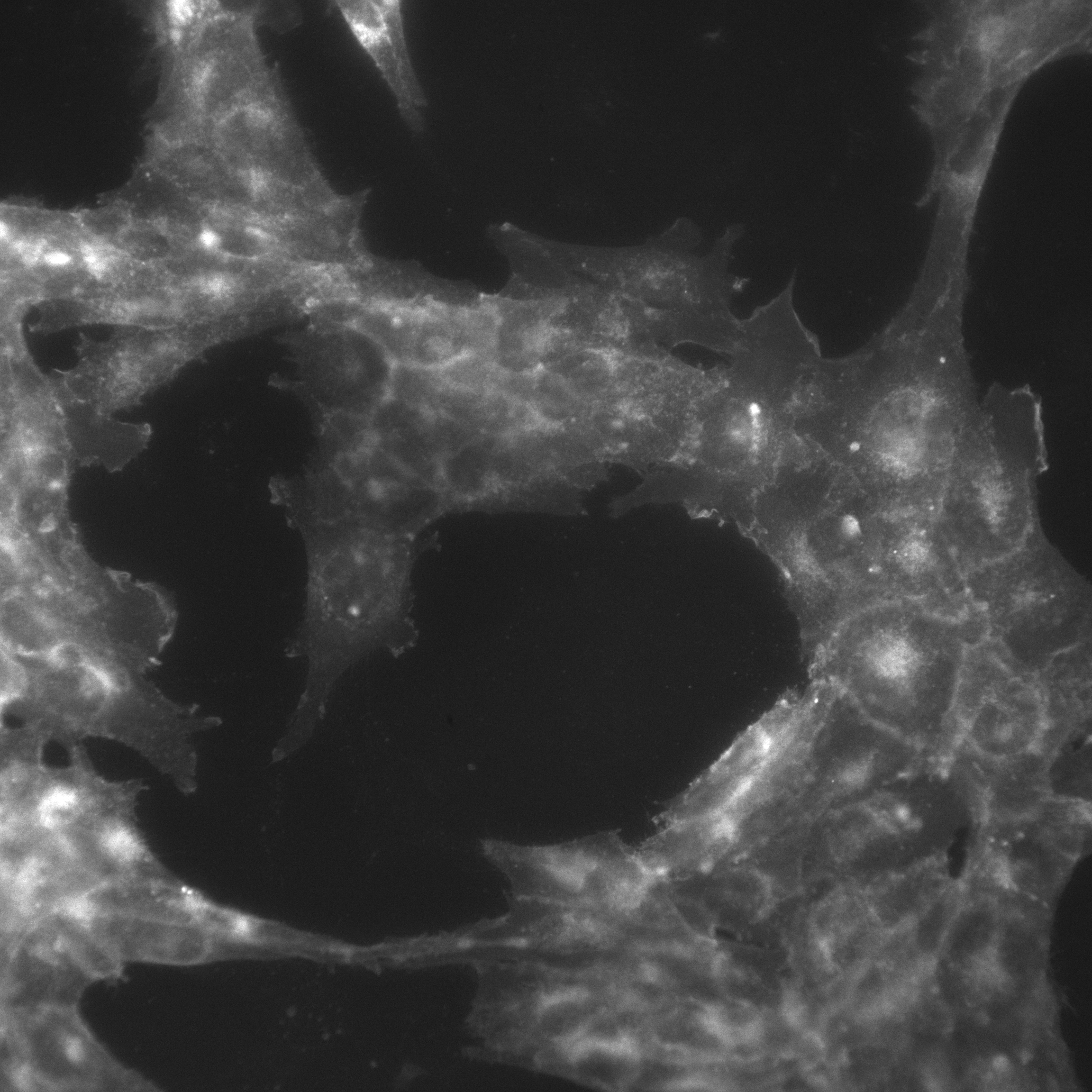

Supplement: Supplementary file 4 — Source data Fig. 3 [file 44319_2025_451_MOESM4_ESM.zip › Figure 3/Figure 3F/shp16-shATR#2.tif]

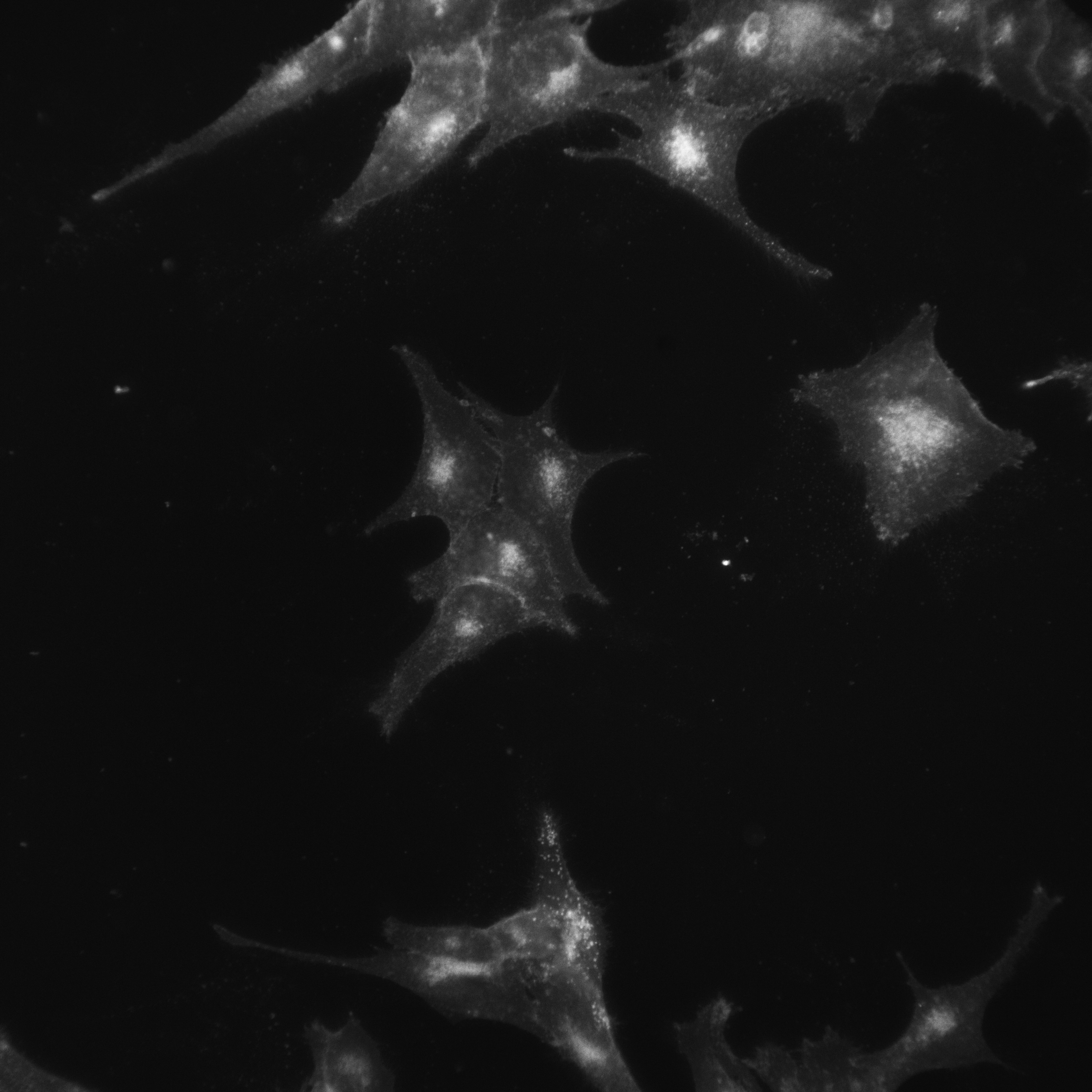

Supplement: Supplementary file 4 — Source data Fig. 3 [file 44319_2025_451_MOESM4_ESM.zip › Figure 3/Figure 3F/shp16-shATR#1.tif]

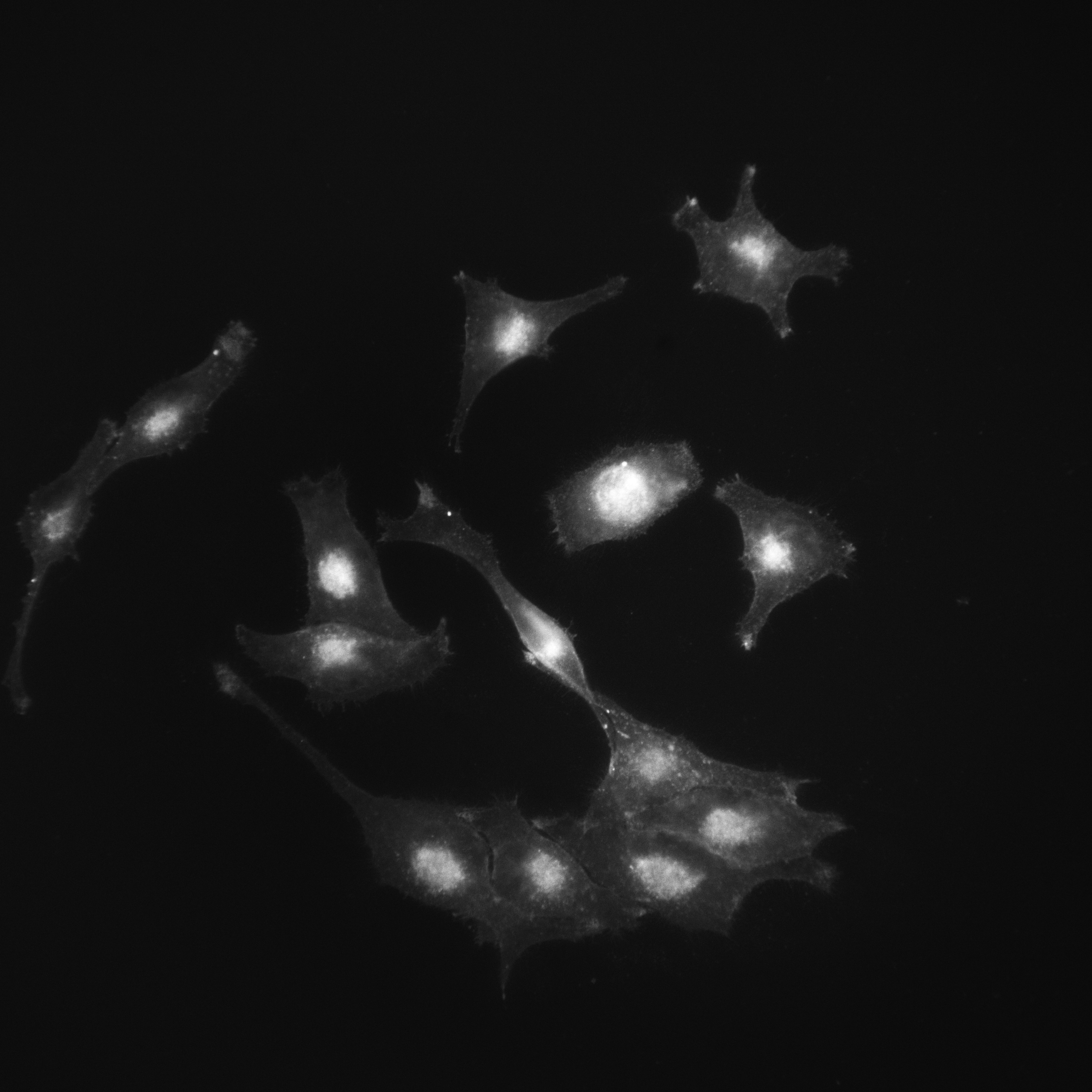

Supplement: Supplementary file 4 — Source data Fig. 3 [file 44319_2025_451_MOESM4_ESM.zip › Figure 3/Figure 3F/shctrl.tif]

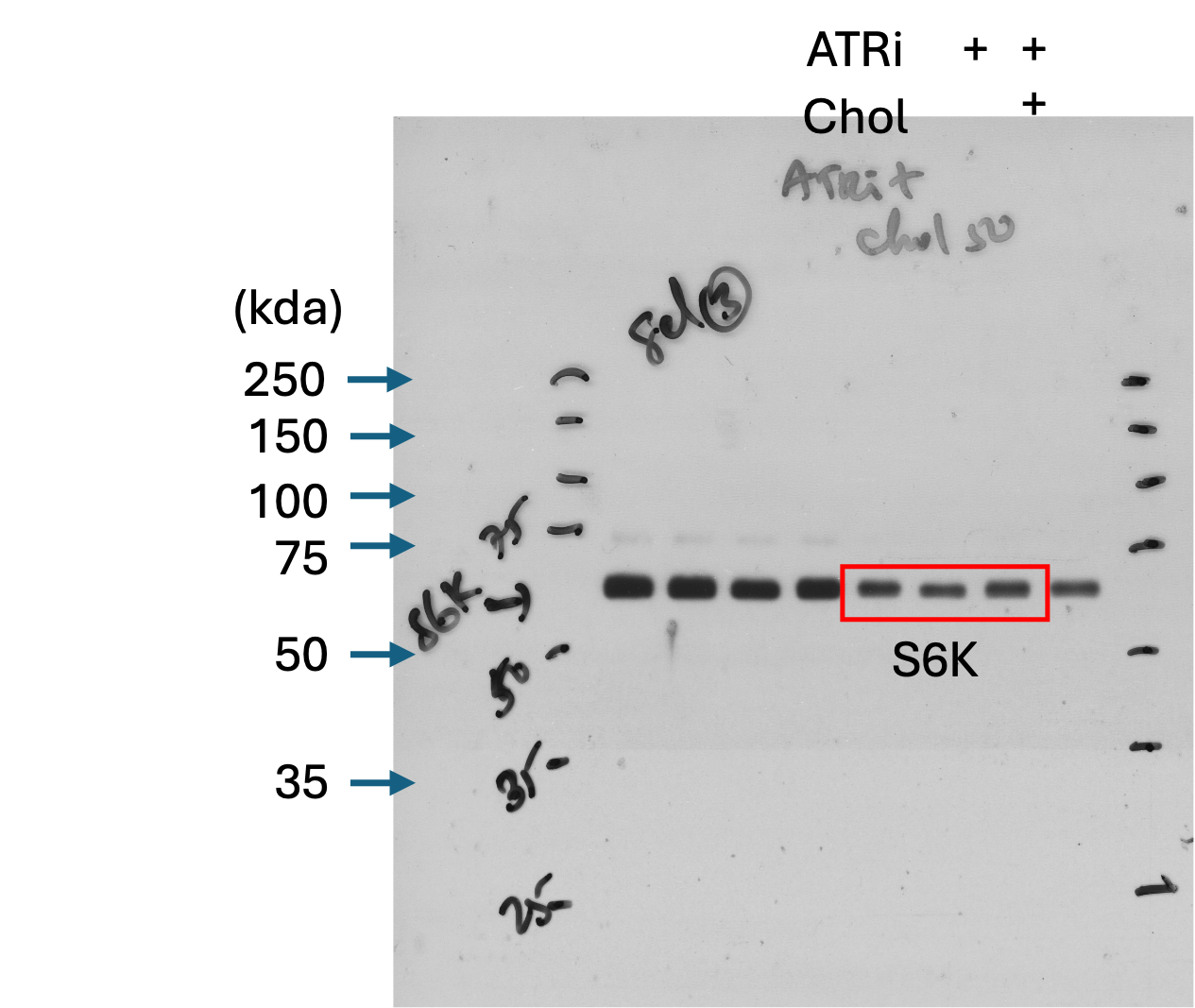

Supplement: Supplementary file 5 — Source data Fig. 4 [file 44319_2025_451_MOESM5_ESM.zip › Figure 4/Figure 4E/western S6K.tif]

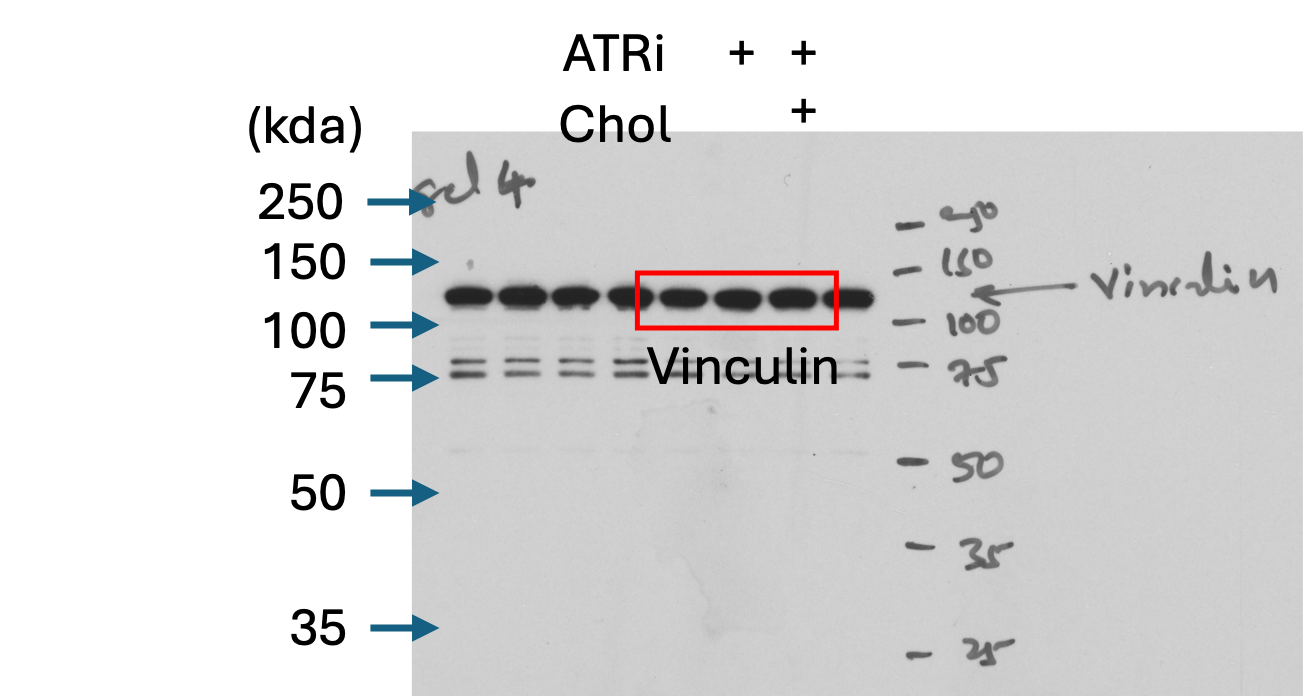

Supplement: Supplementary file 5 — Source data Fig. 4 [file 44319_2025_451_MOESM5_ESM.zip › Figure 4/Figure 4E/western vinculin.tif]

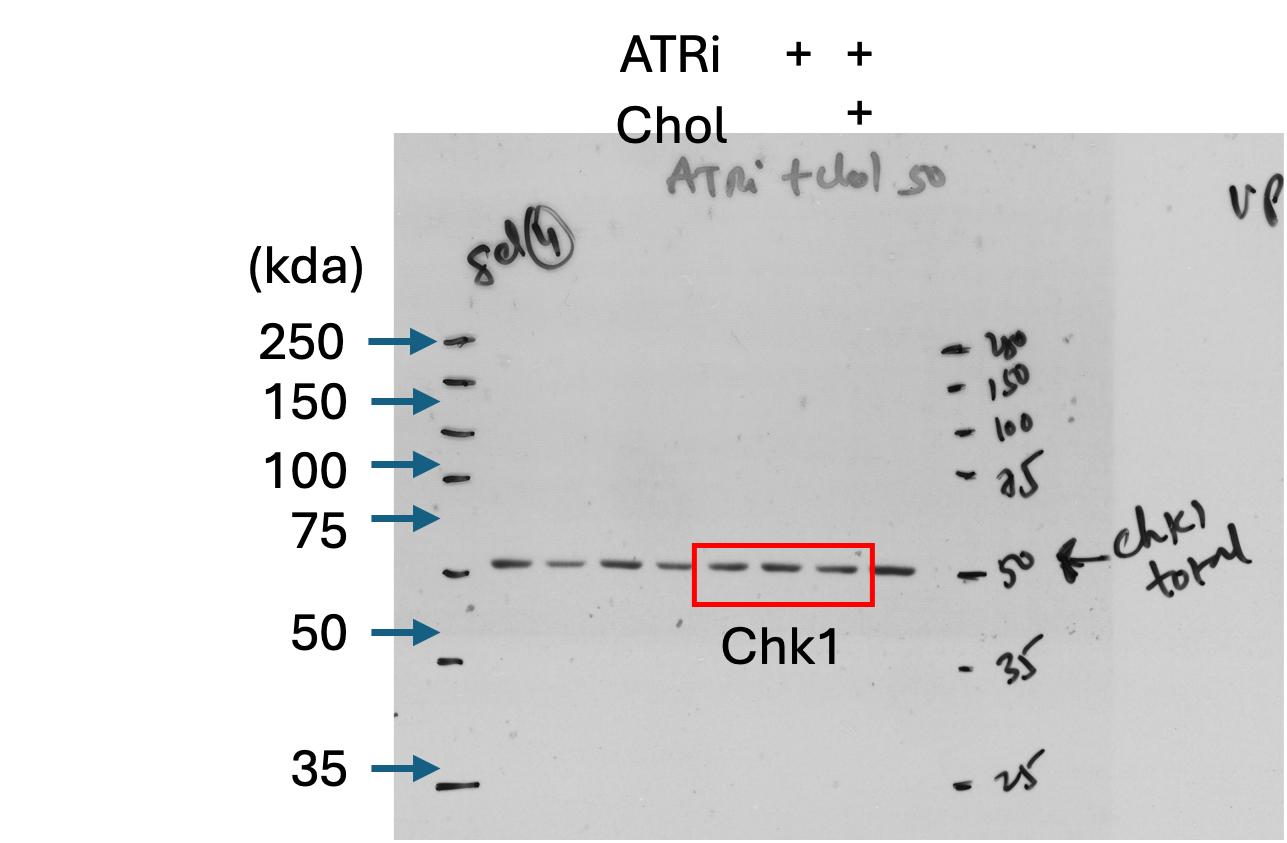

Supplement: Supplementary file 5 — Source data Fig. 4 [file 44319_2025_451_MOESM5_ESM.zip › Figure 4/Figure 4E/western Chk1.tif]

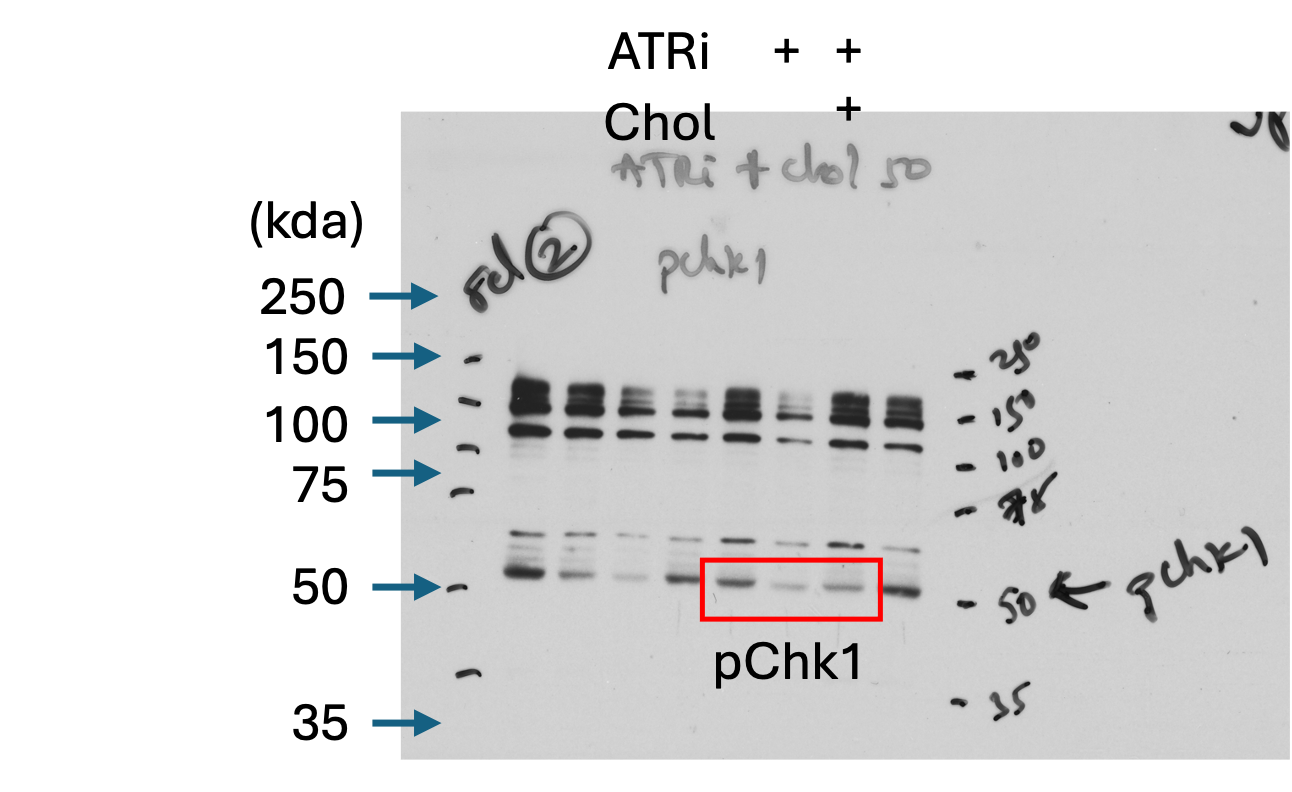

Supplement: Supplementary file 5 — Source data Fig. 4 [file 44319_2025_451_MOESM5_ESM.zip › Figure 4/Figure 4E/western pChk1.tif]

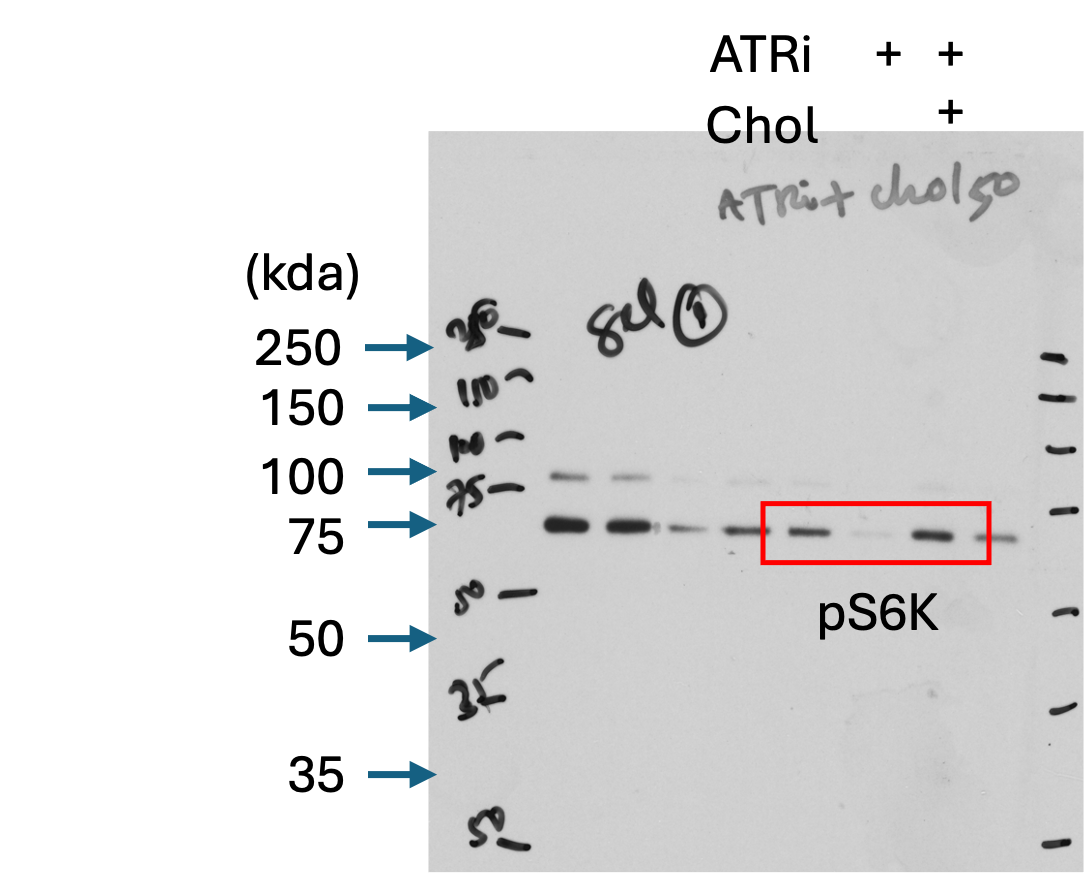

Supplement: Supplementary file 5 — Source data Fig. 4 [file 44319_2025_451_MOESM5_ESM.zip › Figure 4/Figure 4E/western pS6K.tif]

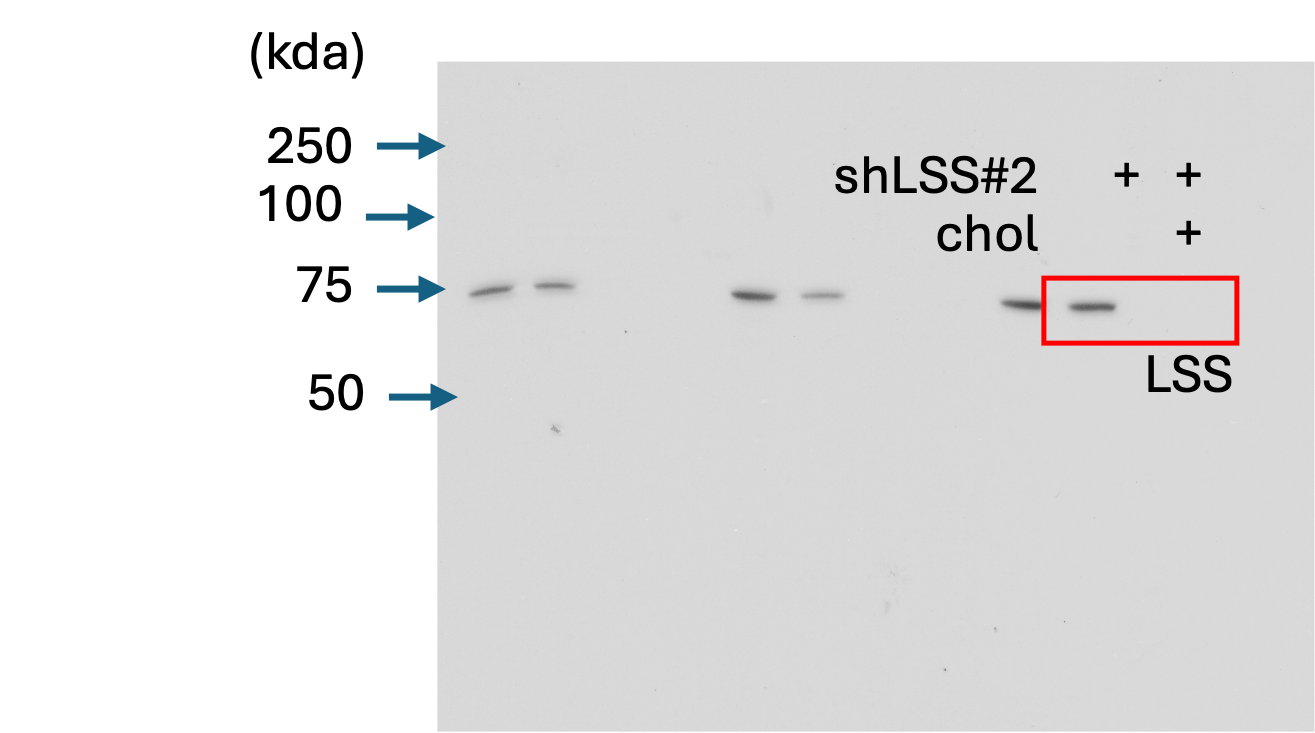

Supplement: Supplementary file 5 — Source data Fig. 4 [file 44319_2025_451_MOESM5_ESM.zip › Figure 4/Figure 4B/western LSS shLSS#2 RPMI7951.tif]

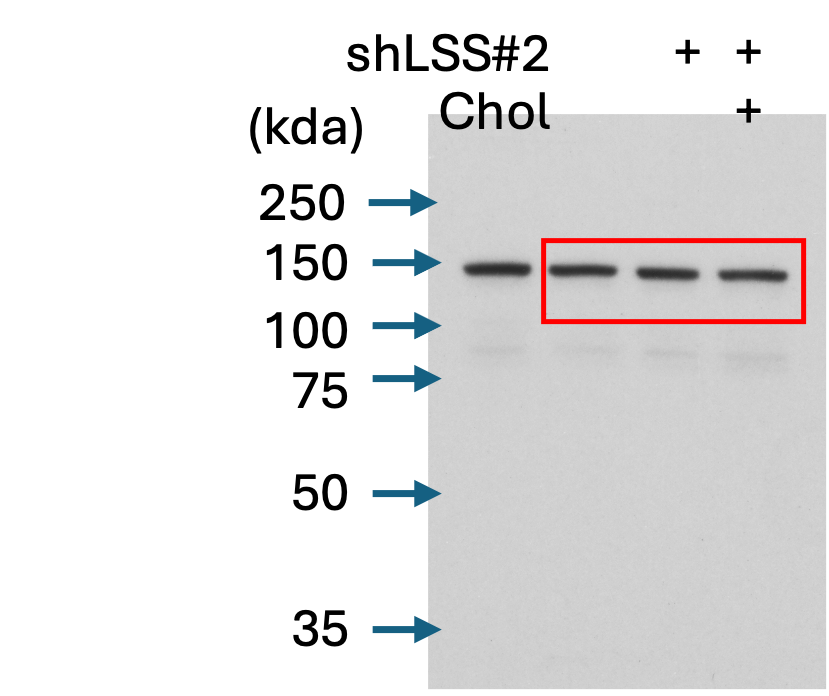

Supplement: Supplementary file 5 — Source data Fig. 4 [file 44319_2025_451_MOESM5_ESM.zip › Figure 4/Figure 4B/western vinculin shLSS#2 SKMel28.tif]

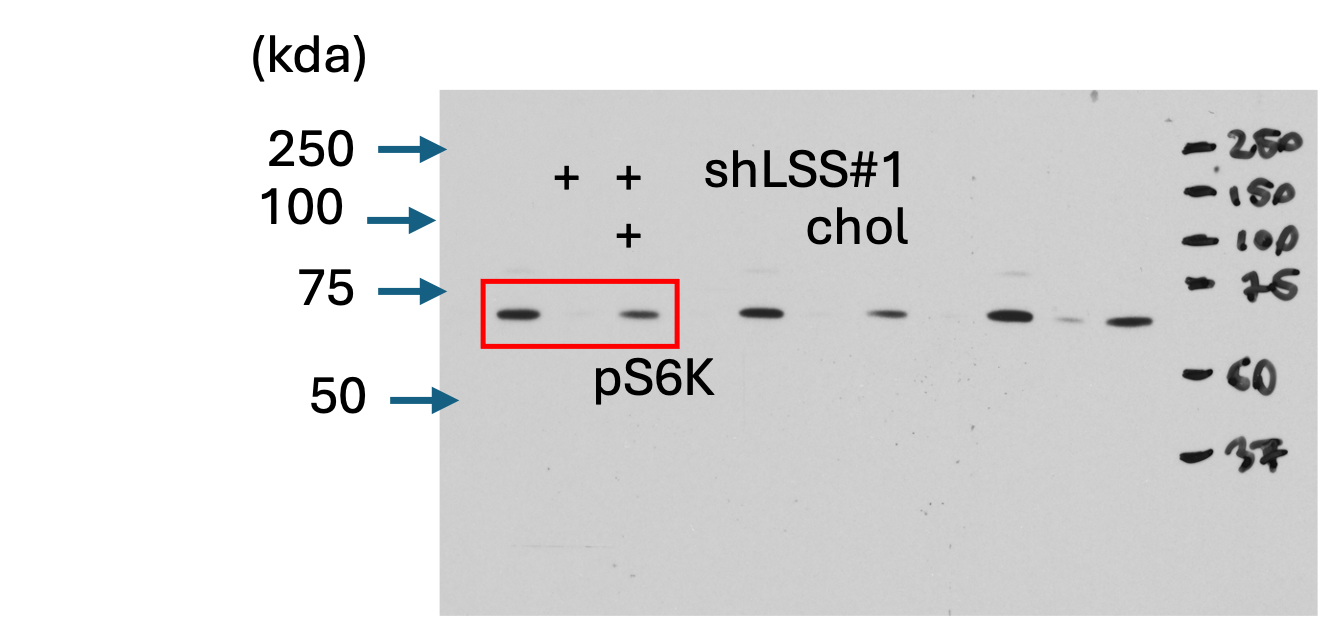

Supplement: Supplementary file 5 — Source data Fig. 4 [file 44319_2025_451_MOESM5_ESM.zip › Figure 4/Figure 4B/western pS6K shLSS#1 RPMI7951.tif]

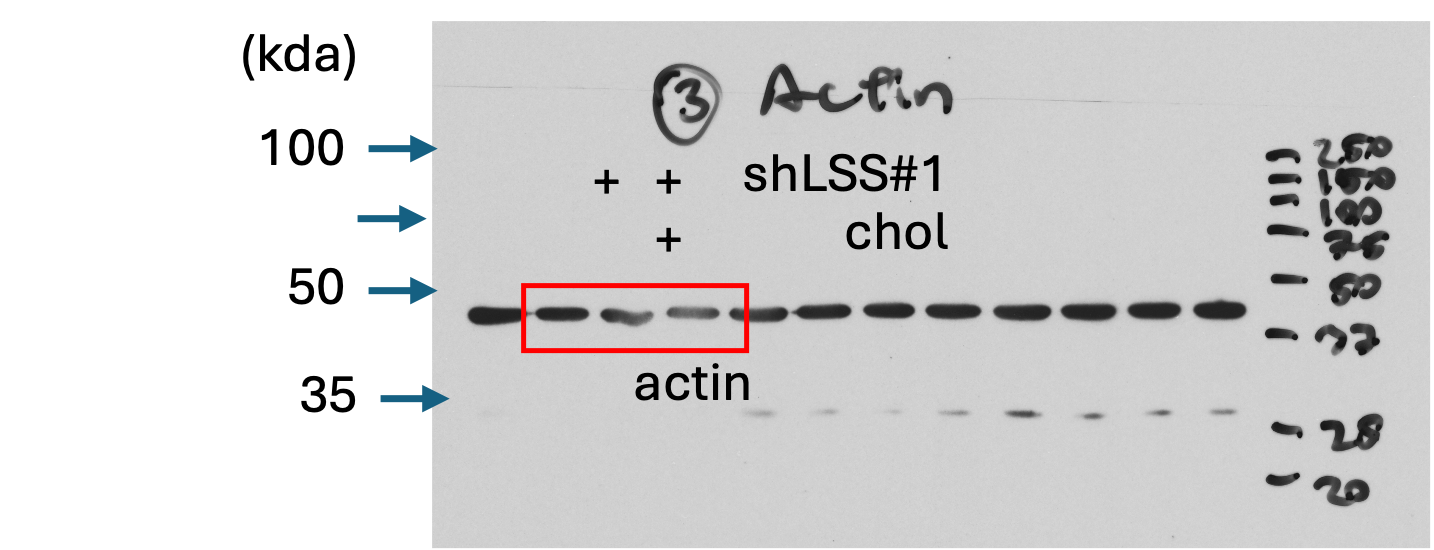

Supplement: Supplementary file 5 — Source data Fig. 4 [file 44319_2025_451_MOESM5_ESM.zip › Figure 4/Figure 4B/western actin shLSS#1 RPMI7951.tif]

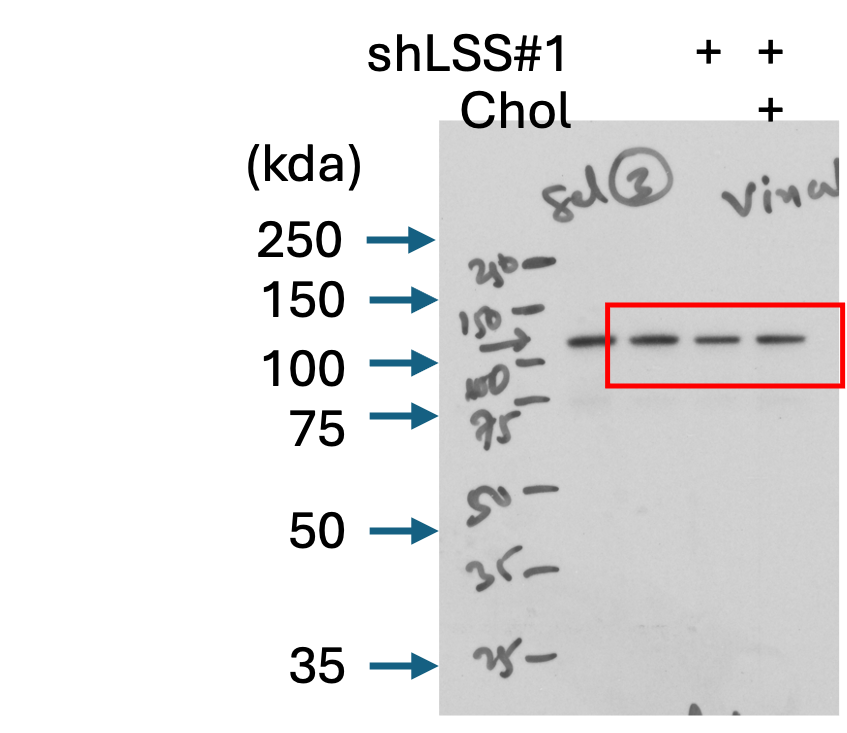

Supplement: Supplementary file 5 — Source data Fig. 4 [file 44319_2025_451_MOESM5_ESM.zip › Figure 4/Figure 4B/western vinculin shLSS#1 SKMel28.tif]

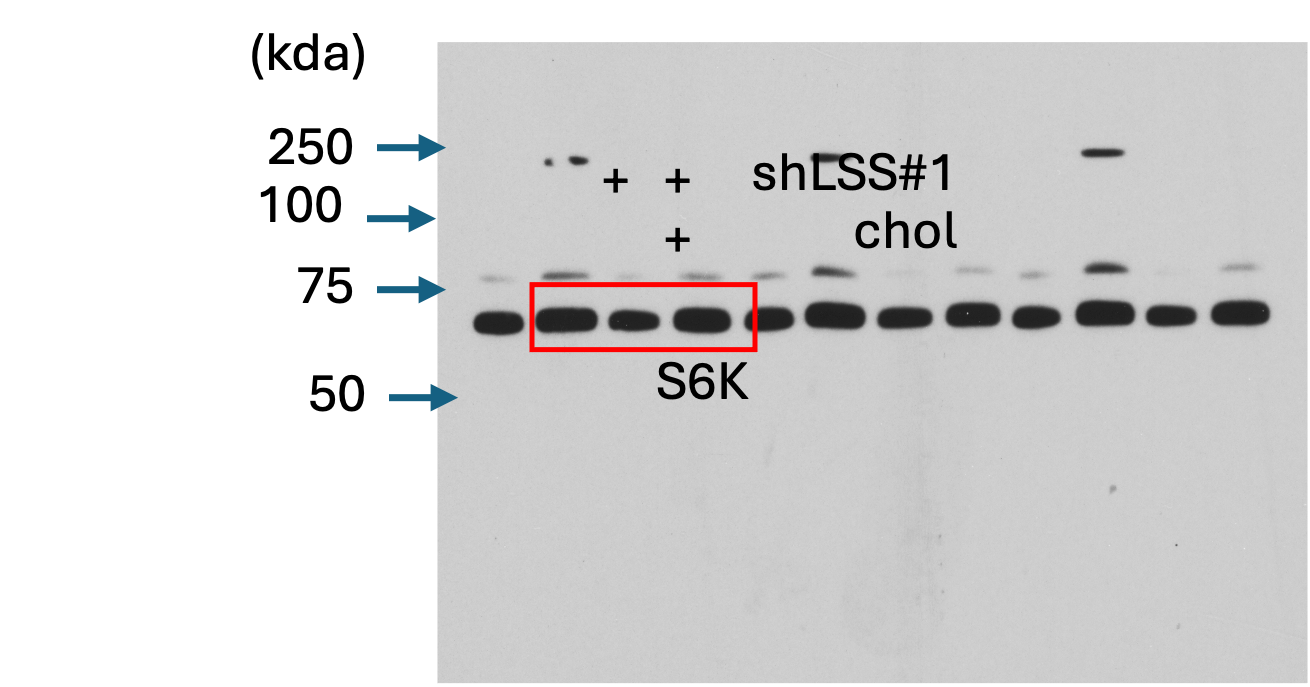

Supplement: Supplementary file 5 — Source data Fig. 4 [file 44319_2025_451_MOESM5_ESM.zip › Figure 4/Figure 4B/western S6K shLSS#1 RPMI7951.tif]

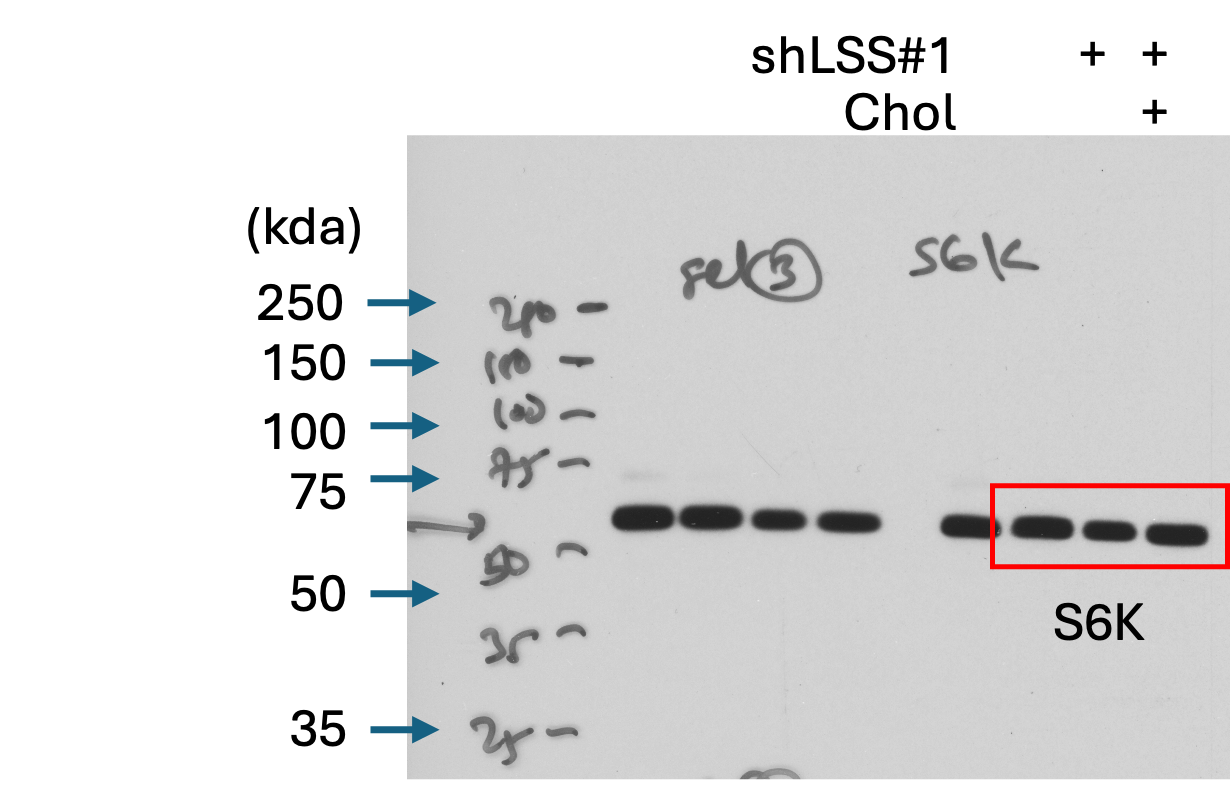

Supplement: Supplementary file 5 — Source data Fig. 4 [file 44319_2025_451_MOESM5_ESM.zip › Figure 4/Figure 4B/western S6K shLSS#1 SKMel28.tif]

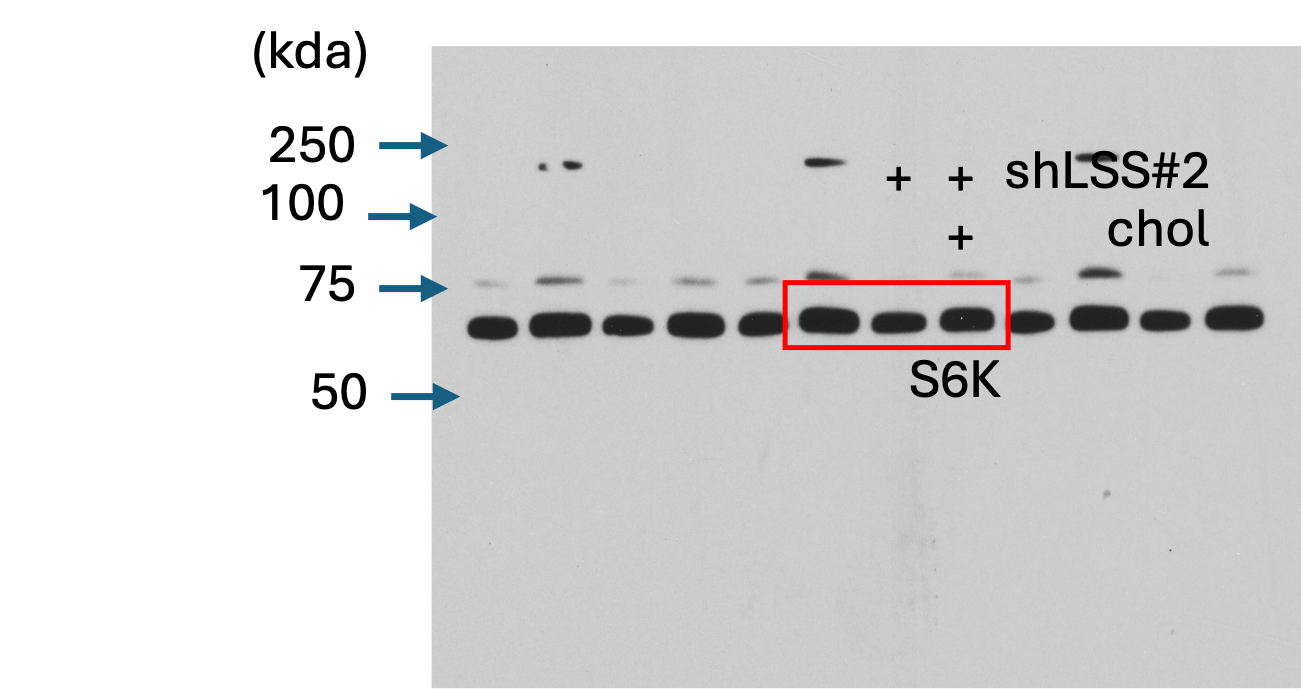

Supplement: Supplementary file 5 — Source data Fig. 4 [file 44319_2025_451_MOESM5_ESM.zip › Figure 4/Figure 4B/western S6K shLSS#2 RPMI7951.tif]

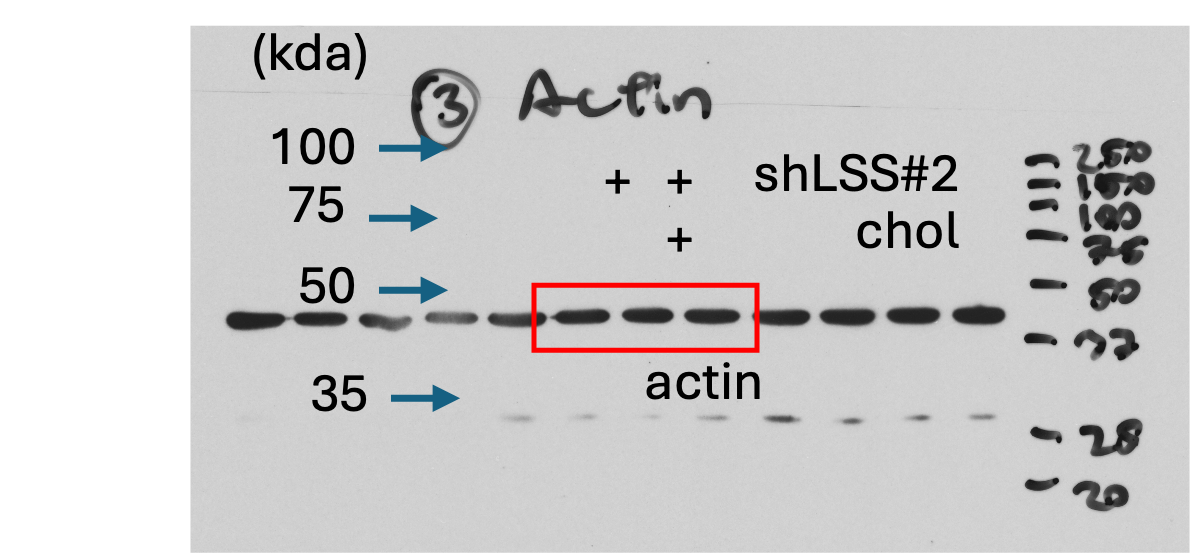

Supplement: Supplementary file 5 — Source data Fig. 4 [file 44319_2025_451_MOESM5_ESM.zip › Figure 4/Figure 4B/western actin shLSS#2 RPMI7951.tif]

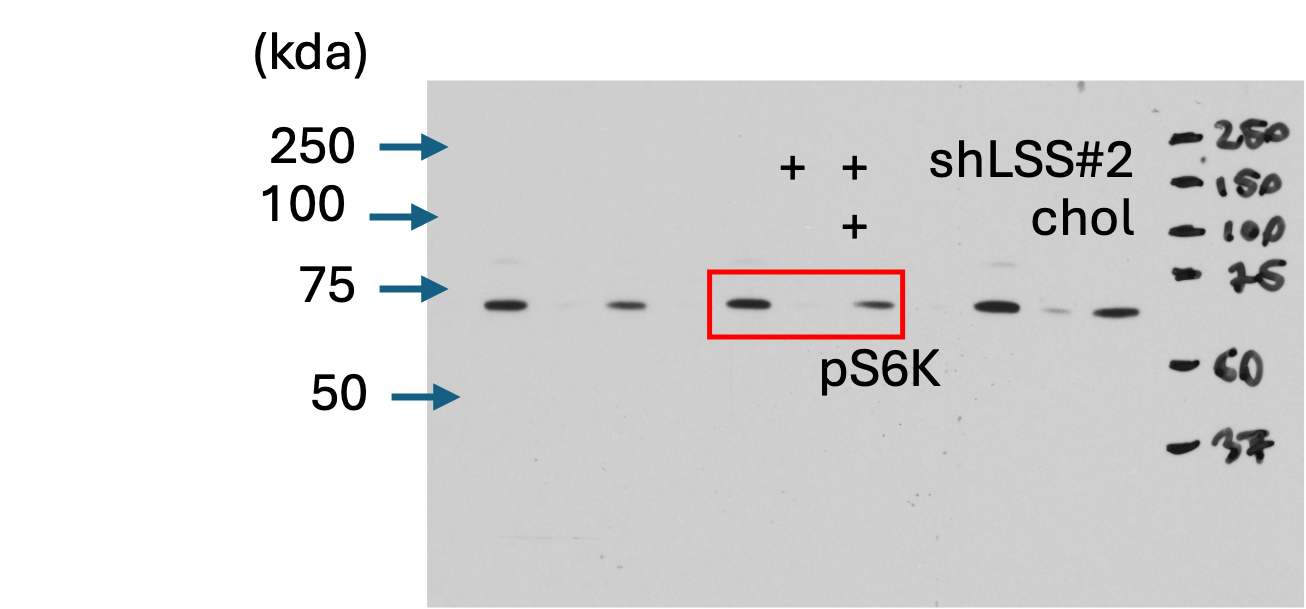

Supplement: Supplementary file 5 — Source data Fig. 4 [file 44319_2025_451_MOESM5_ESM.zip › Figure 4/Figure 4B/western pS6K shLSS#2 RPMI7951.tif]

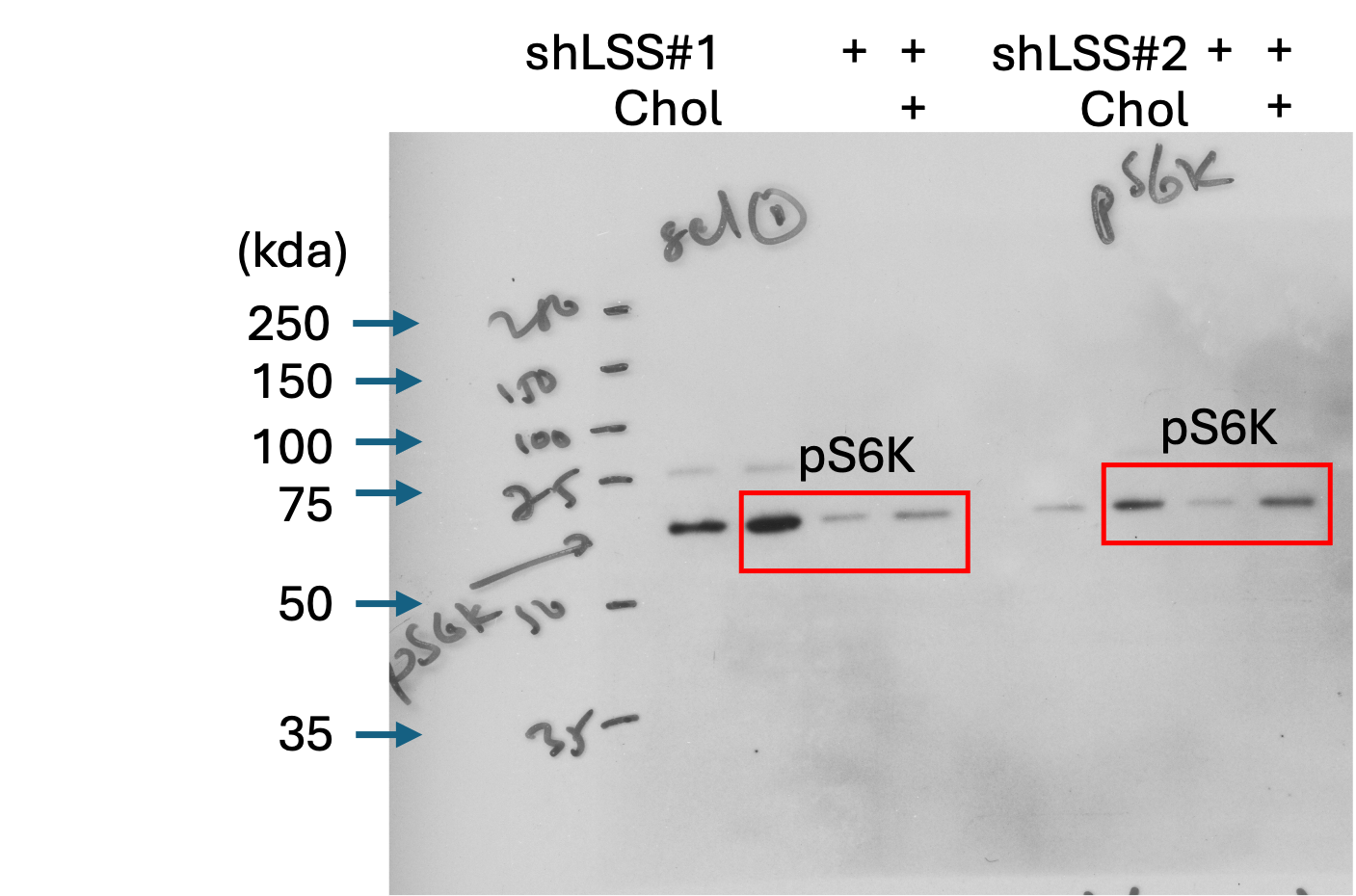

Supplement: Supplementary file 5 — Source data Fig. 4 [file 44319_2025_451_MOESM5_ESM.zip › Figure 4/Figure 4B/western pS6K SKMel28.tif]

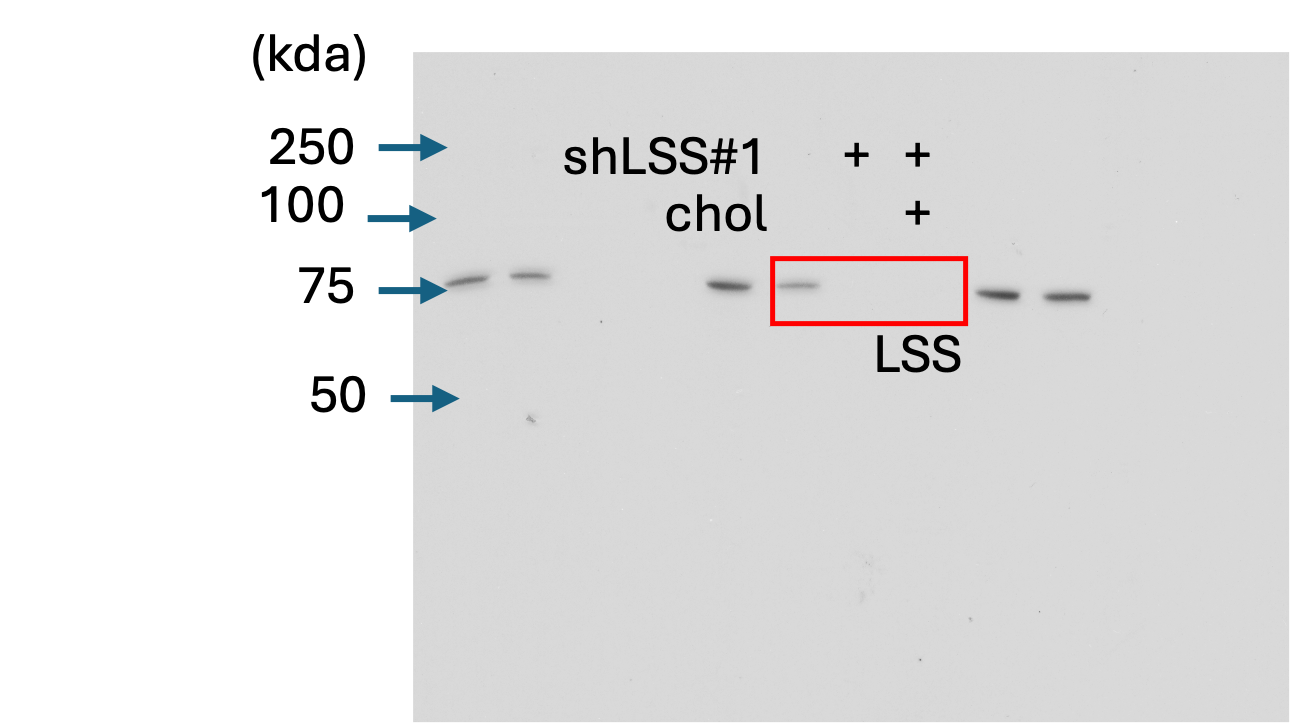

Supplement: Supplementary file 5 — Source data Fig. 4 [file 44319_2025_451_MOESM5_ESM.zip › Figure 4/Figure 4B/western LSS shLSS#1 RPMI7951.tif]

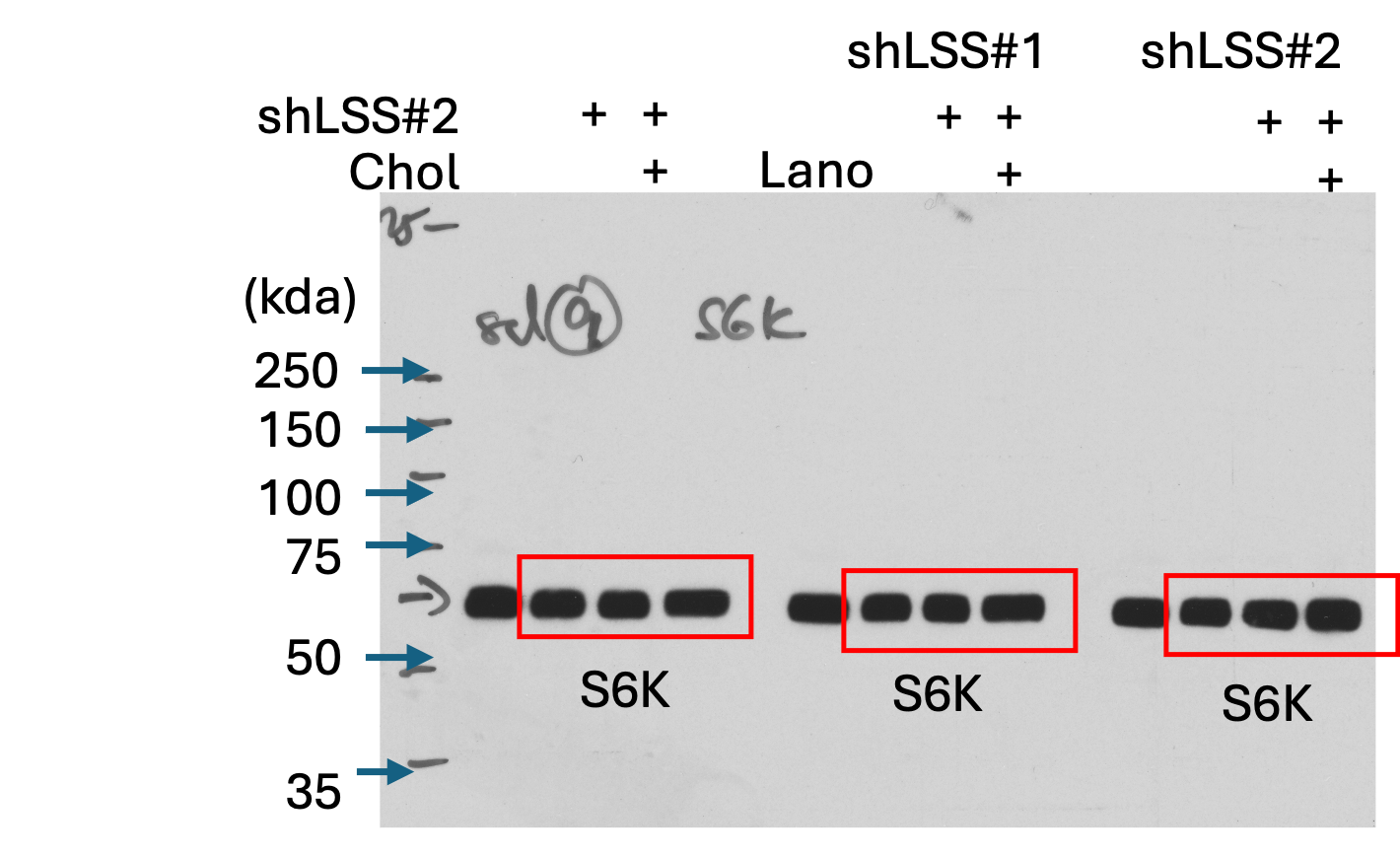

Supplement: Supplementary file 5 — Source data Fig. 4 [file 44319_2025_451_MOESM5_ESM.zip › Figure 4/Figure 4B/western S6K shLSS#2 SKMel28.tif]

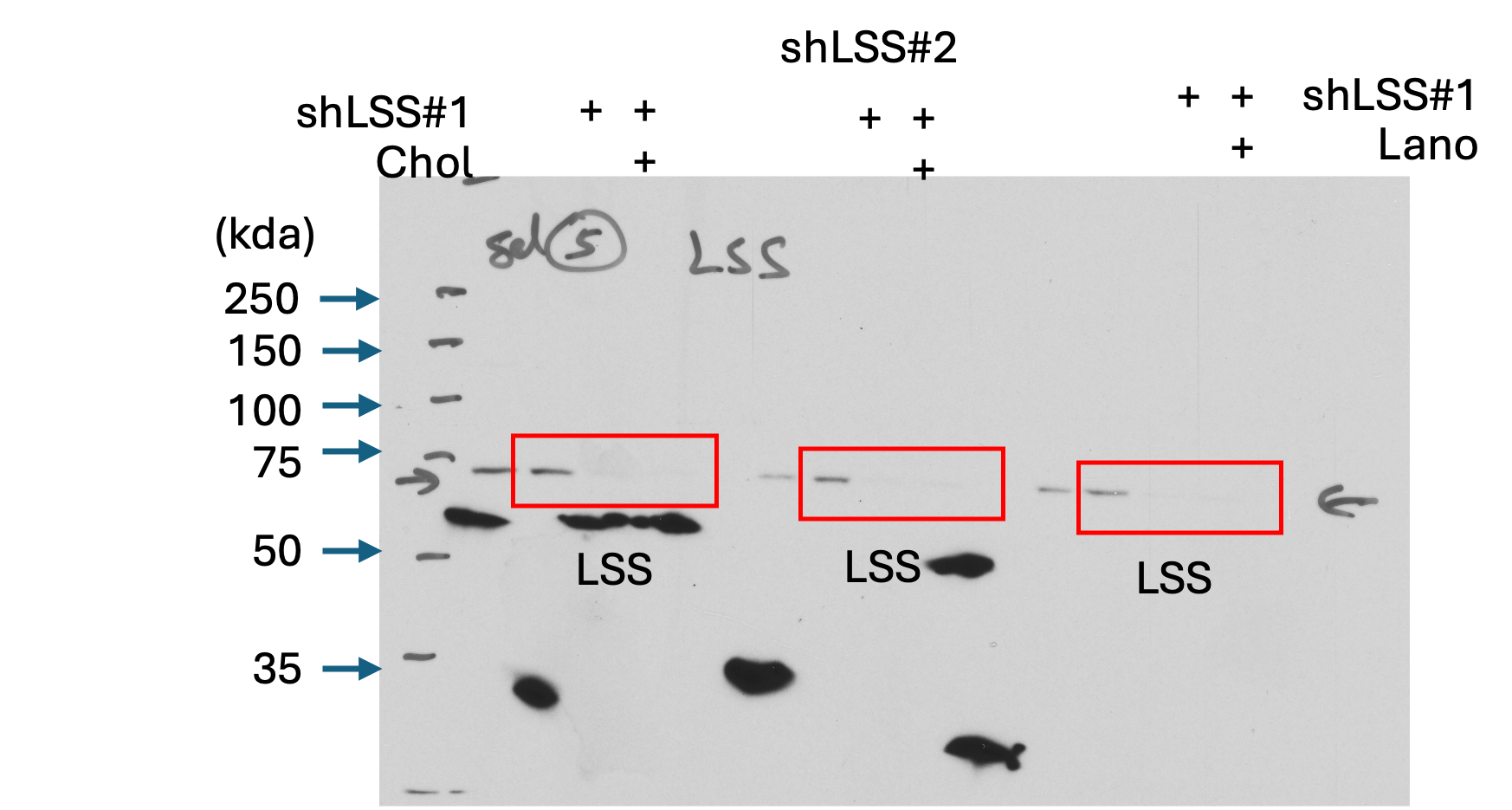

Supplement: Supplementary file 5 — Source data Fig. 4 [file 44319_2025_451_MOESM5_ESM.zip › Figure 4/Figure 4B/western LSS SKMel28.tif]

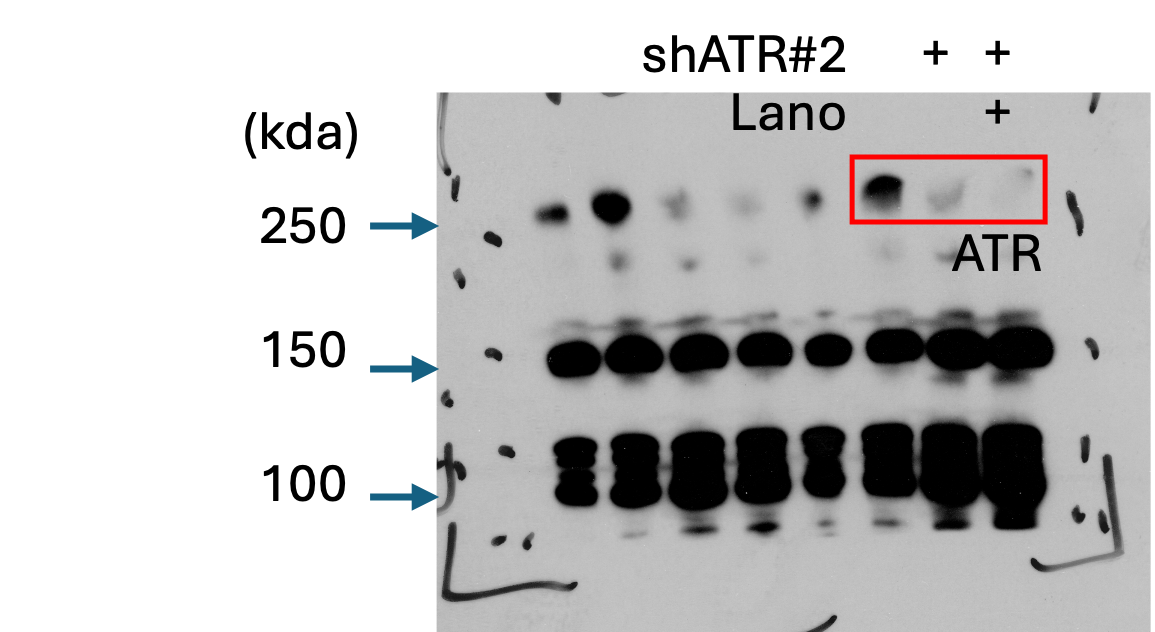

Supplement: Supplementary file 5 — Source data Fig. 4 [file 44319_2025_451_MOESM5_ESM.zip › Figure 4/Figure 4C/western ATR shATR#2 RPMI7951.tif]

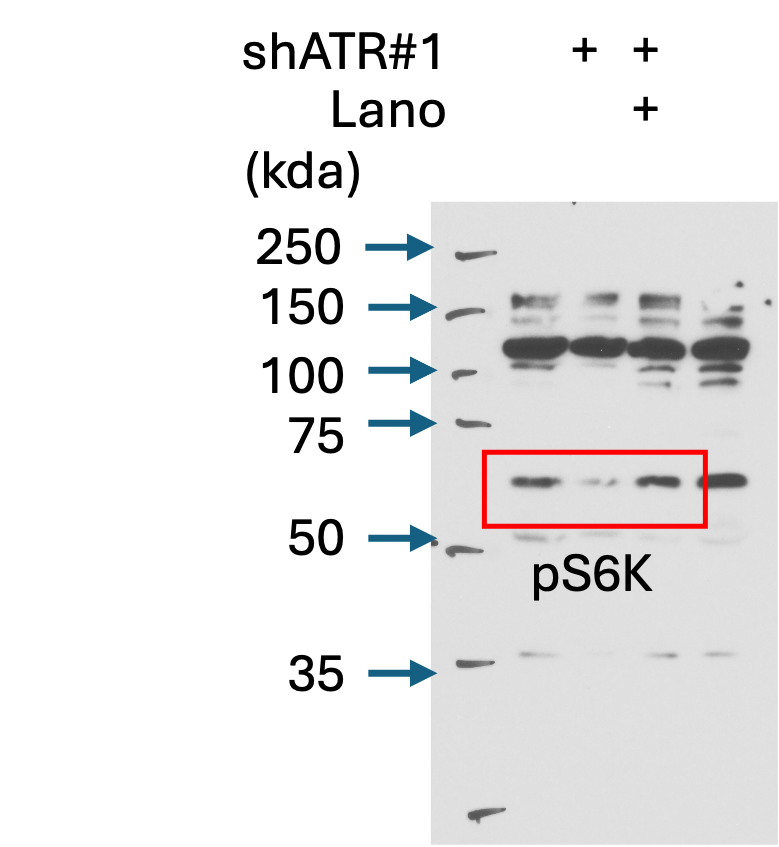

Supplement: Supplementary file 5 — Source data Fig. 4 [file 44319_2025_451_MOESM5_ESM.zip › Figure 4/Figure 4C/western pS6K shATR#1 SKMel28.tif]

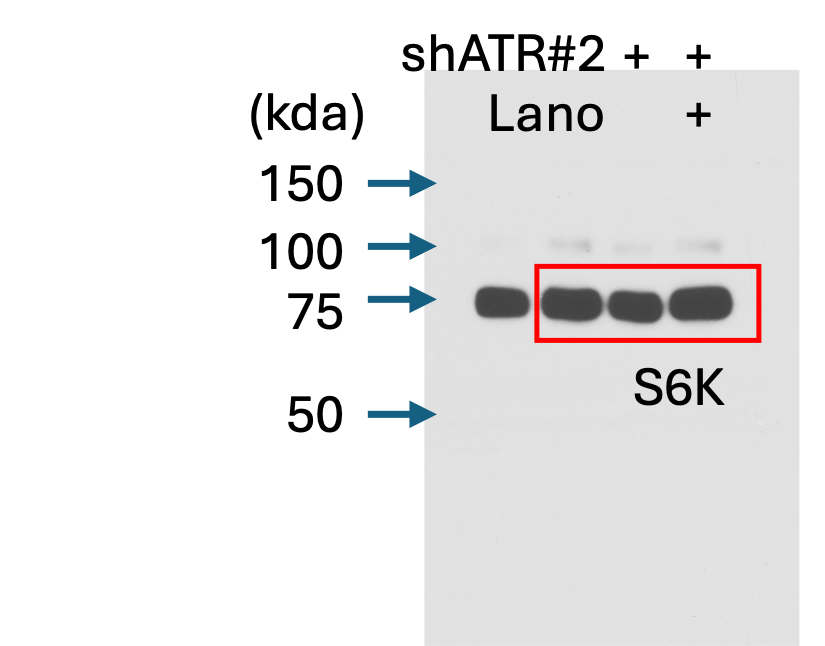

Supplement: Supplementary file 5 — Source data Fig. 4 [file 44319_2025_451_MOESM5_ESM.zip › Figure 4/Figure 4C/western S6K shATR#2 RPMI7951.tif]

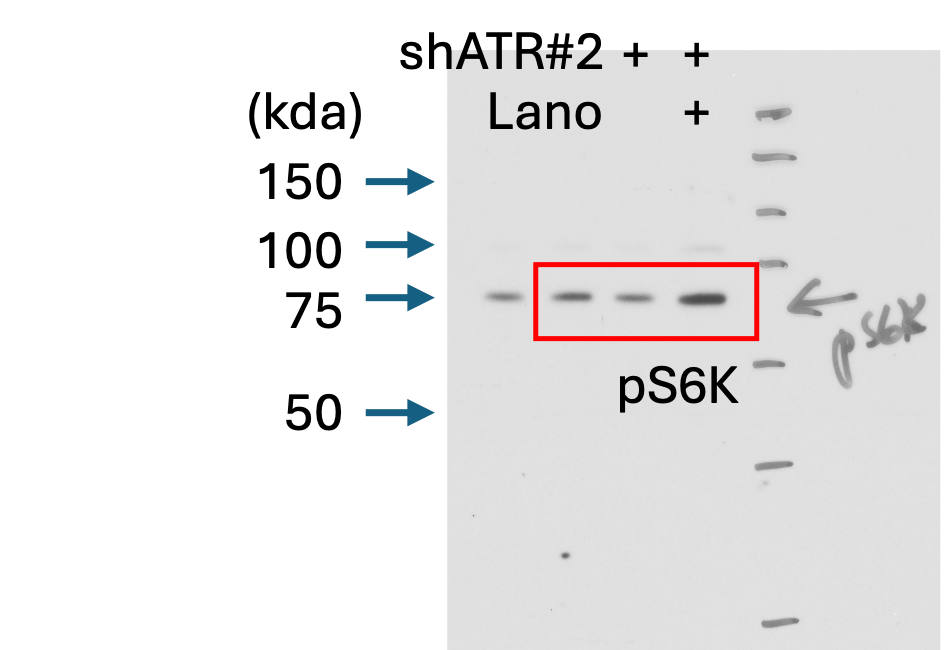

Supplement: Supplementary file 5 — Source data Fig. 4 [file 44319_2025_451_MOESM5_ESM.zip › Figure 4/Figure 4C/western pS6K shATR#2 RPMI7951.tif]

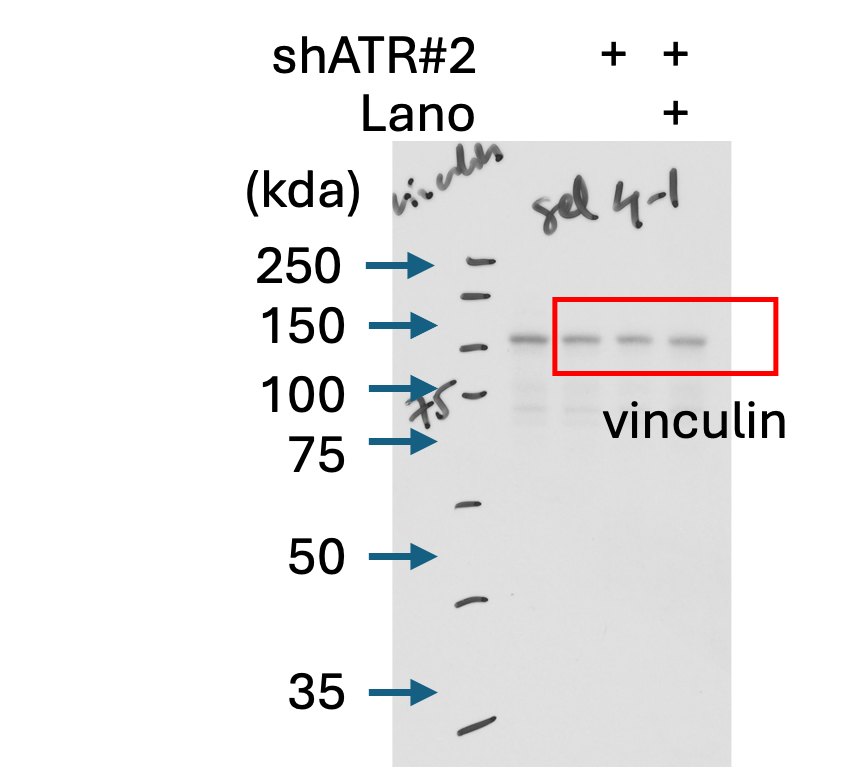

Supplement: Supplementary file 5 — Source data Fig. 4 [file 44319_2025_451_MOESM5_ESM.zip › Figure 4/Figure 4C/western vinculin shATR#2 SKMel28.tif]

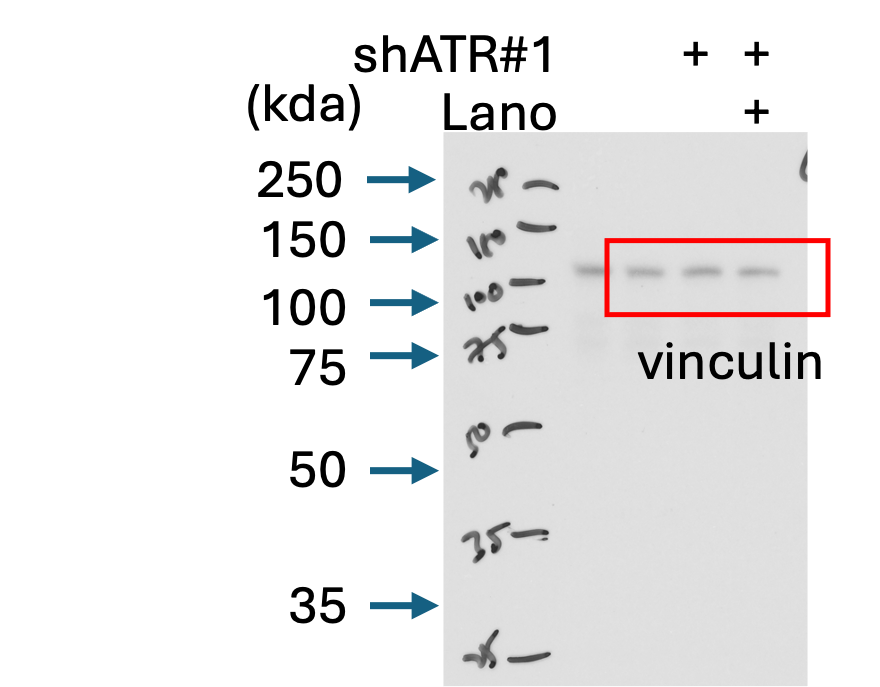

Supplement: Supplementary file 5 — Source data Fig. 4 [file 44319_2025_451_MOESM5_ESM.zip › Figure 4/Figure 4C/western vinculin shATR#1 SKMel28.tif]

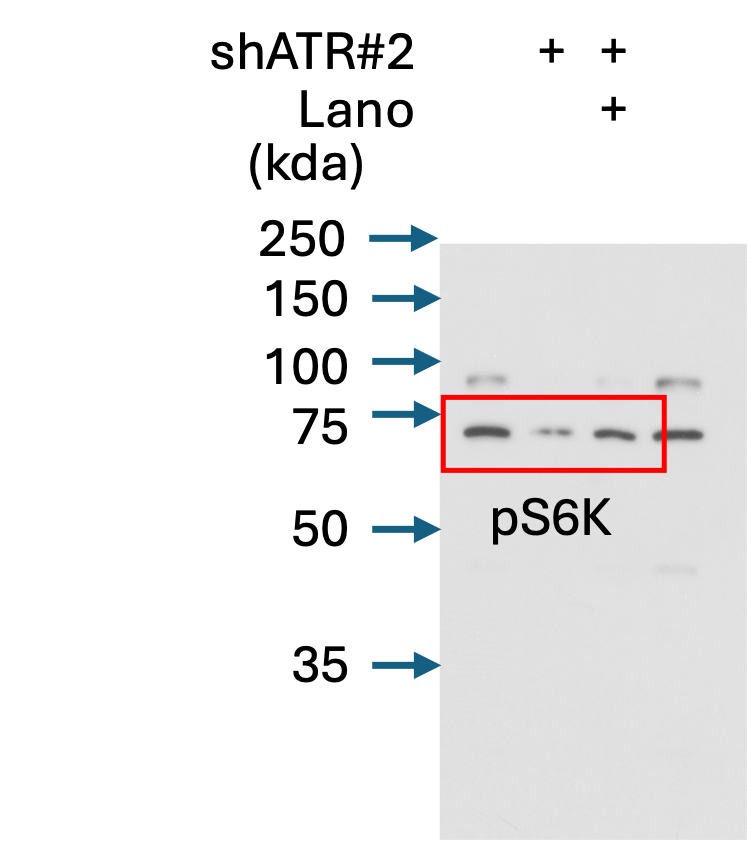

Supplement: Supplementary file 5 — Source data Fig. 4 [file 44319_2025_451_MOESM5_ESM.zip › Figure 4/Figure 4C/western pS6K shATR#2 SKMel28.tif]

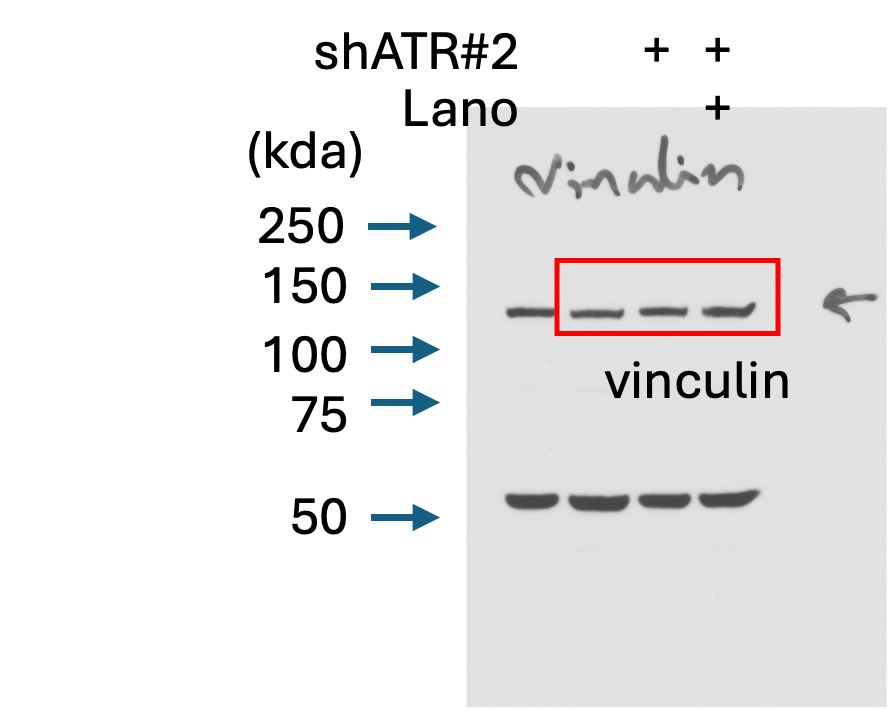

Supplement: Supplementary file 5 — Source data Fig. 4 [file 44319_2025_451_MOESM5_ESM.zip › Figure 4/Figure 4C/western vinculin shATR#2 RPMI7951.tif]

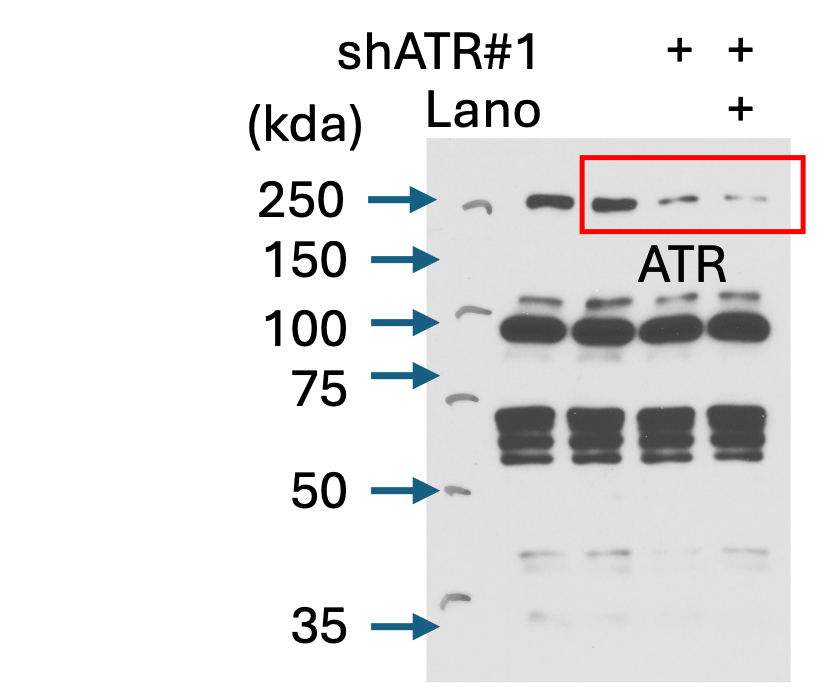

Supplement: Supplementary file 5 — Source data Fig. 4 [file 44319_2025_451_MOESM5_ESM.zip › Figure 4/Figure 4C/western ATR shATR#1 SKMel28.tif]

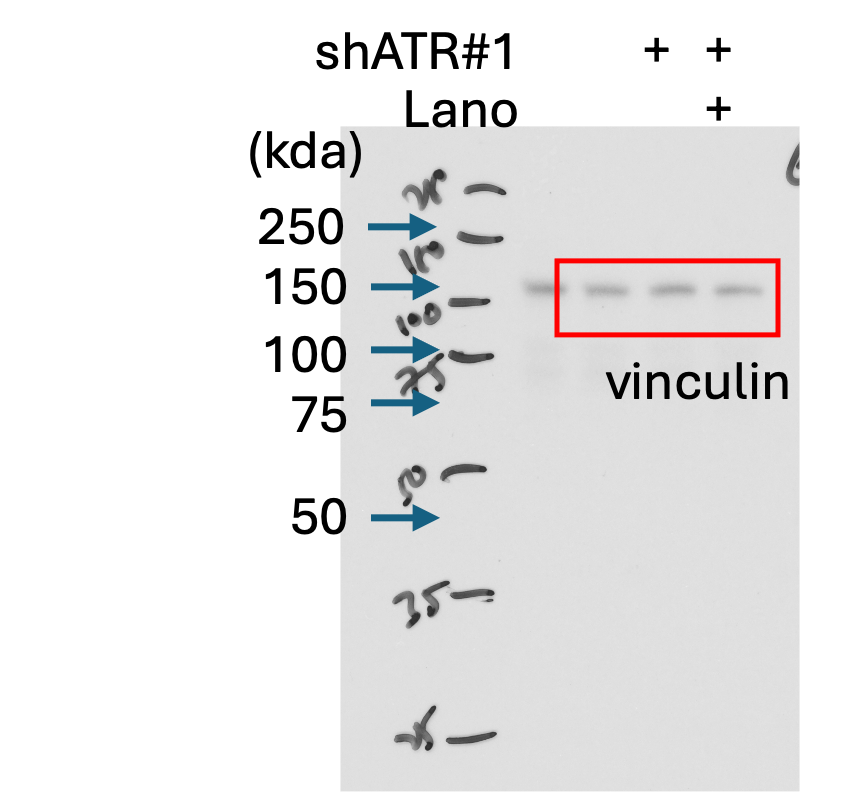

Supplement: Supplementary file 5 — Source data Fig. 4 [file 44319_2025_451_MOESM5_ESM.zip › Figure 4/Figure 4C/western vinculin shATR#1 RPMI7951.tif]

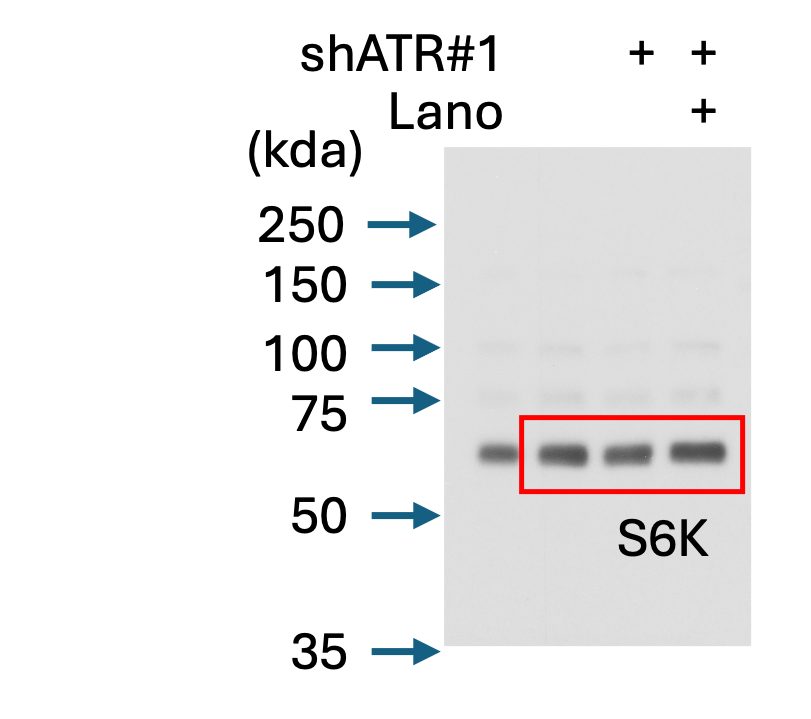

Supplement: Supplementary file 5 — Source data Fig. 4 [file 44319_2025_451_MOESM5_ESM.zip › Figure 4/Figure 4C/western S6K shATR#1 SKMel28.tif]

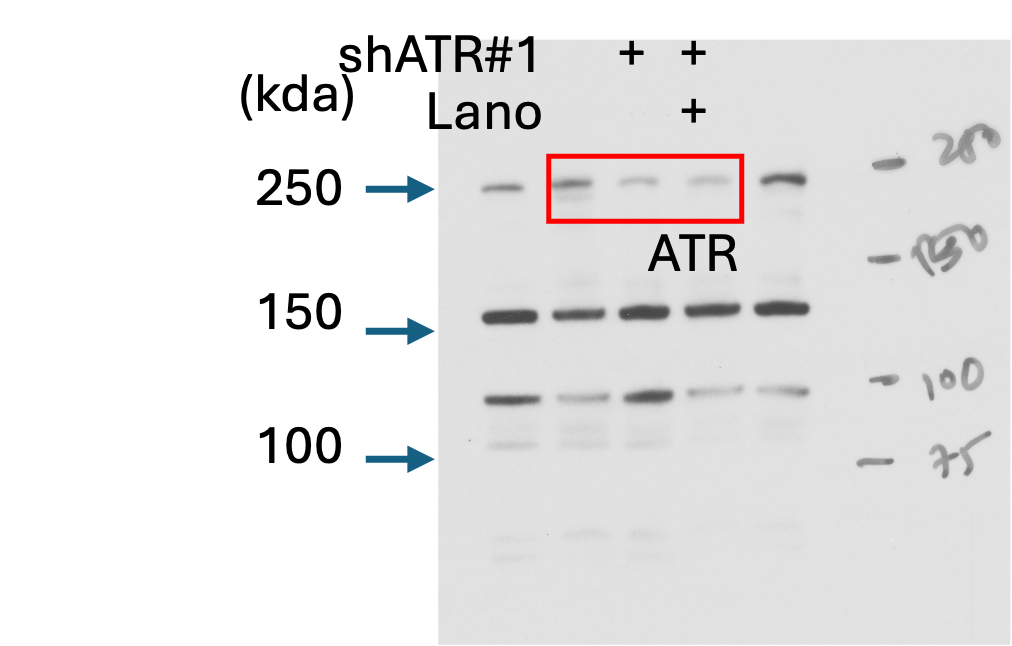

Supplement: Supplementary file 5 — Source data Fig. 4 [file 44319_2025_451_MOESM5_ESM.zip › Figure 4/Figure 4C/western ATR shATR#1 RPMI7951.tif]

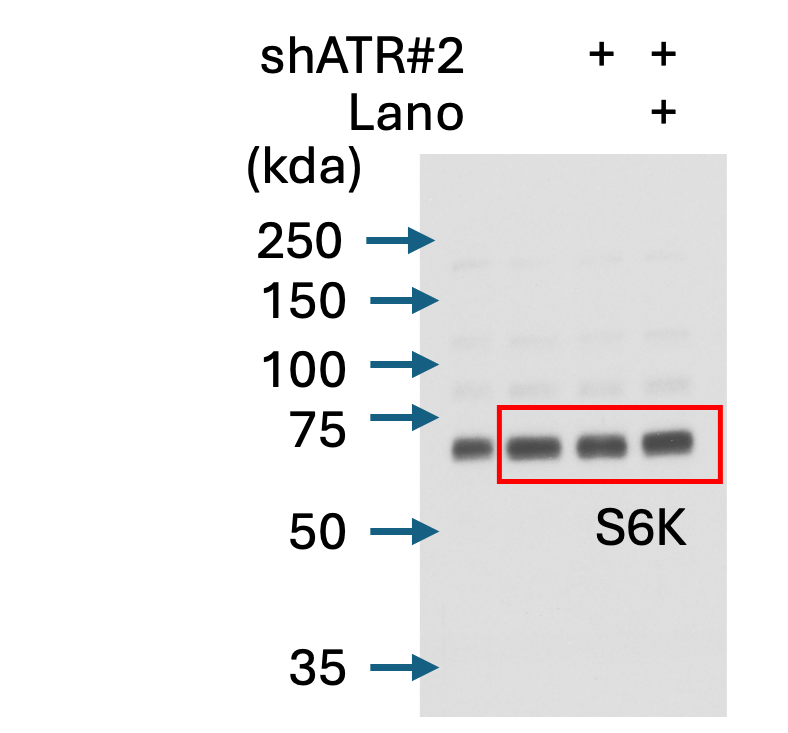

Supplement: Supplementary file 5 — Source data Fig. 4 [file 44319_2025_451_MOESM5_ESM.zip › Figure 4/Figure 4C/western S6K shATR#2 SKMel28.tif]

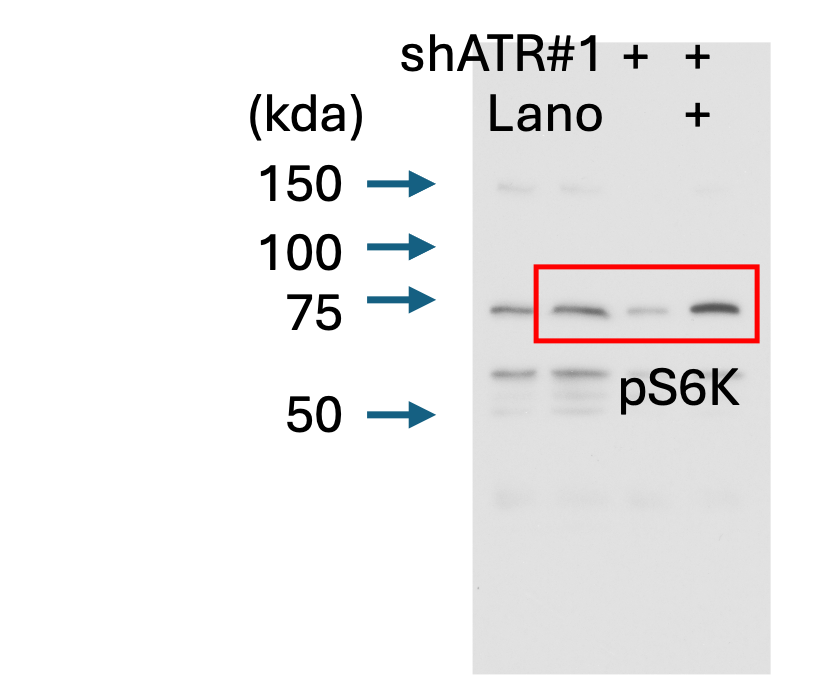

Supplement: Supplementary file 5 — Source data Fig. 4 [file 44319_2025_451_MOESM5_ESM.zip › Figure 4/Figure 4C/western pS6K shATR#1 RPMI7951.tif]

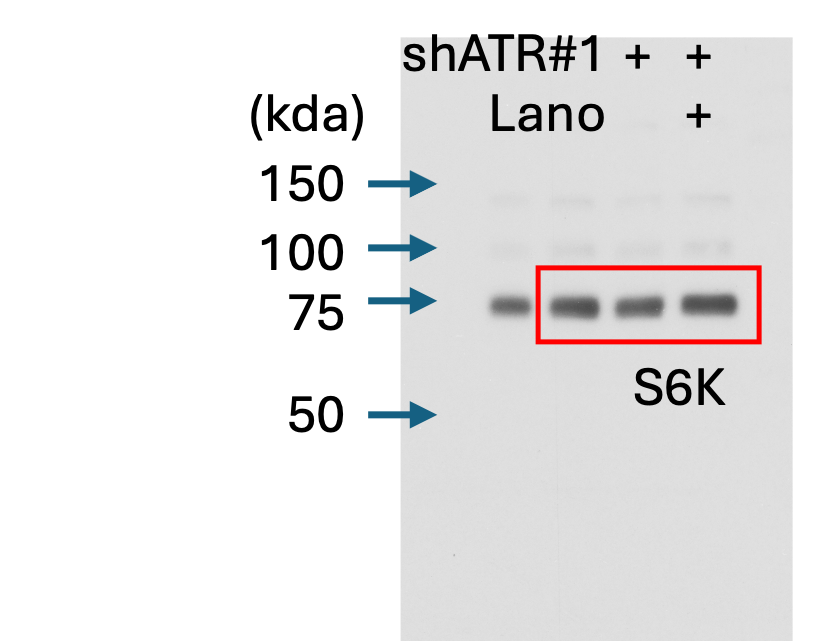

Supplement: Supplementary file 5 — Source data Fig. 4 [file 44319_2025_451_MOESM5_ESM.zip › Figure 4/Figure 4C/western S6K shATR#1 RPMI7951.tif]

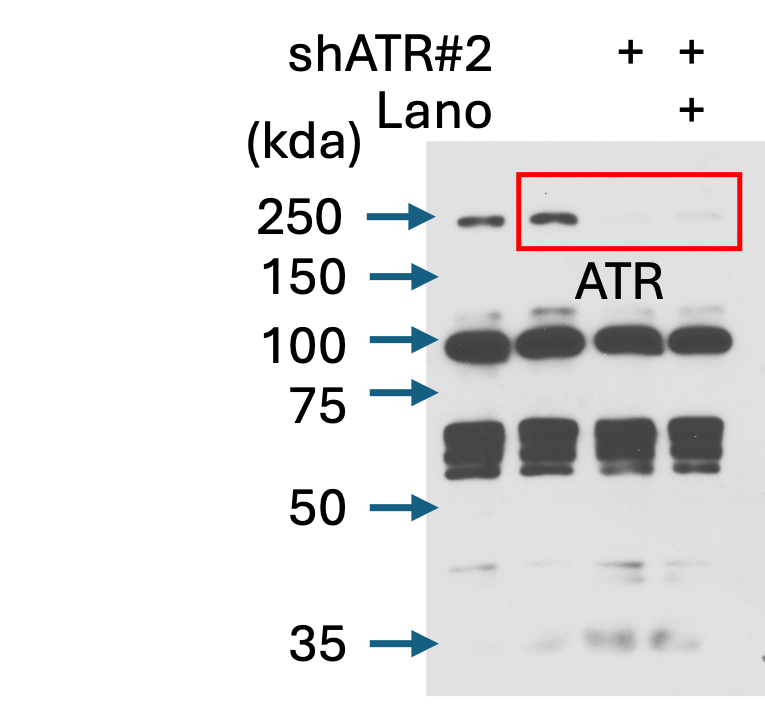

Supplement: Supplementary file 5 — Source data Fig. 4 [file 44319_2025_451_MOESM5_ESM.zip › Figure 4/Figure 4C/western ATR shATR#2 SKMel28.tif]

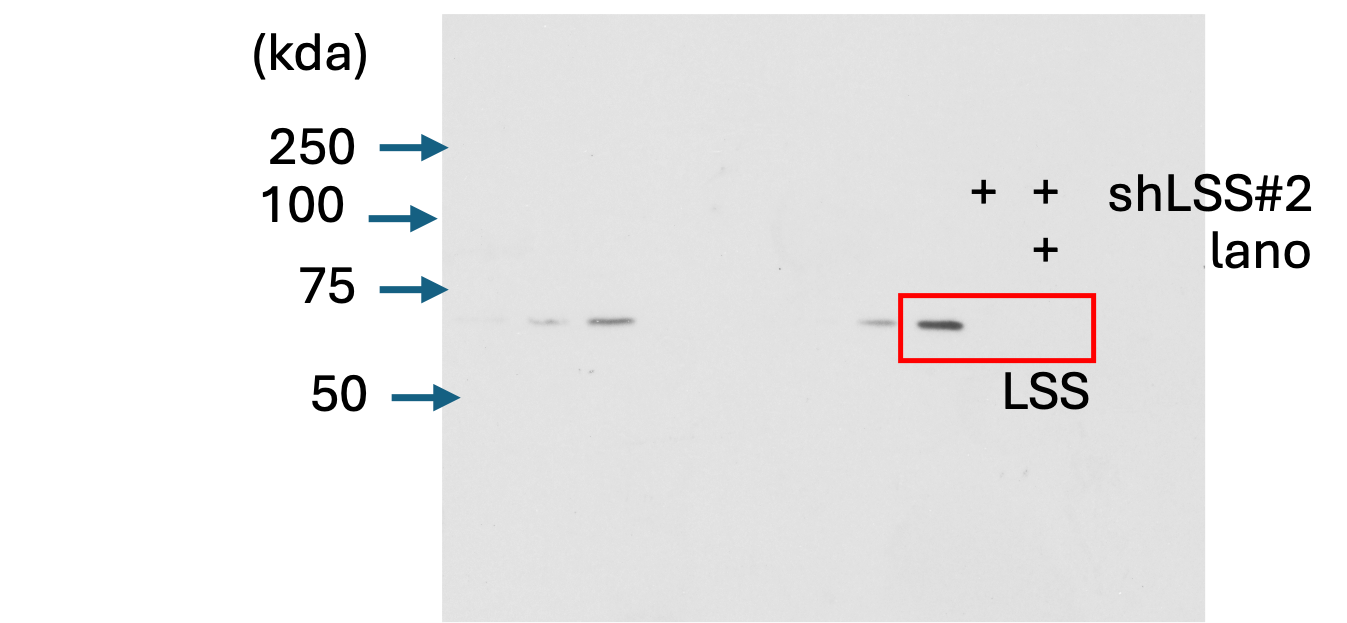

Supplement: Supplementary file 5 — Source data Fig. 4 [file 44319_2025_451_MOESM5_ESM.zip › Figure 4/Figure 4D/western LSS shLSS#2 RPMI7951.tif]

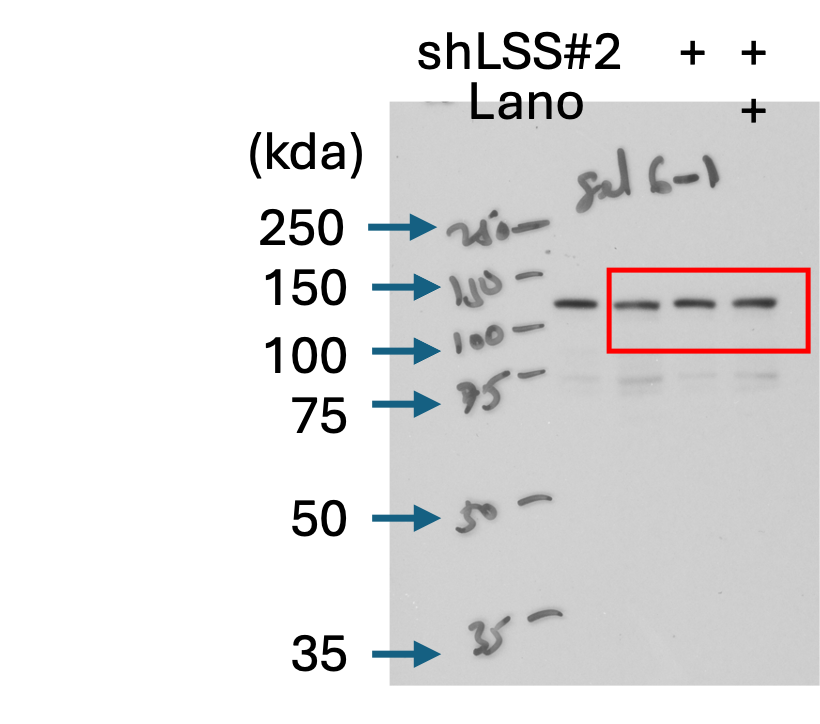

Supplement: Supplementary file 5 — Source data Fig. 4 [file 44319_2025_451_MOESM5_ESM.zip › Figure 4/Figure 4D/western vinculin shLSS#2 SKMel28.tif]
