# Supplementary figures and images for: ATR promotes mTORC1 activity via de novo cholesterol synthesis (part 2 of 2)
Source: EMBO Rep. 2025 Jun 13;26(14):3574–93. doi: 10.1038/s44319-025-00451-3 (PMC12287318; doi:10.1038/s44319-025-00451-3)

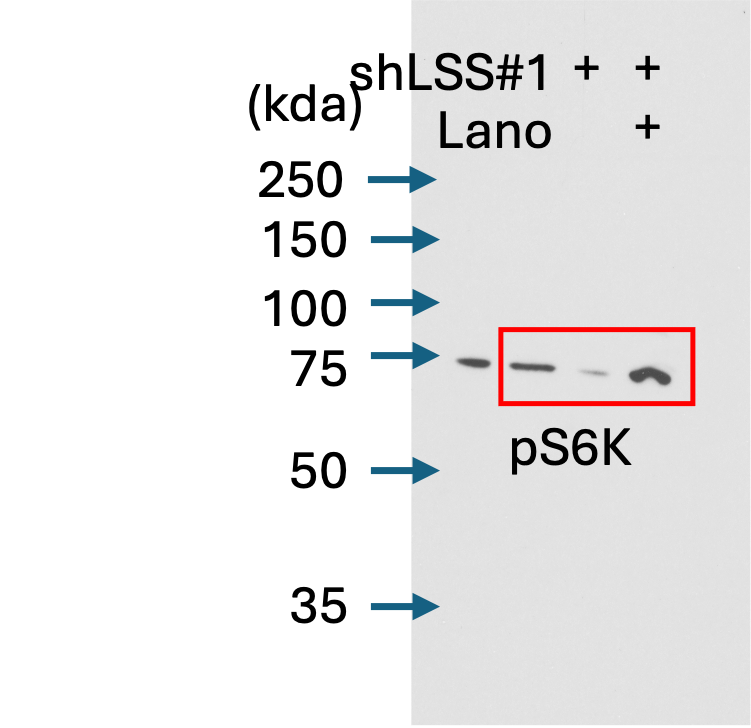

Supplement: Supplementary file 5 — Source data Fig. 4 [file 44319_2025_451_MOESM5_ESM.zip › Figure 4/Figure 4D/western pS6K shLSS#1 SKMel28.tif]

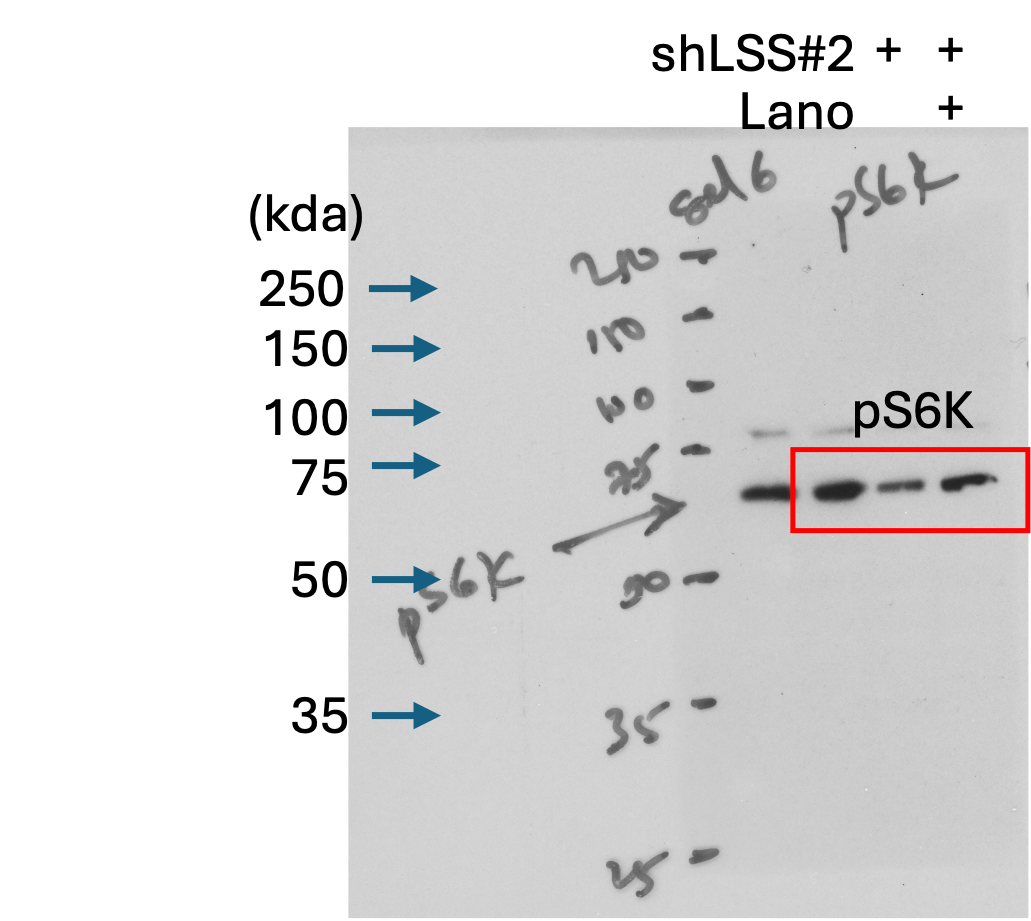

Supplement: Supplementary file 5 — Source data Fig. 4 [file 44319_2025_451_MOESM5_ESM.zip › Figure 4/Figure 4D/western pS6K shLSS#2 SKMel28.tif]

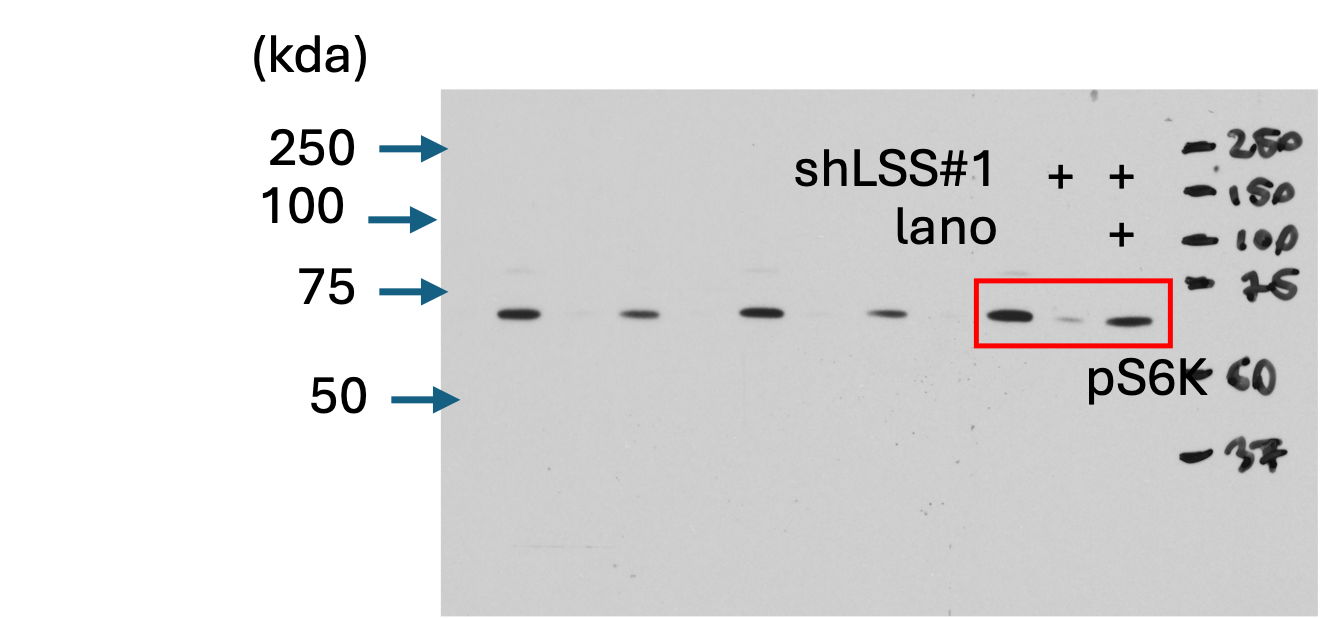

Supplement: Supplementary file 5 — Source data Fig. 4 [file 44319_2025_451_MOESM5_ESM.zip › Figure 4/Figure 4D/western pS6K shLSS#1 RPMI7951.tif]

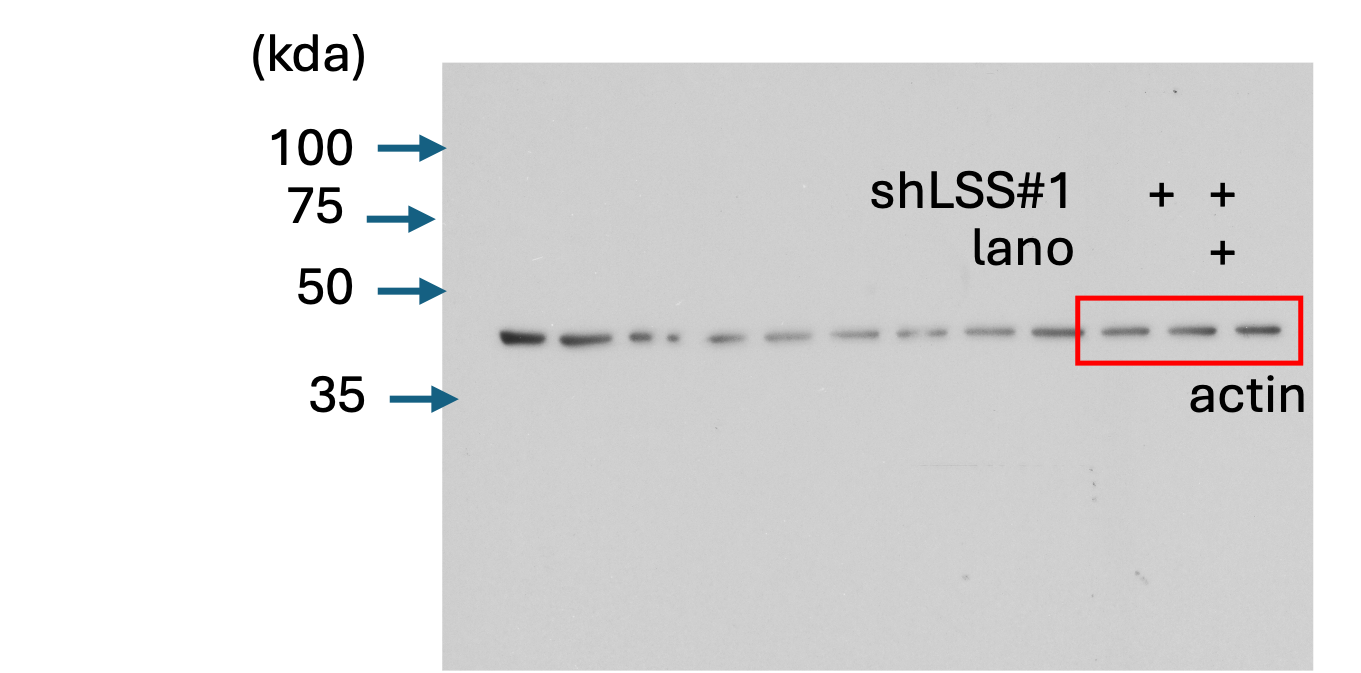

Supplement: Supplementary file 5 — Source data Fig. 4 [file 44319_2025_451_MOESM5_ESM.zip › Figure 4/Figure 4D/western actin shLSS#1 RPMI7951.tif]

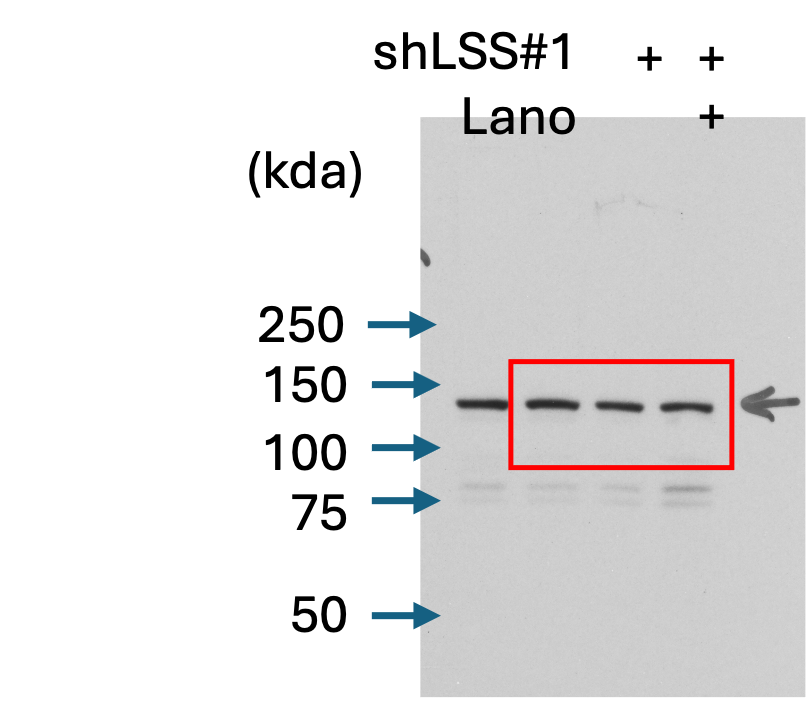

Supplement: Supplementary file 5 — Source data Fig. 4 [file 44319_2025_451_MOESM5_ESM.zip › Figure 4/Figure 4D/western vinculin shLSS#1 SKMel28.tif]

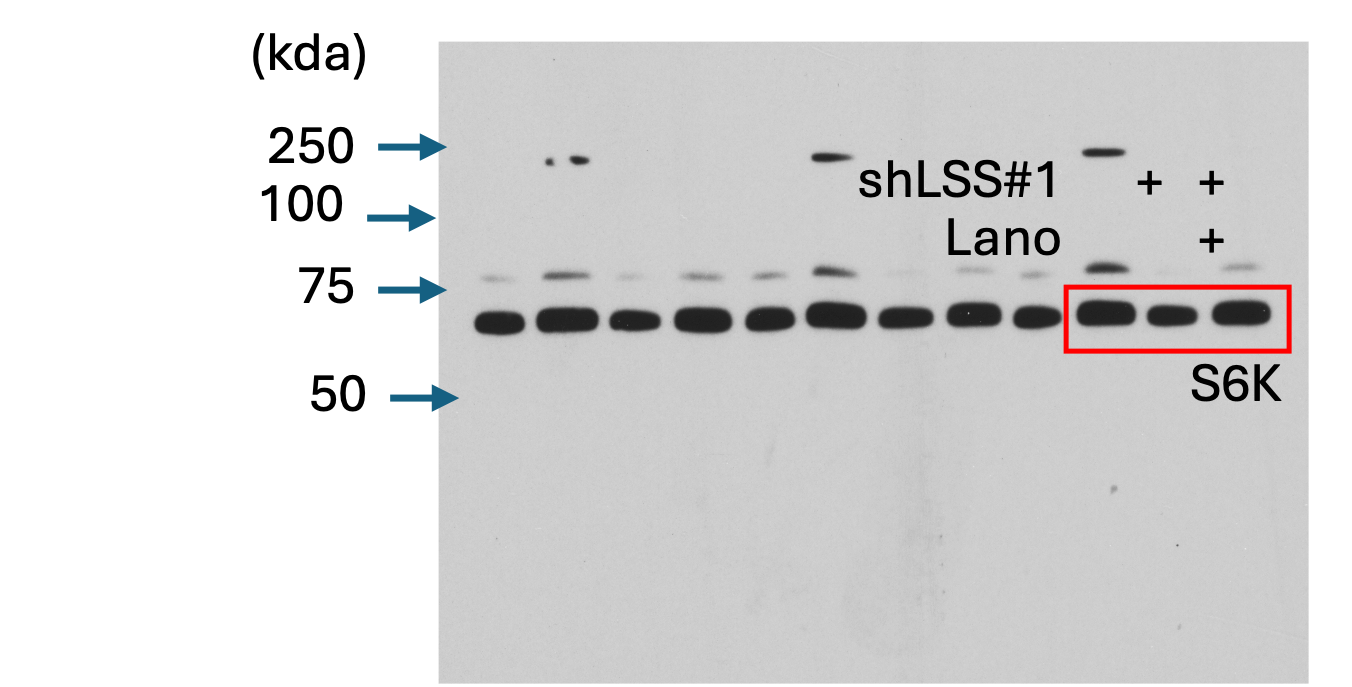

Supplement: Supplementary file 5 — Source data Fig. 4 [file 44319_2025_451_MOESM5_ESM.zip › Figure 4/Figure 4D/western S6K shLSS#1 RPMI7951.tif]

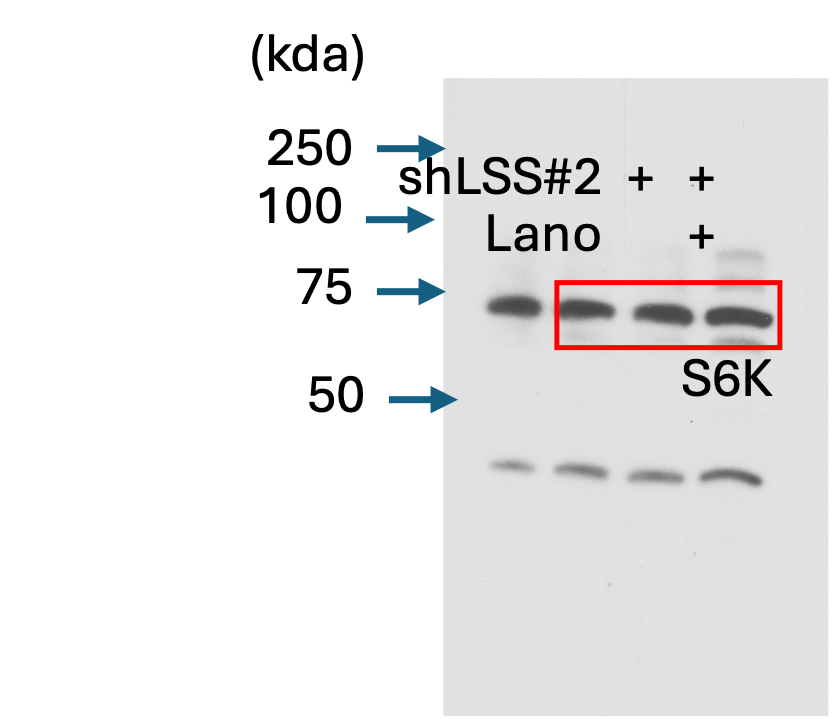

Supplement: Supplementary file 5 — Source data Fig. 4 [file 44319_2025_451_MOESM5_ESM.zip › Figure 4/Figure 4D/western S6K shLSS#2 RPMI7951.tif]

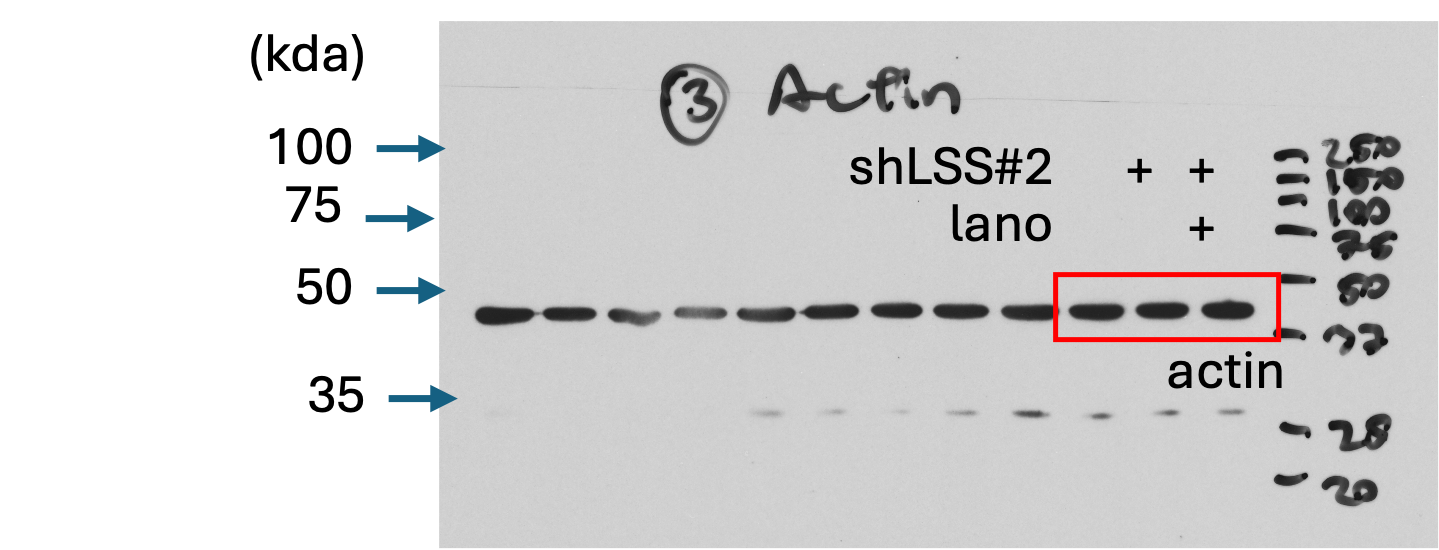

Supplement: Supplementary file 5 — Source data Fig. 4 [file 44319_2025_451_MOESM5_ESM.zip › Figure 4/Figure 4D/western actin shLSS#2 RPMI7951.tif]

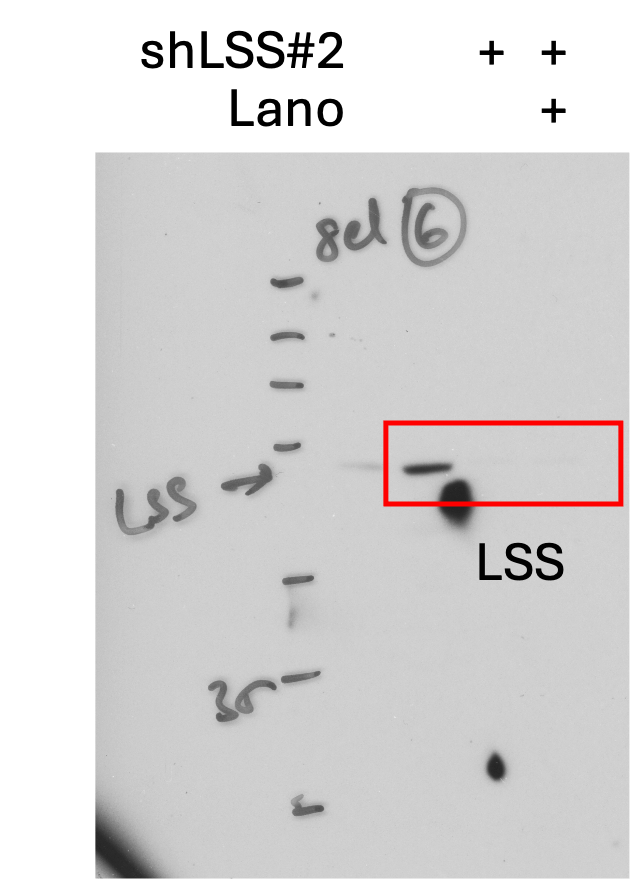

Supplement: Supplementary file 5 — Source data Fig. 4 [file 44319_2025_451_MOESM5_ESM.zip › Figure 4/Figure 4D/western LSS shLSS#2 SKMel28.tif]

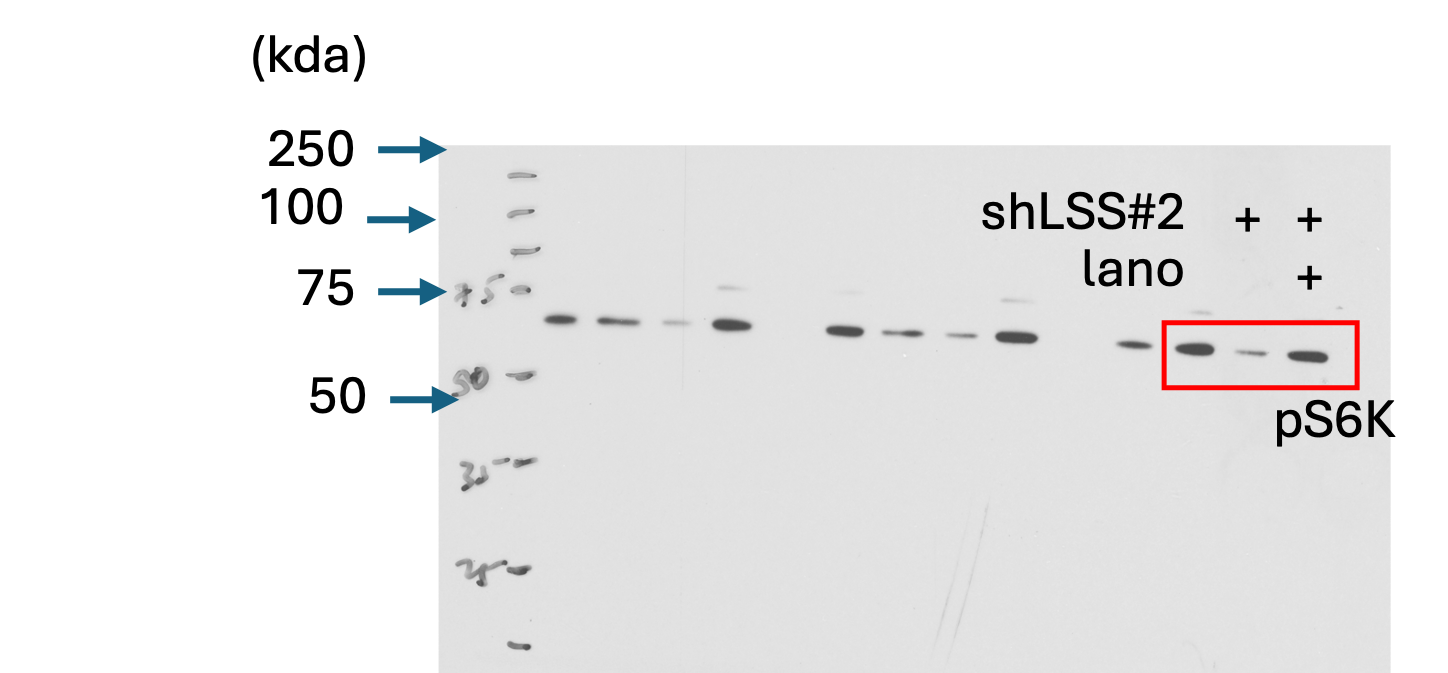

Supplement: Supplementary file 5 — Source data Fig. 4 [file 44319_2025_451_MOESM5_ESM.zip › Figure 4/Figure 4D/western pS6K shLSS#2 RPMI7951.tif]

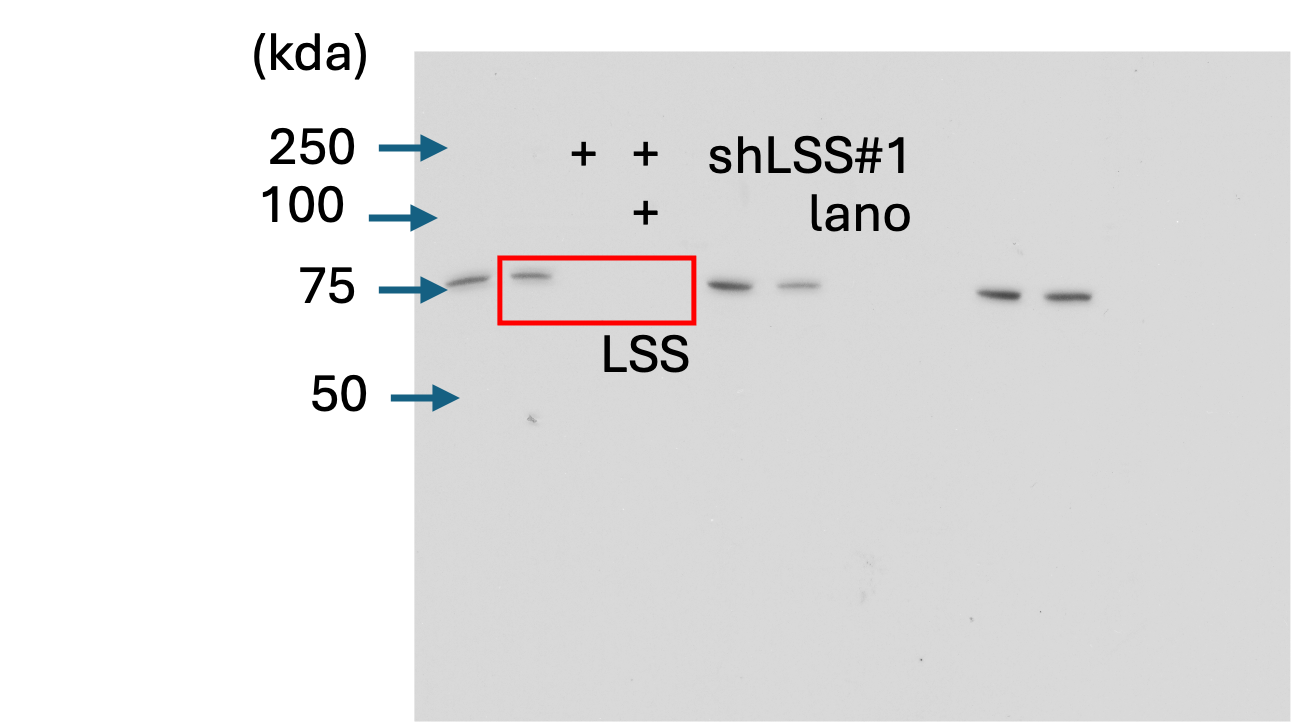

Supplement: Supplementary file 5 — Source data Fig. 4 [file 44319_2025_451_MOESM5_ESM.zip › Figure 4/Figure 4D/western LSS shLSS#1 RPMI7951.tif]

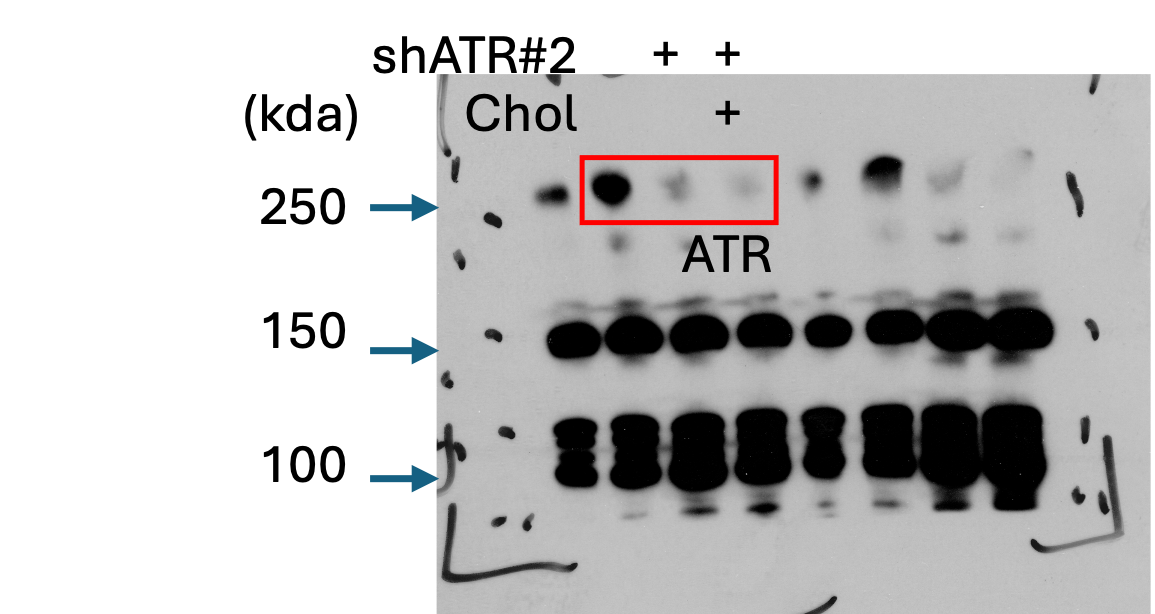

Supplement: Supplementary file 5 — Source data Fig. 4 [file 44319_2025_451_MOESM5_ESM.zip › Figure 4/Figure 4A/western ATR shATR#2 RPMI7951.tif]

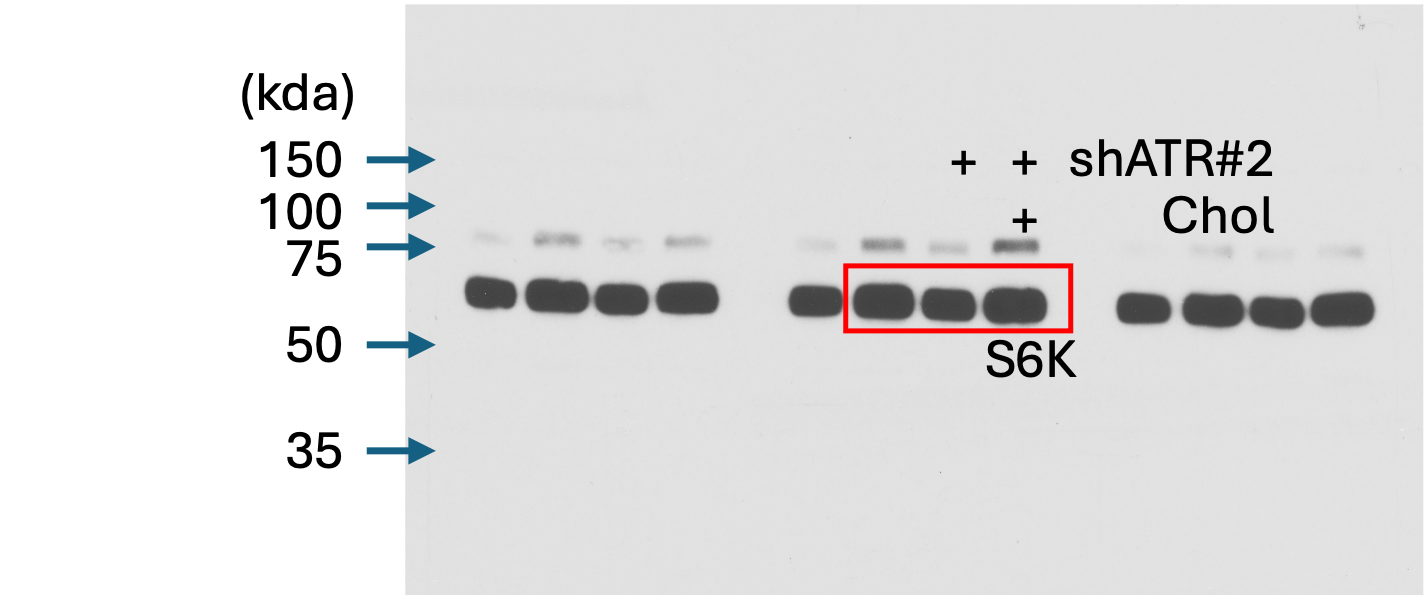

Supplement: Supplementary file 5 — Source data Fig. 4 [file 44319_2025_451_MOESM5_ESM.zip › Figure 4/Figure 4A/western S6K shATR#2 RPMI7951.tif]

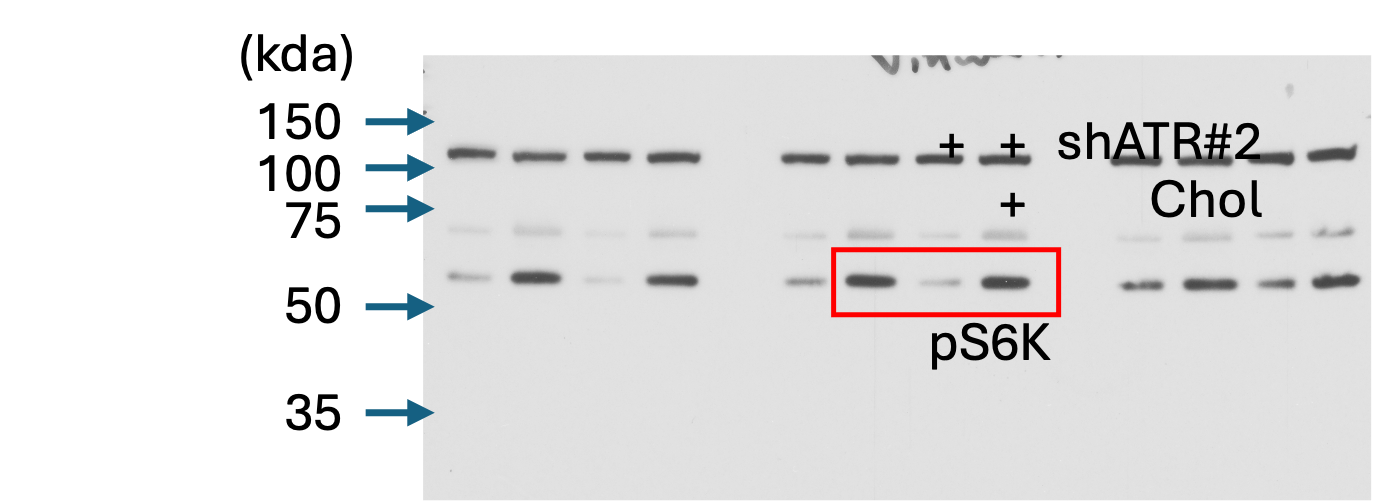

Supplement: Supplementary file 5 — Source data Fig. 4 [file 44319_2025_451_MOESM5_ESM.zip › Figure 4/Figure 4A/western pS6K shATR#2 RPMI7951.tif]

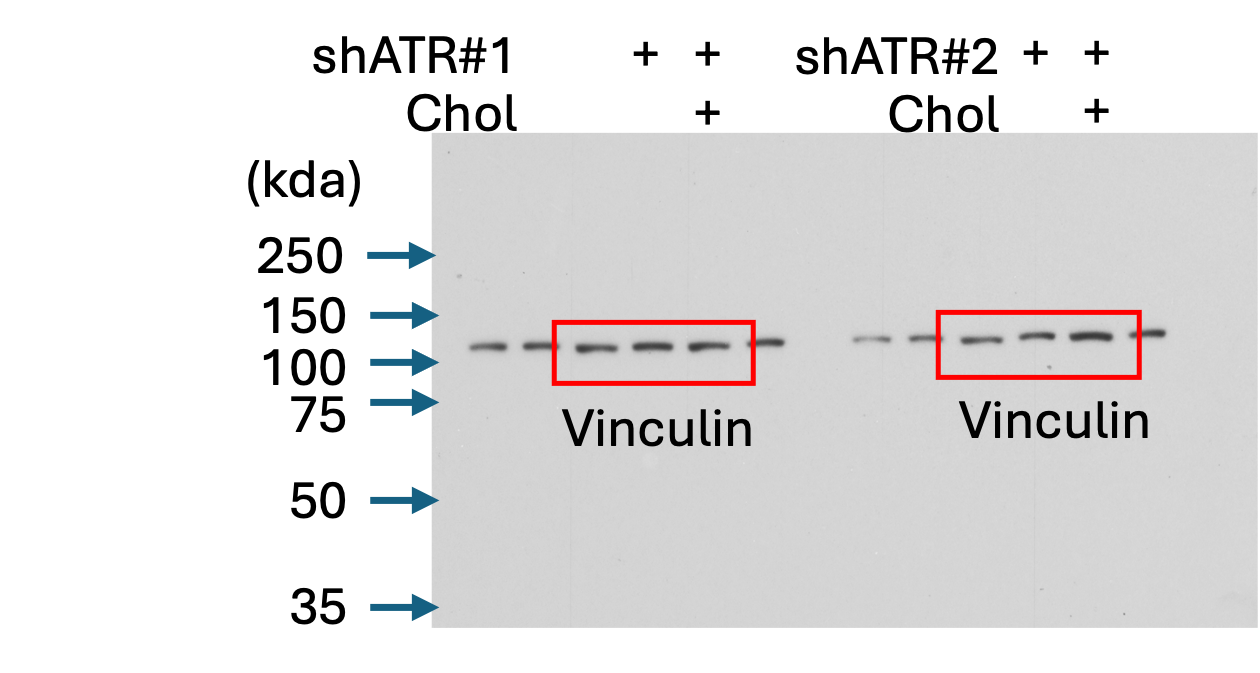

Supplement: Supplementary file 5 — Source data Fig. 4 [file 44319_2025_451_MOESM5_ESM.zip › Figure 4/Figure 4A/western vinculin_SKMel28.tif]

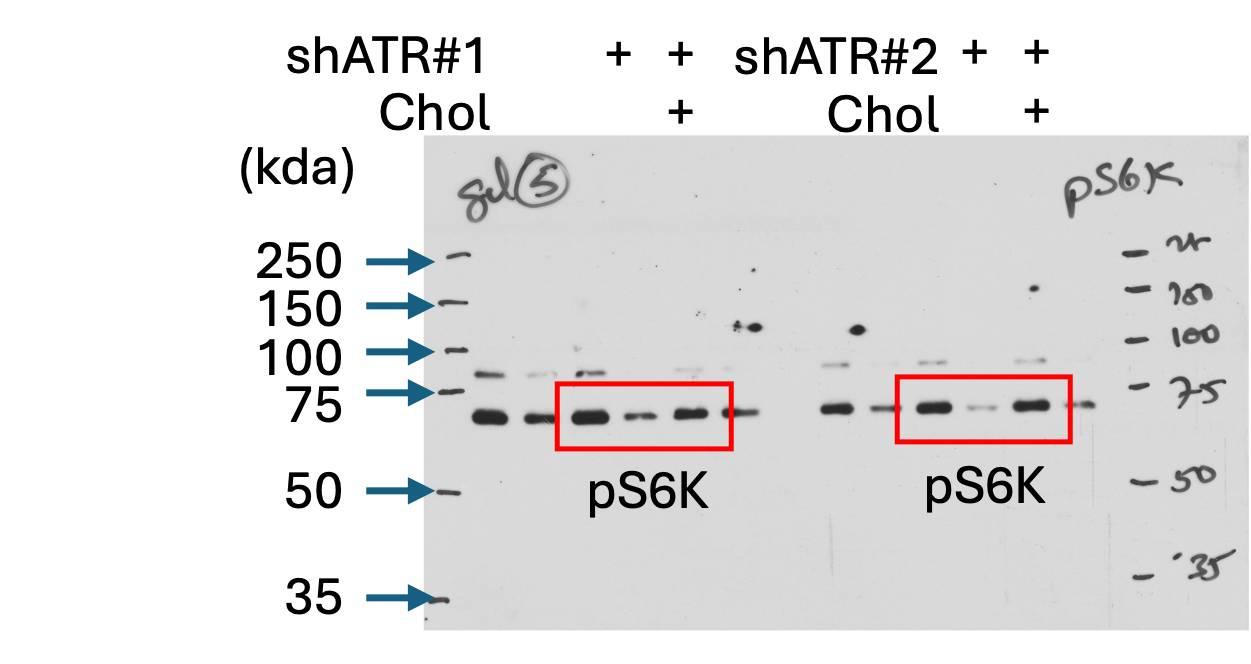

Supplement: Supplementary file 5 — Source data Fig. 4 [file 44319_2025_451_MOESM5_ESM.zip › Figure 4/Figure 4A/western pS6K_SKMel28.tif]

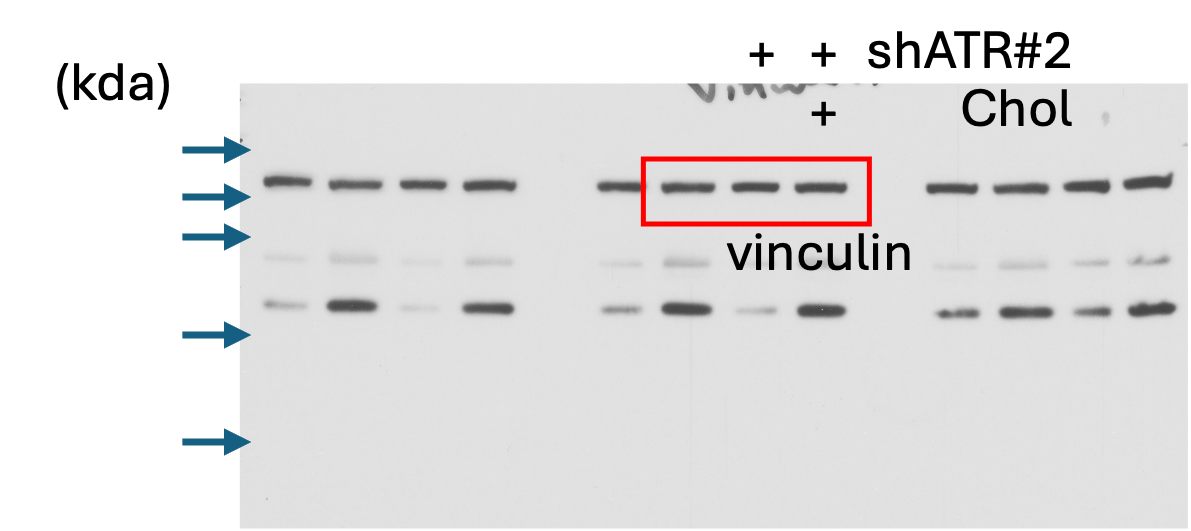

Supplement: Supplementary file 5 — Source data Fig. 4 [file 44319_2025_451_MOESM5_ESM.zip › Figure 4/Figure 4A/western vinculin shATR#2 RPMI7951.tif]

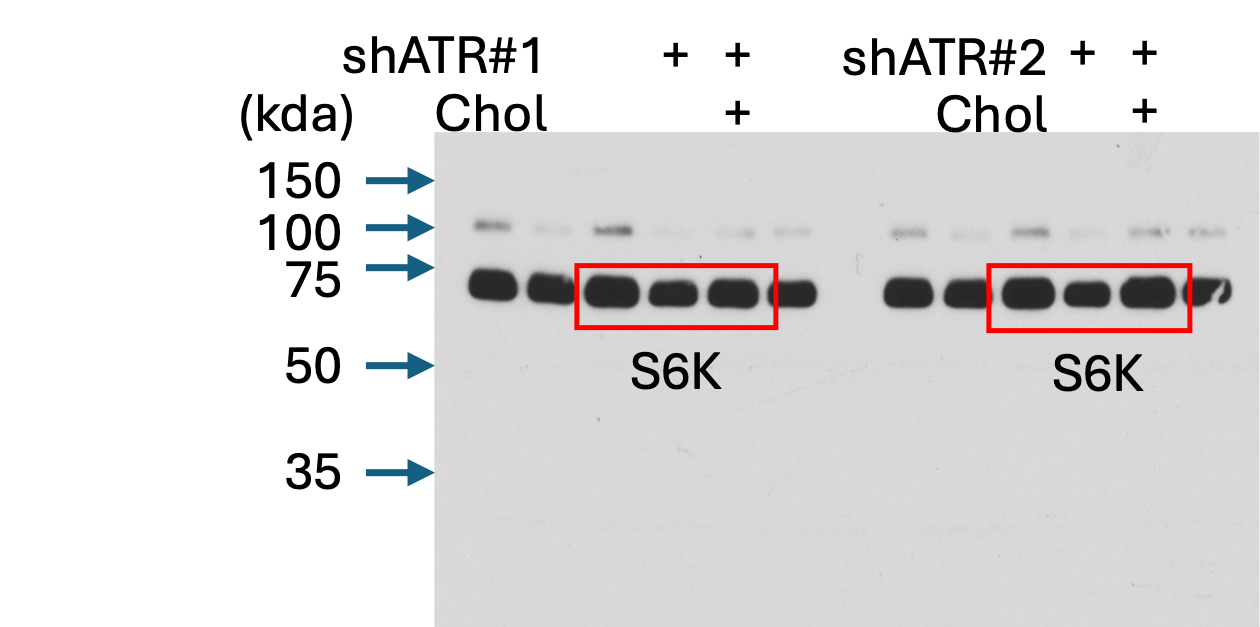

Supplement: Supplementary file 5 — Source data Fig. 4 [file 44319_2025_451_MOESM5_ESM.zip › Figure 4/Figure 4A/western S6K_SKMel28.tif]

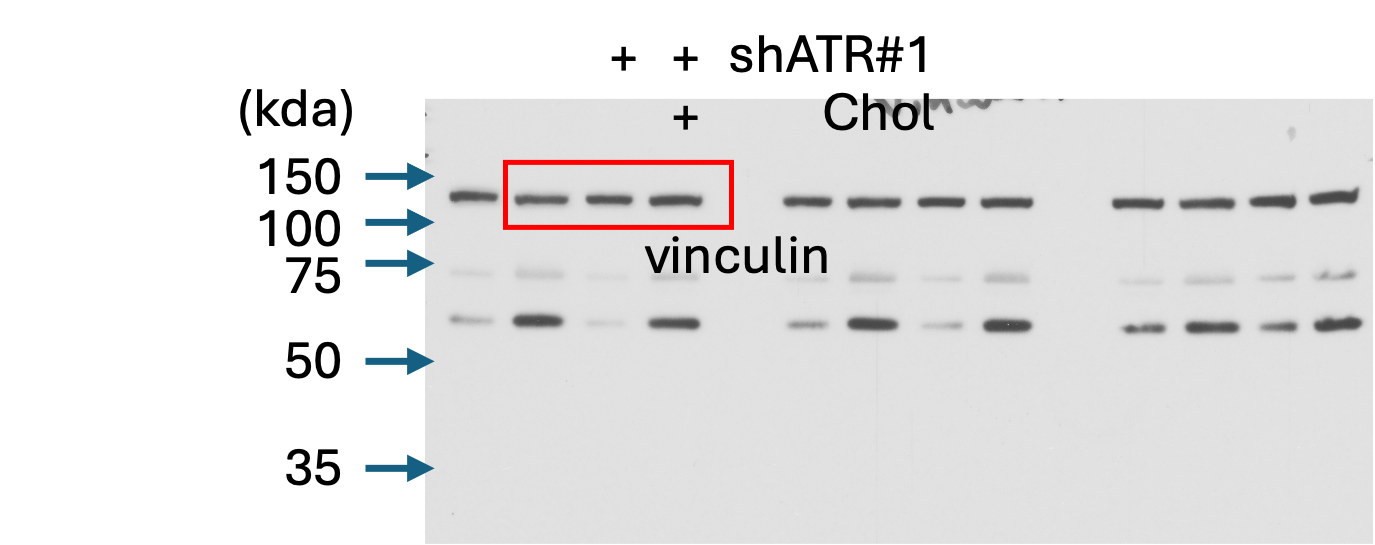

Supplement: Supplementary file 5 — Source data Fig. 4 [file 44319_2025_451_MOESM5_ESM.zip › Figure 4/Figure 4A/western vinculin shATR#1 RPMI7951.tif]

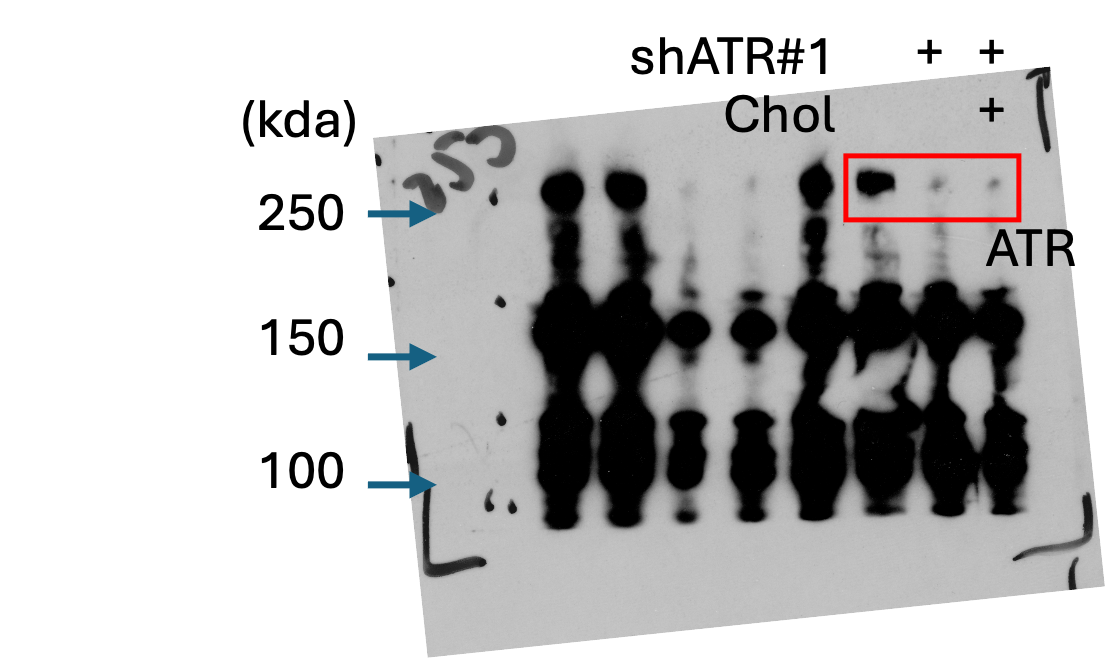

Supplement: Supplementary file 5 — Source data Fig. 4 [file 44319_2025_451_MOESM5_ESM.zip › Figure 4/Figure 4A/western ATR shATR#1 RPMI7951.tif]

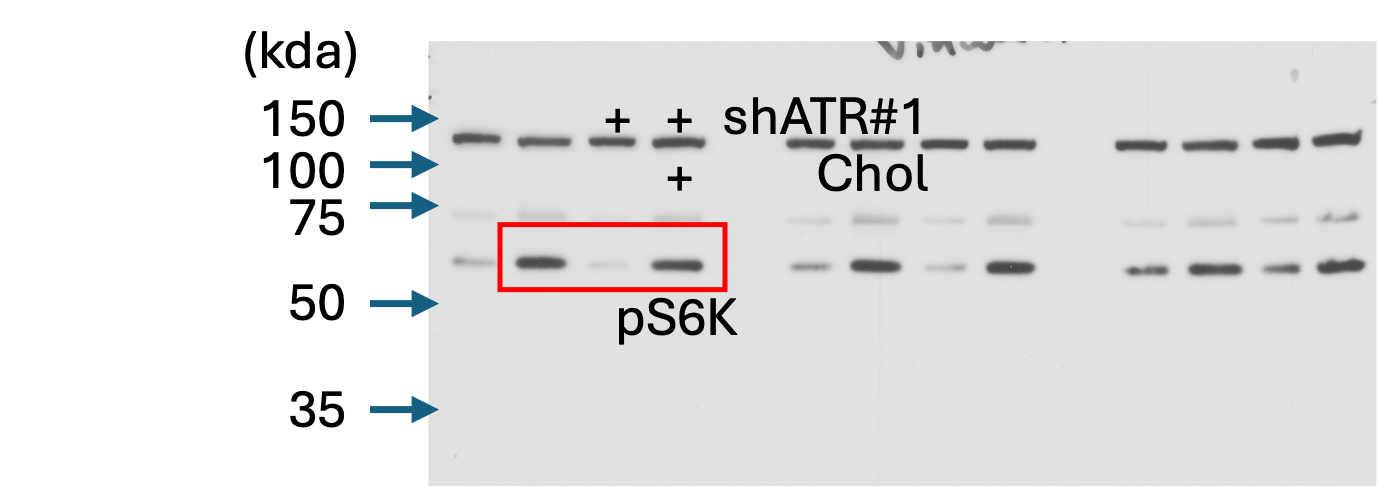

Supplement: Supplementary file 5 — Source data Fig. 4 [file 44319_2025_451_MOESM5_ESM.zip › Figure 4/Figure 4A/western pS6K shATR#1 RPMI7951.tif]

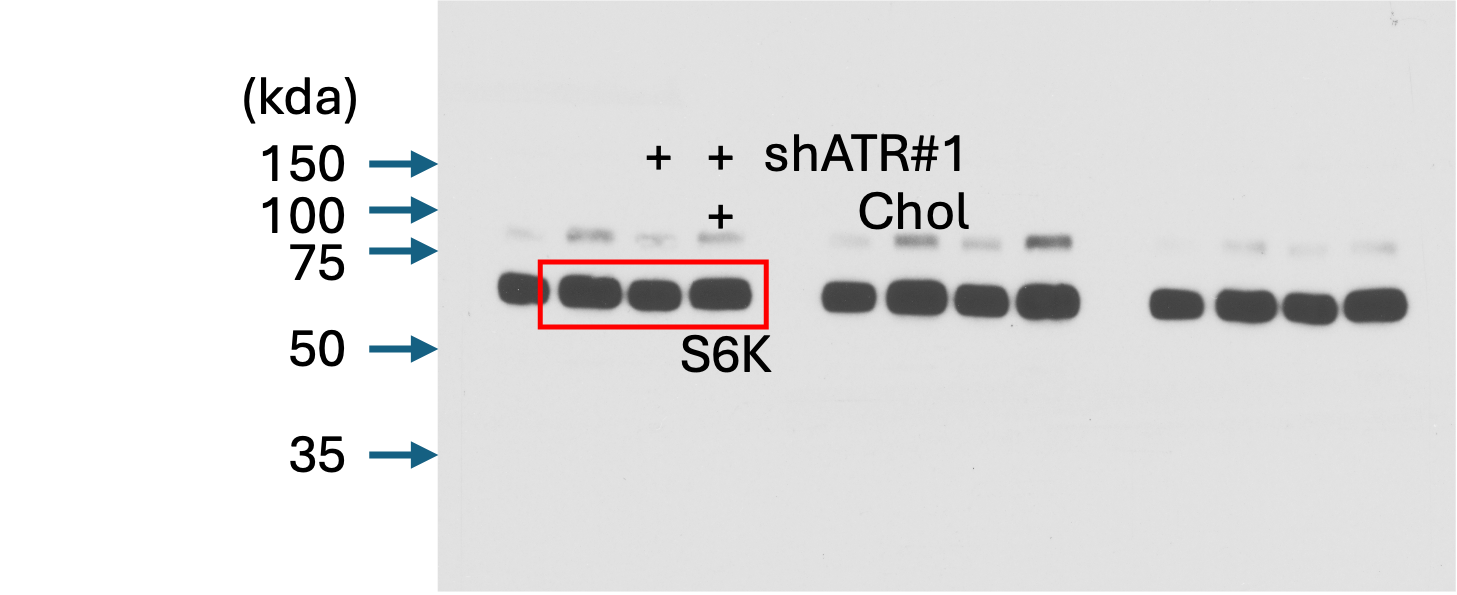

Supplement: Supplementary file 5 — Source data Fig. 4 [file 44319_2025_451_MOESM5_ESM.zip › Figure 4/Figure 4A/western S6K shATR#1 RPMI7951.tif]

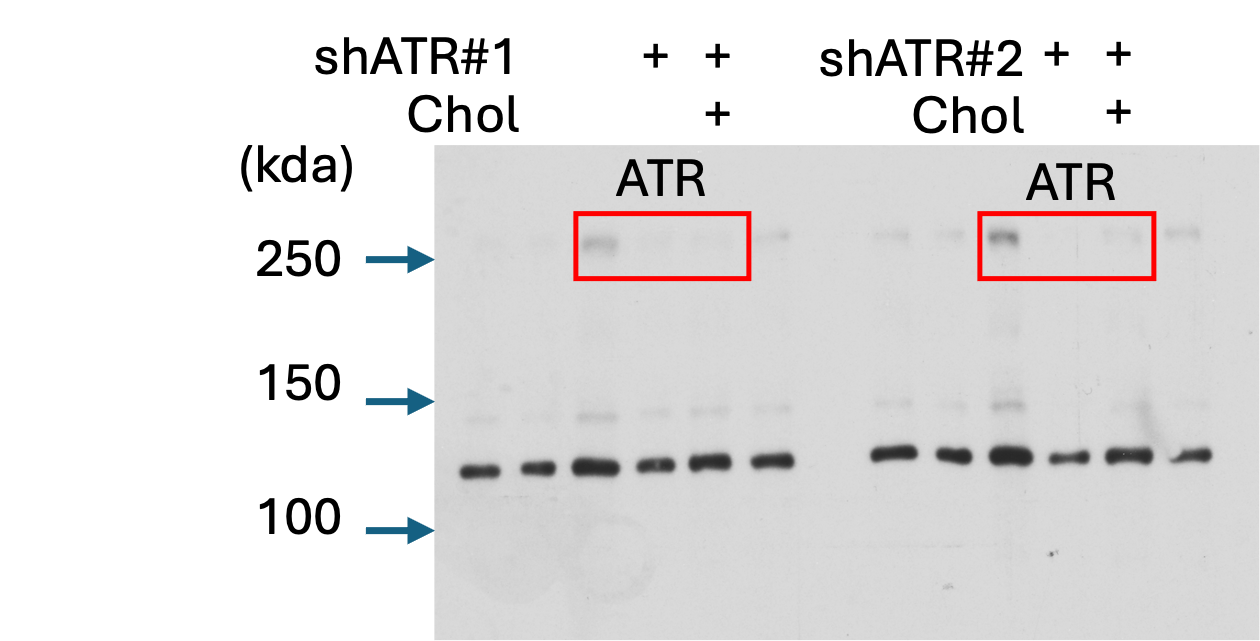

Supplement: Supplementary file 5 — Source data Fig. 4 [file 44319_2025_451_MOESM5_ESM.zip › Figure 4/Figure 4A/western ATR_SKMel28.tif]

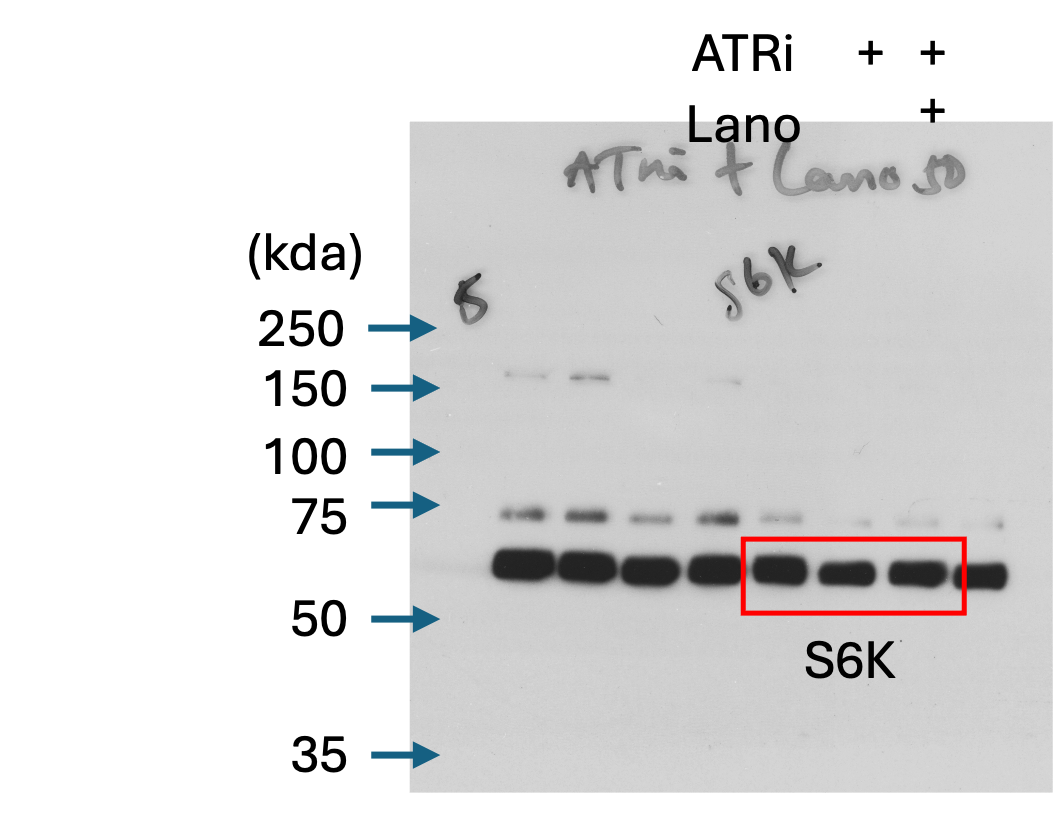

Supplement: Supplementary file 5 — Source data Fig. 4 [file 44319_2025_451_MOESM5_ESM.zip › Figure 4/Figure 4F/western S6K.tif]

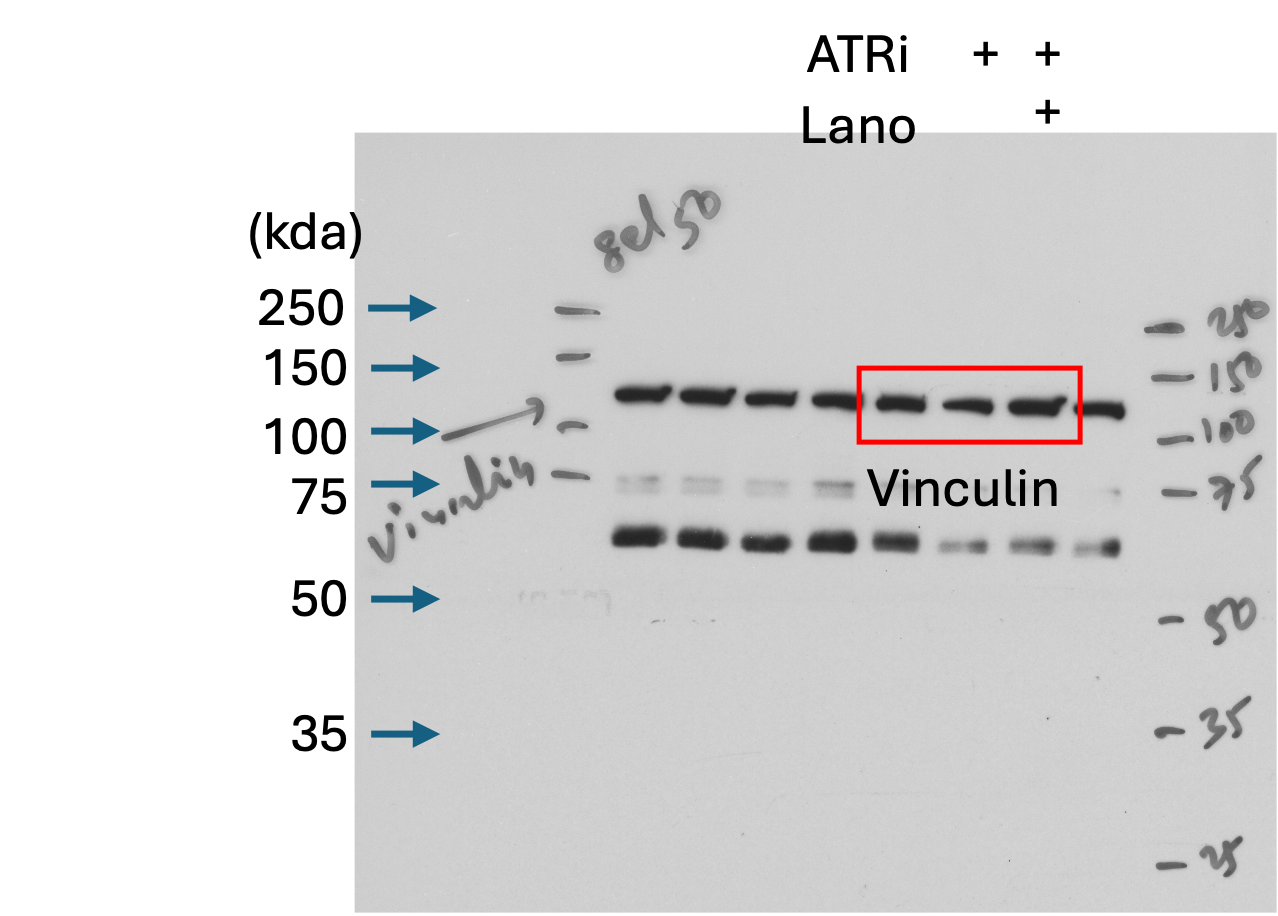

Supplement: Supplementary file 5 — Source data Fig. 4 [file 44319_2025_451_MOESM5_ESM.zip › Figure 4/Figure 4F/western vinculin.tif]

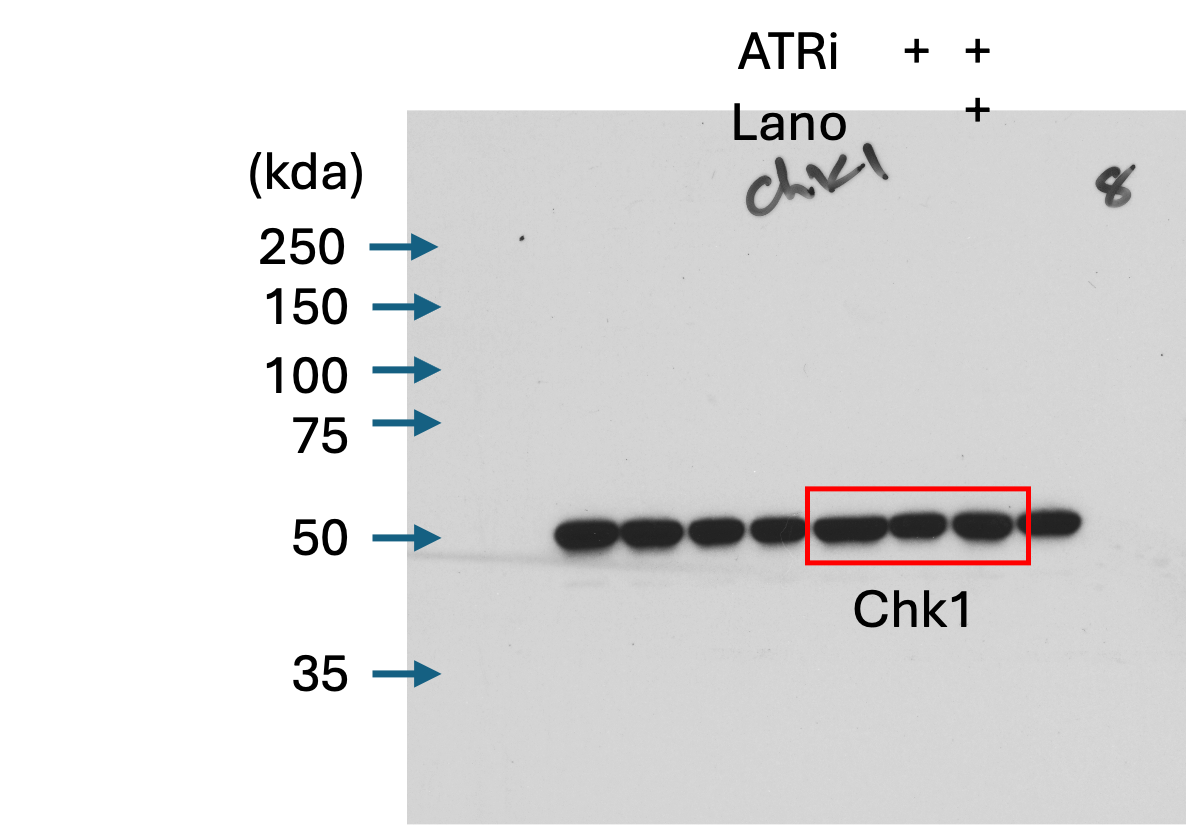

Supplement: Supplementary file 5 — Source data Fig. 4 [file 44319_2025_451_MOESM5_ESM.zip › Figure 4/Figure 4F/western Chk1.tif]

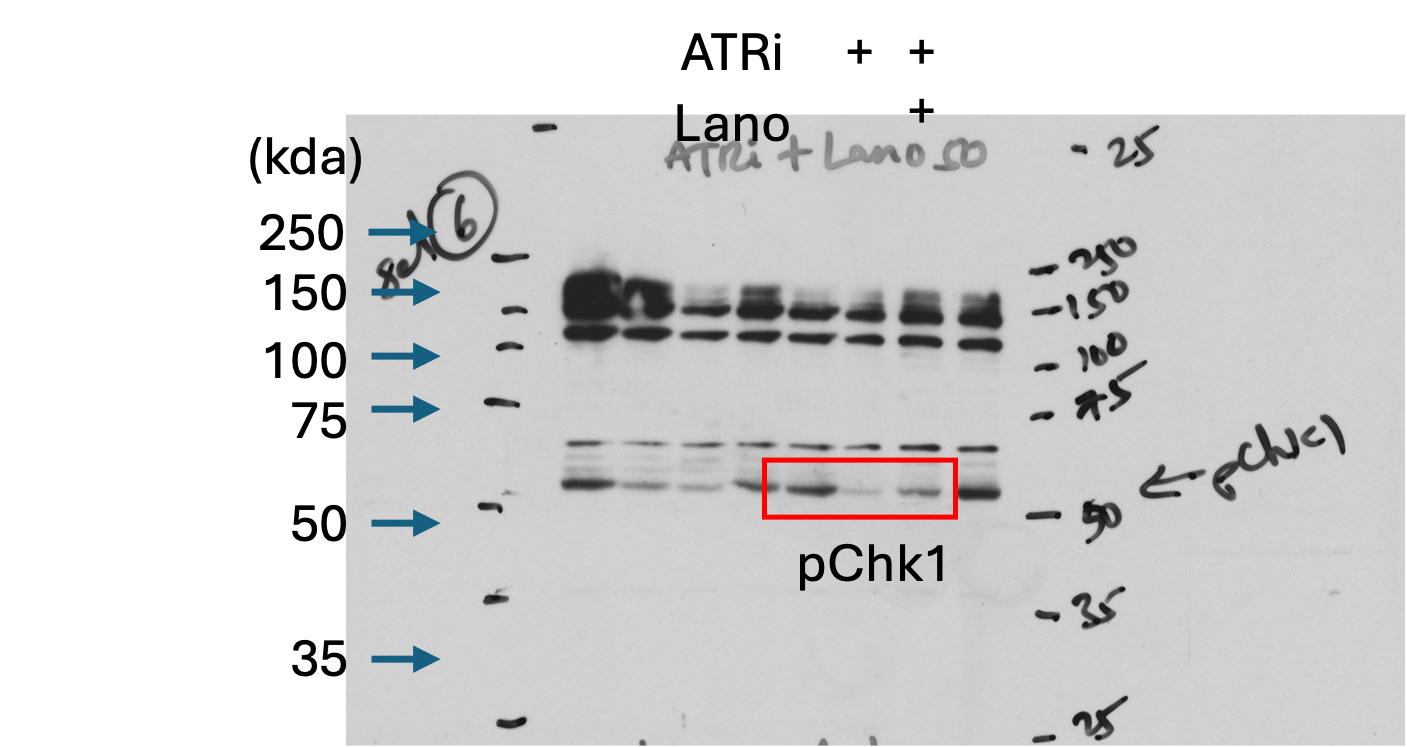

Supplement: Supplementary file 5 — Source data Fig. 4 [file 44319_2025_451_MOESM5_ESM.zip › Figure 4/Figure 4F/western pChk1.tif]

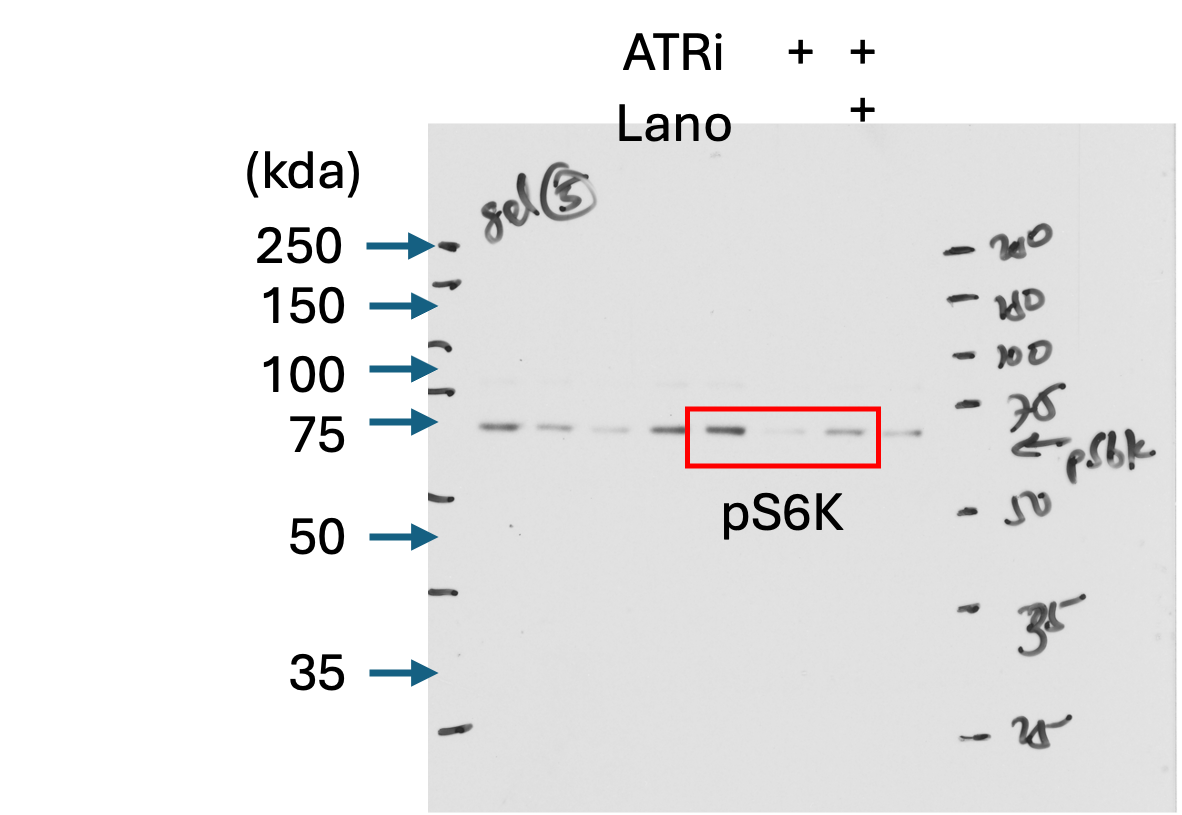

Supplement: Supplementary file 5 — Source data Fig. 4 [file 44319_2025_451_MOESM5_ESM.zip › Figure 4/Figure 4F/western pS6K.tif]

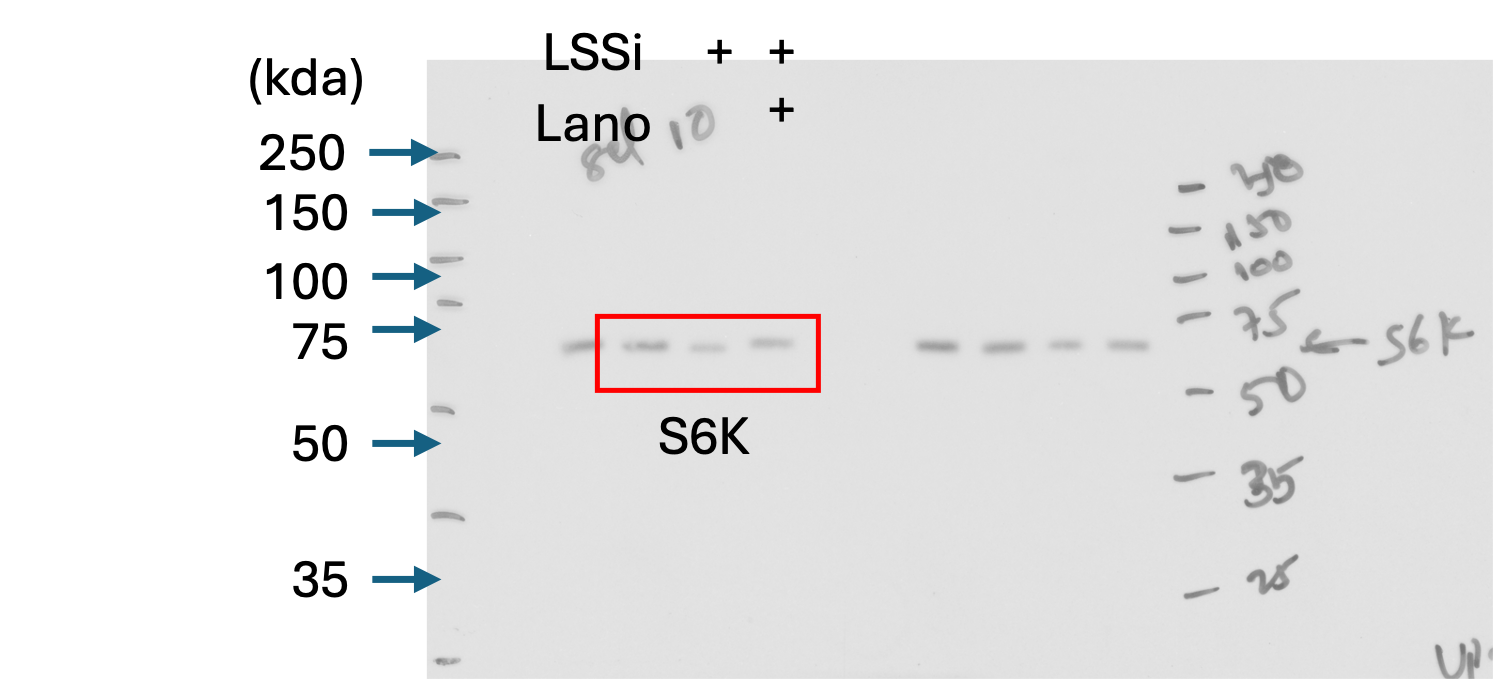

Supplement: Supplementary file 5 — Source data Fig. 4 [file 44319_2025_451_MOESM5_ESM.zip › Figure 4/Figure 4H/western S6K.tif]

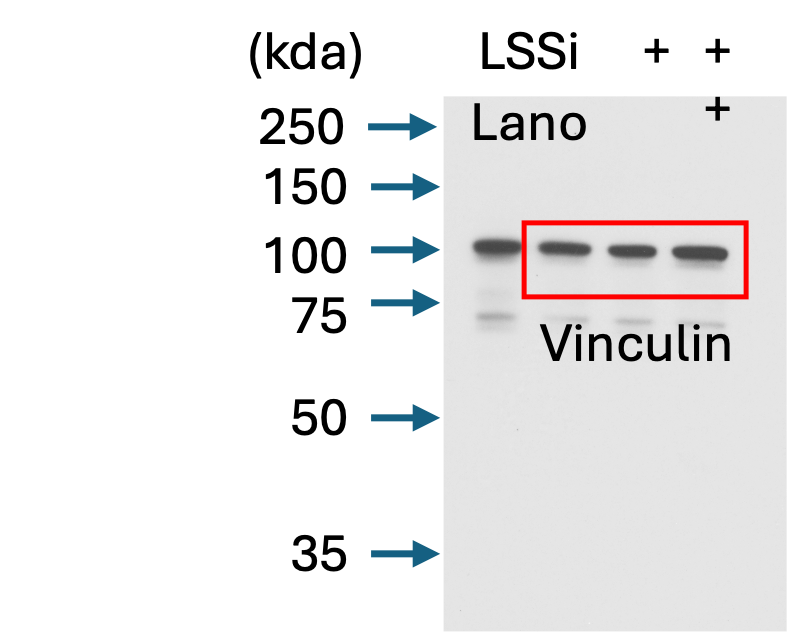

Supplement: Supplementary file 5 — Source data Fig. 4 [file 44319_2025_451_MOESM5_ESM.zip › Figure 4/Figure 4H/western vinculin.tif]

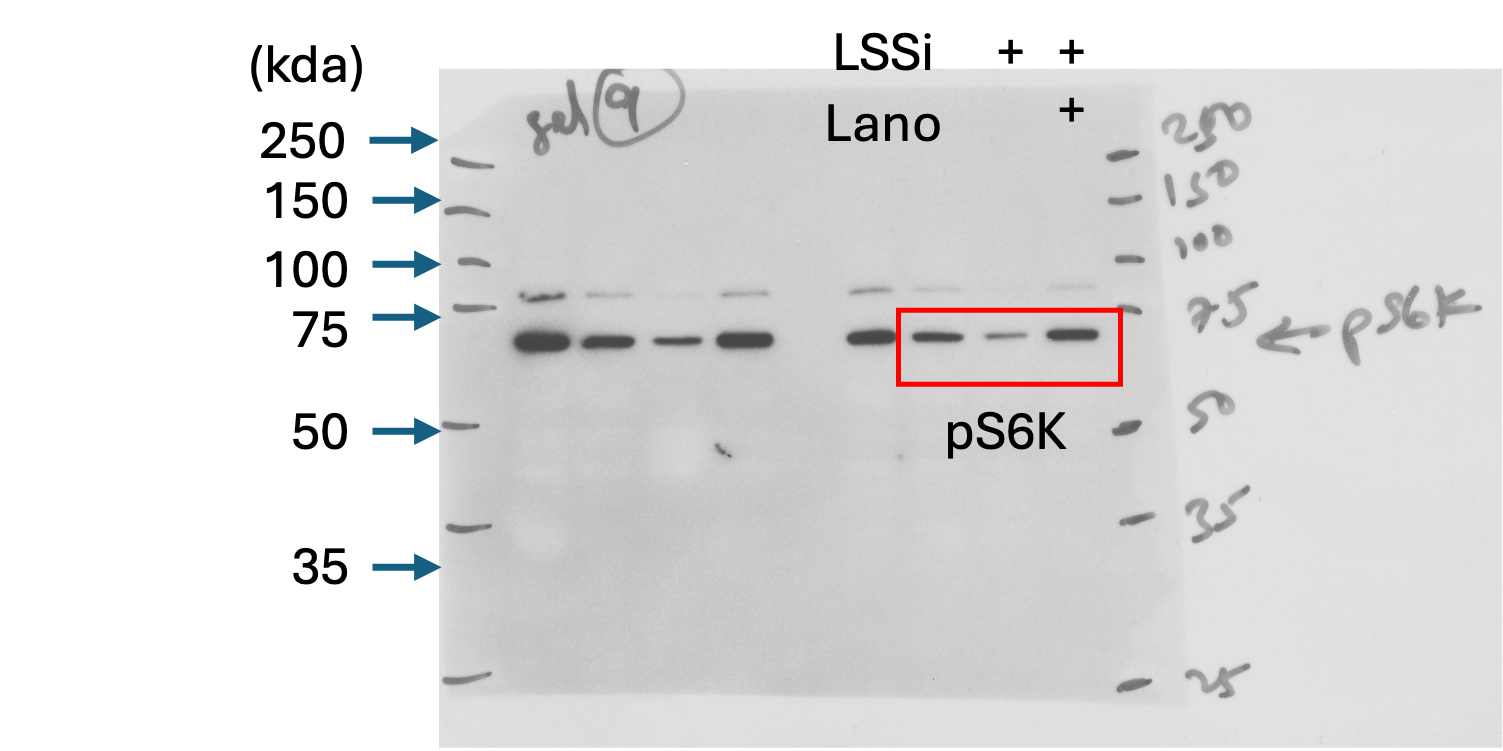

Supplement: Supplementary file 5 — Source data Fig. 4 [file 44319_2025_451_MOESM5_ESM.zip › Figure 4/Figure 4H/western pS6K.tif]

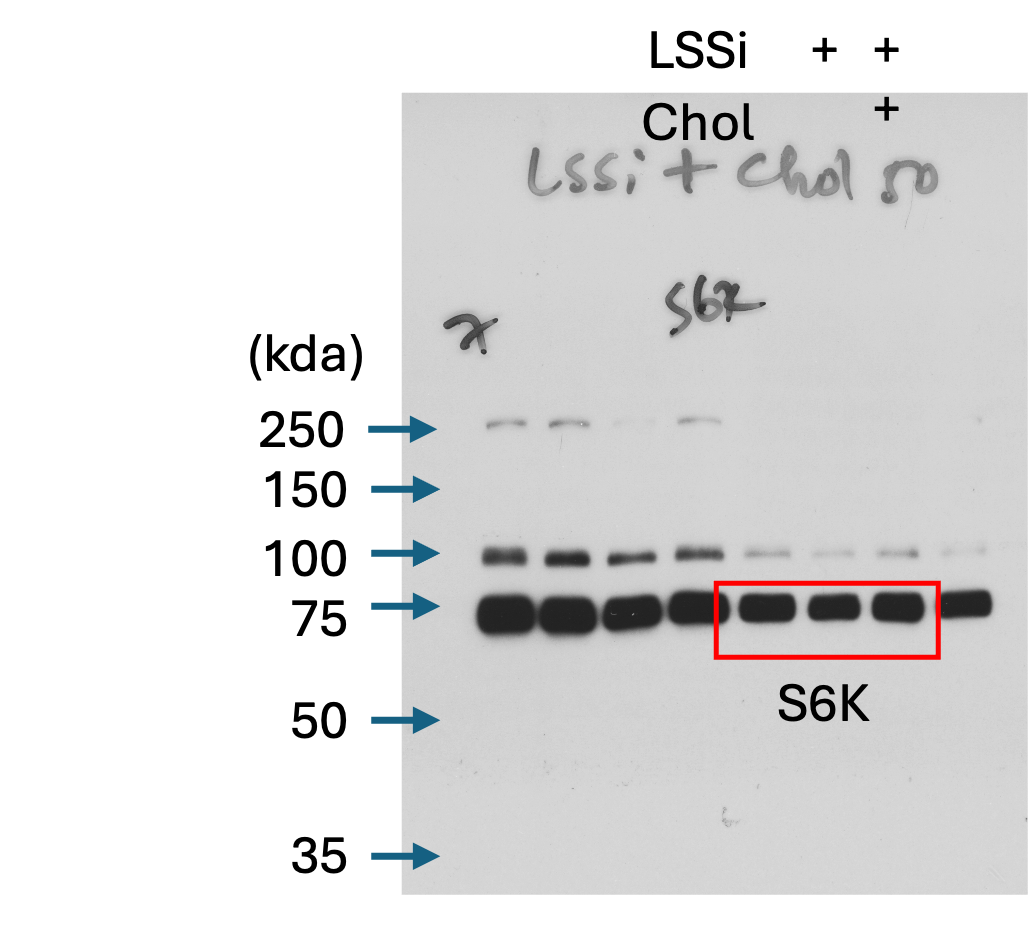

Supplement: Supplementary file 5 — Source data Fig. 4 [file 44319_2025_451_MOESM5_ESM.zip › Figure 4/Figure 4G/western S6K.tif]

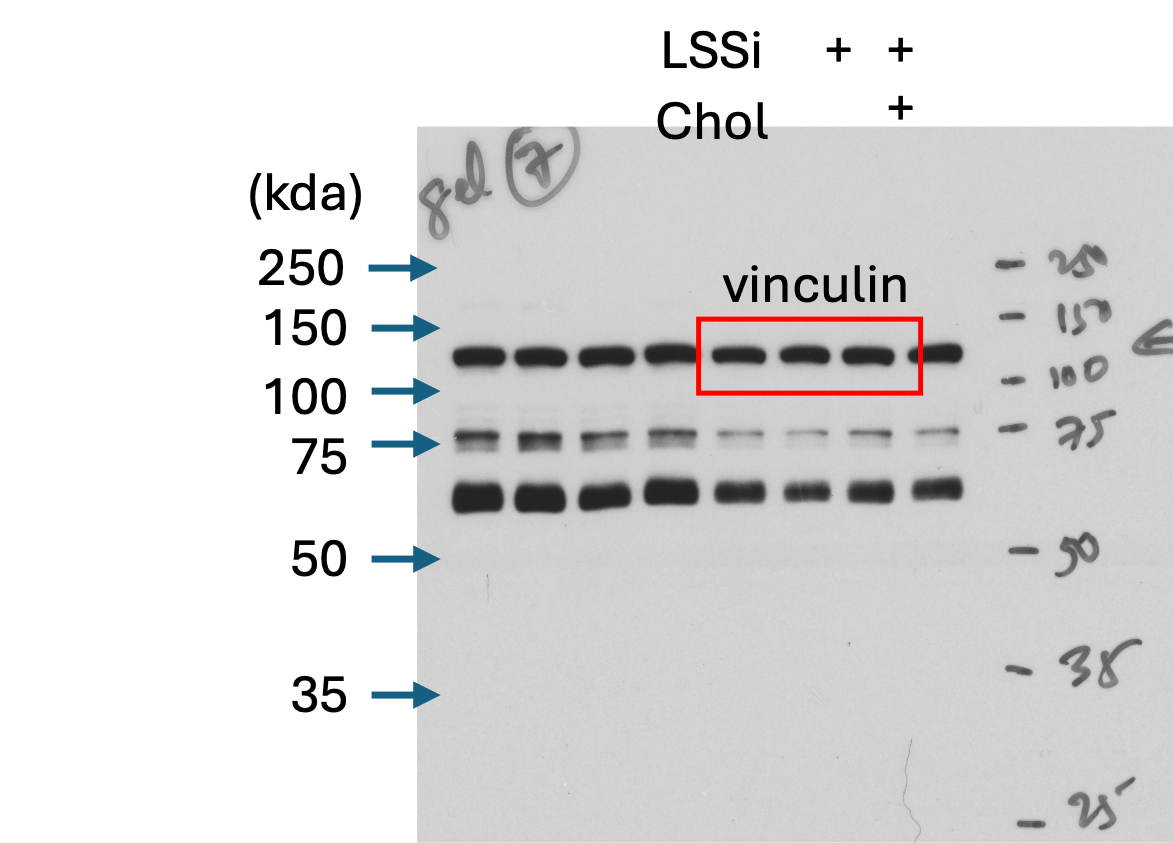

Supplement: Supplementary file 5 — Source data Fig. 4 [file 44319_2025_451_MOESM5_ESM.zip › Figure 4/Figure 4G/western vinculin.tif]

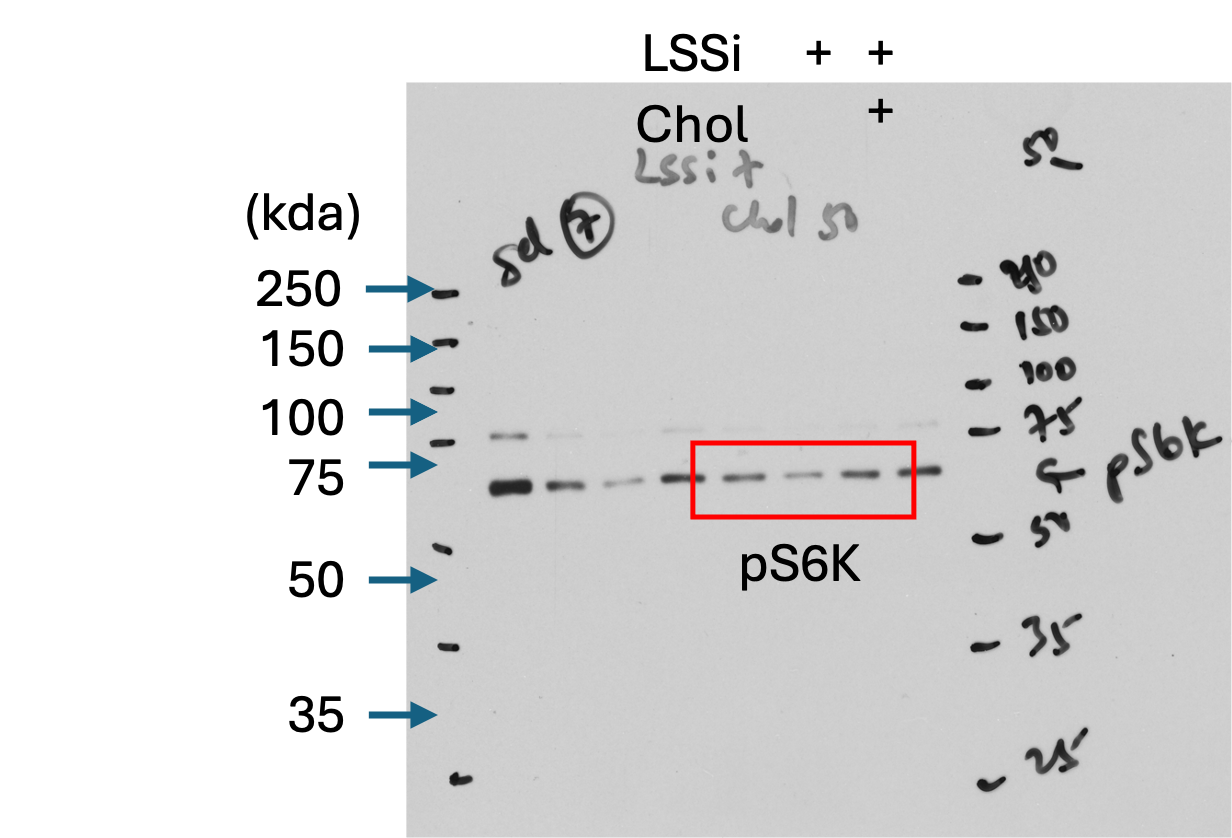

Supplement: Supplementary file 5 — Source data Fig. 4 [file 44319_2025_451_MOESM5_ESM.zip › Figure 4/Figure 4G/western pS6K.tif]

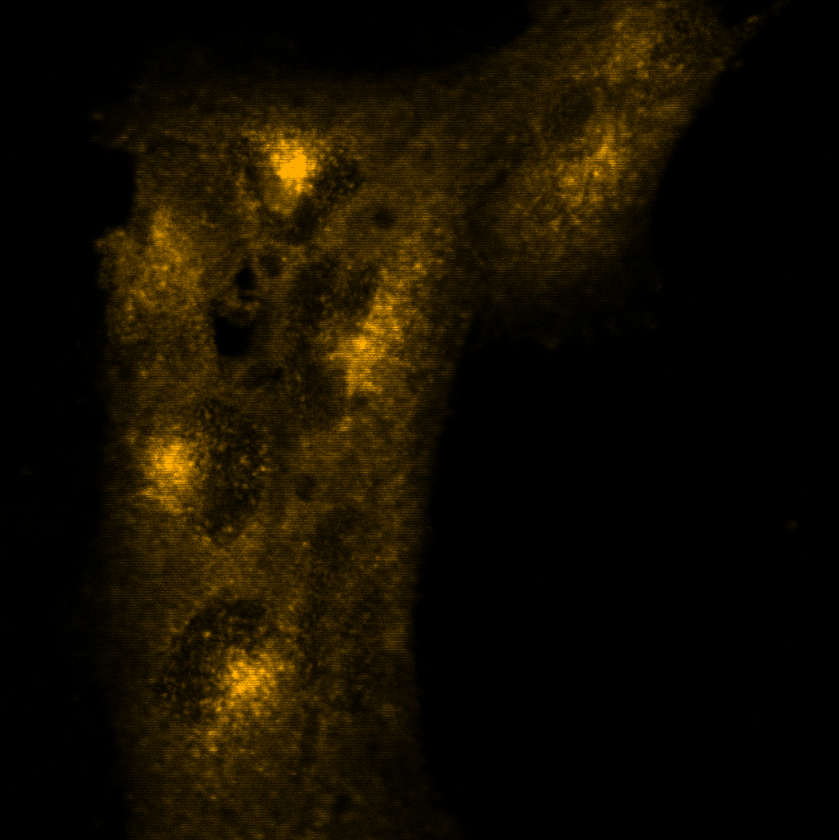

Supplement: Supplementary file 6 — Source data Fig. 5 [file 44319_2025_451_MOESM6_ESM.zip › Figure 5/Figure 5A/mTOR shp16 shATR cholesterol.tif]

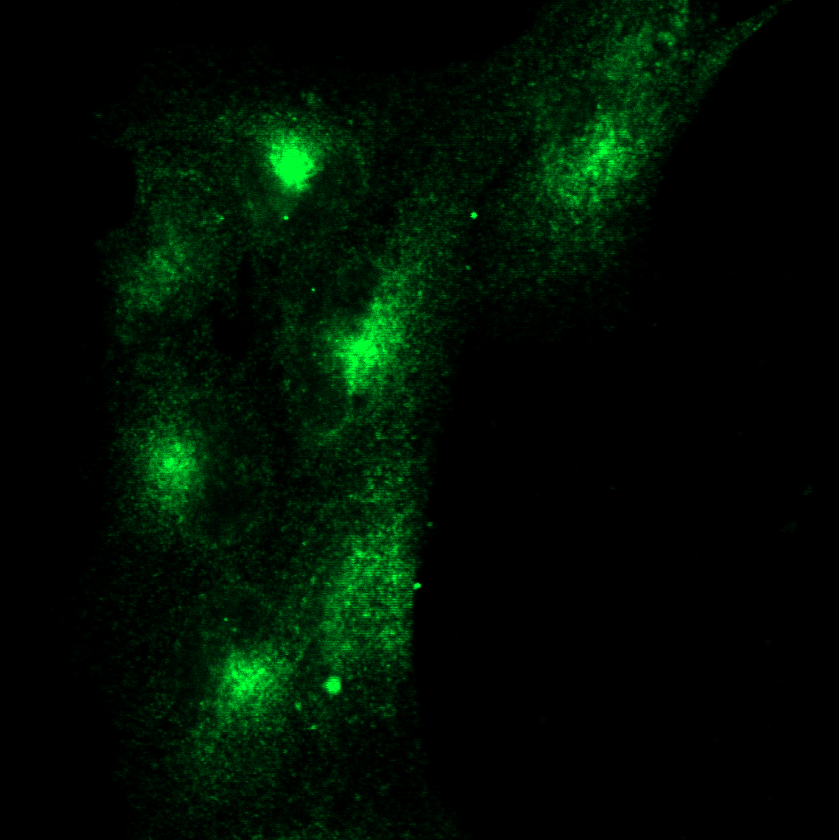

Supplement: Supplementary file 6 — Source data Fig. 5 [file 44319_2025_451_MOESM6_ESM.zip › Figure 5/Figure 5A/LAMP2 shp16 shATR cholesterol.tif]

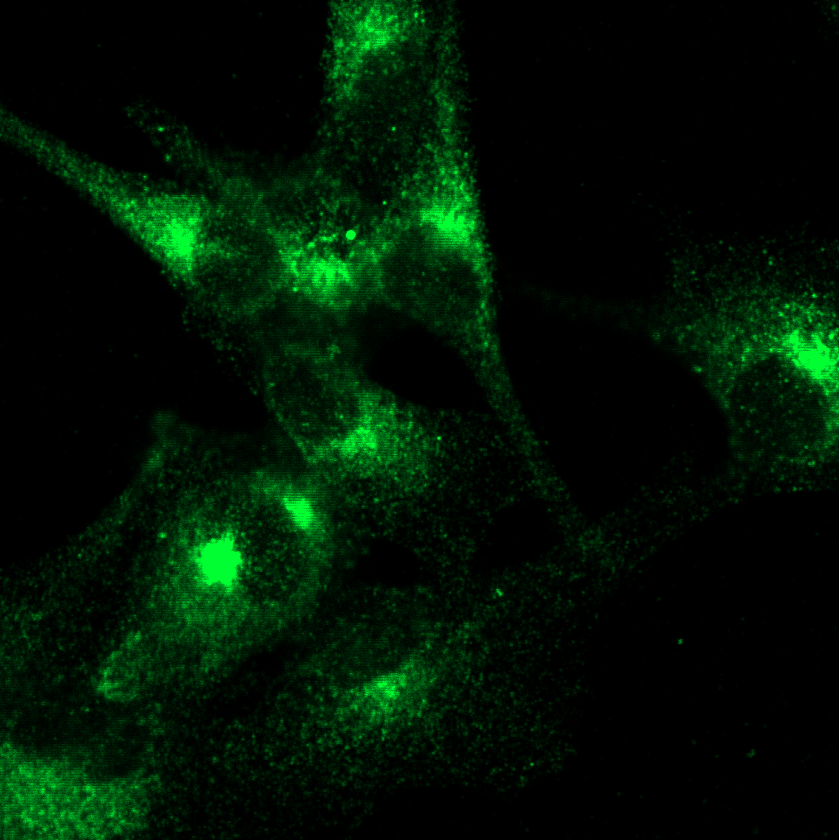

Supplement: Supplementary file 6 — Source data Fig. 5 [file 44319_2025_451_MOESM6_ESM.zip › Figure 5/Figure 5A/LAMP2 shp16 shCont cholesterol.tif]

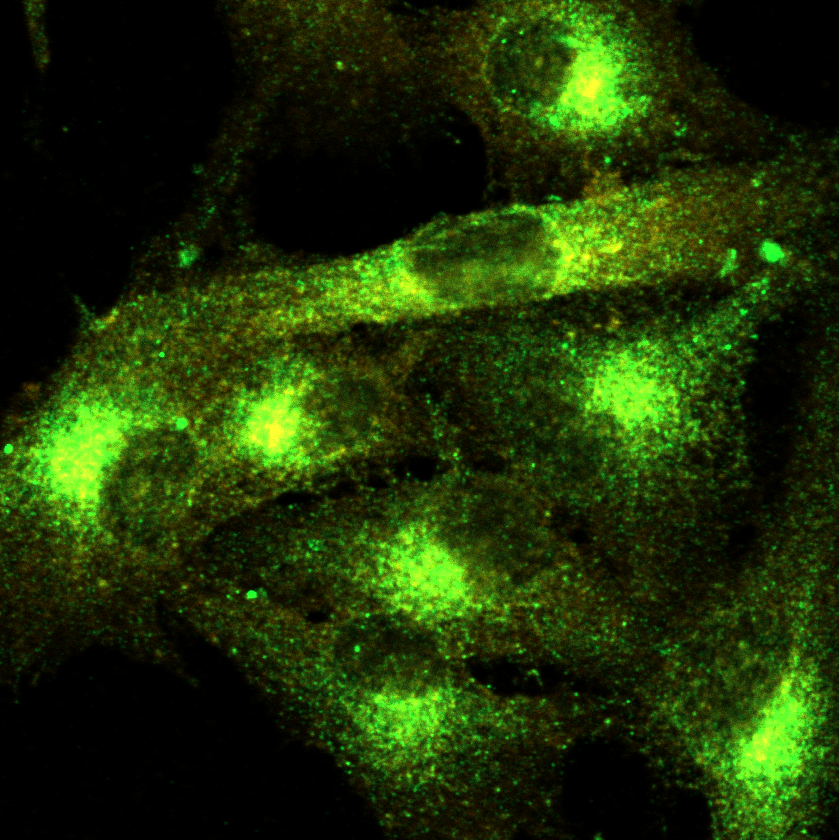

Supplement: Supplementary file 6 — Source data Fig. 5 [file 44319_2025_451_MOESM6_ESM.zip › Figure 5/Figure 5A/Merge shp16 shCont.tif]

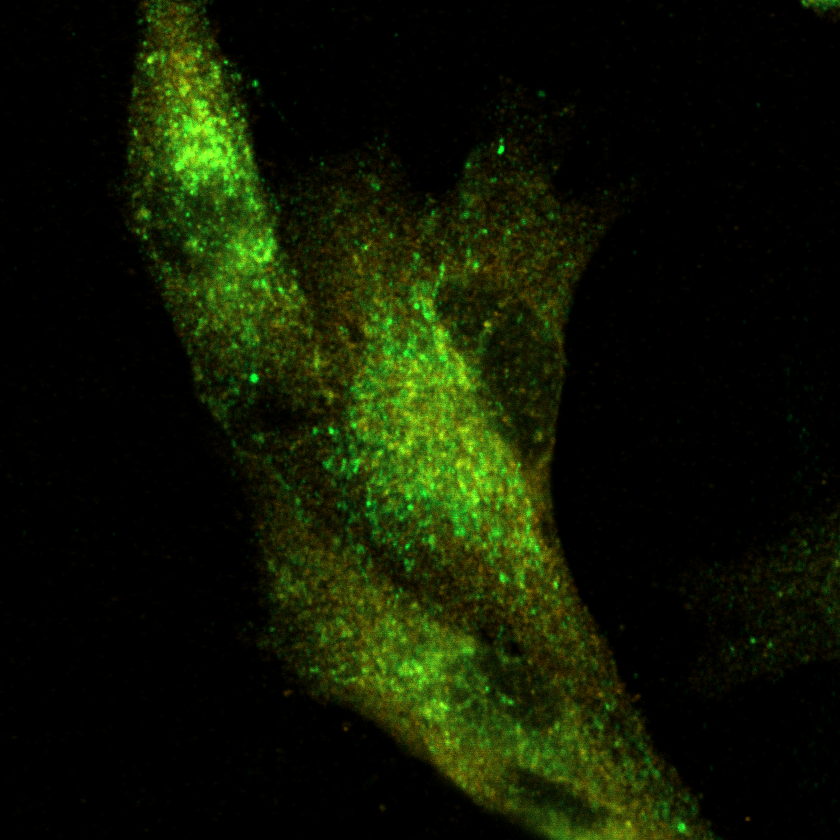

Supplement: Supplementary file 6 — Source data Fig. 5 [file 44319_2025_451_MOESM6_ESM.zip › Figure 5/Figure 5A/Merge shp16 shATR.tif]

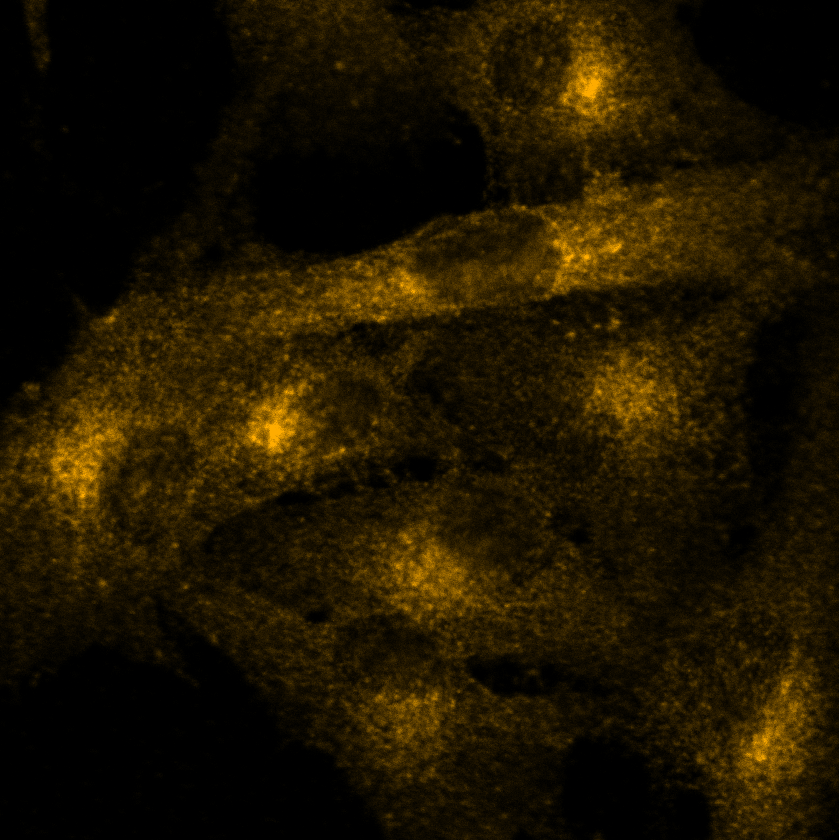

Supplement: Supplementary file 6 — Source data Fig. 5 [file 44319_2025_451_MOESM6_ESM.zip › Figure 5/Figure 5A/mTOR shp16 shCont.tif]

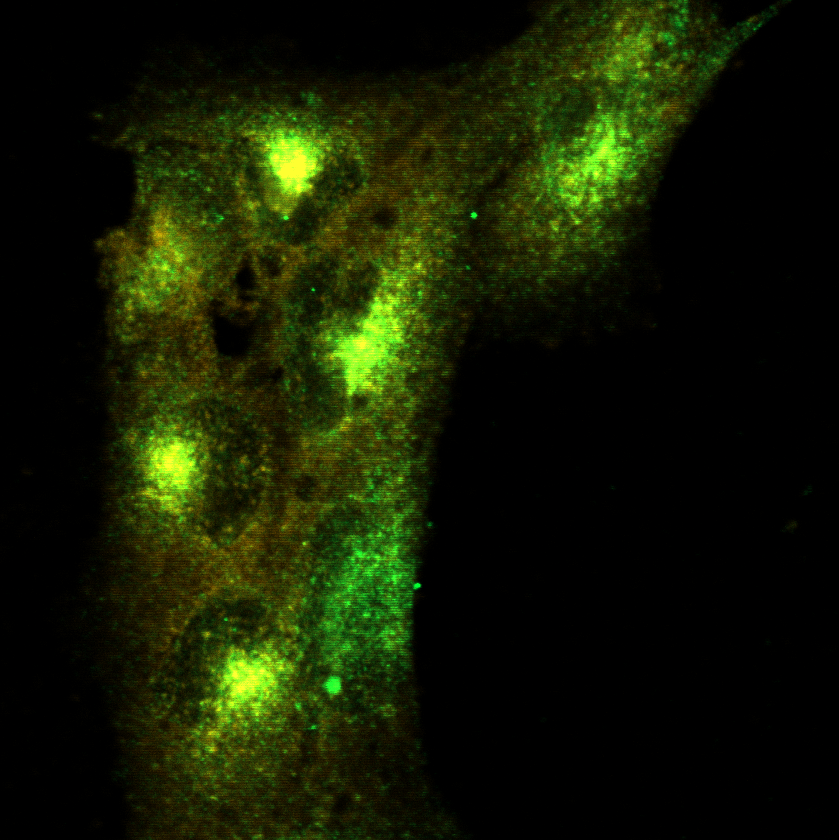

Supplement: Supplementary file 6 — Source data Fig. 5 [file 44319_2025_451_MOESM6_ESM.zip › Figure 5/Figure 5A/Merge shp16 shATR cholesterol.tif]

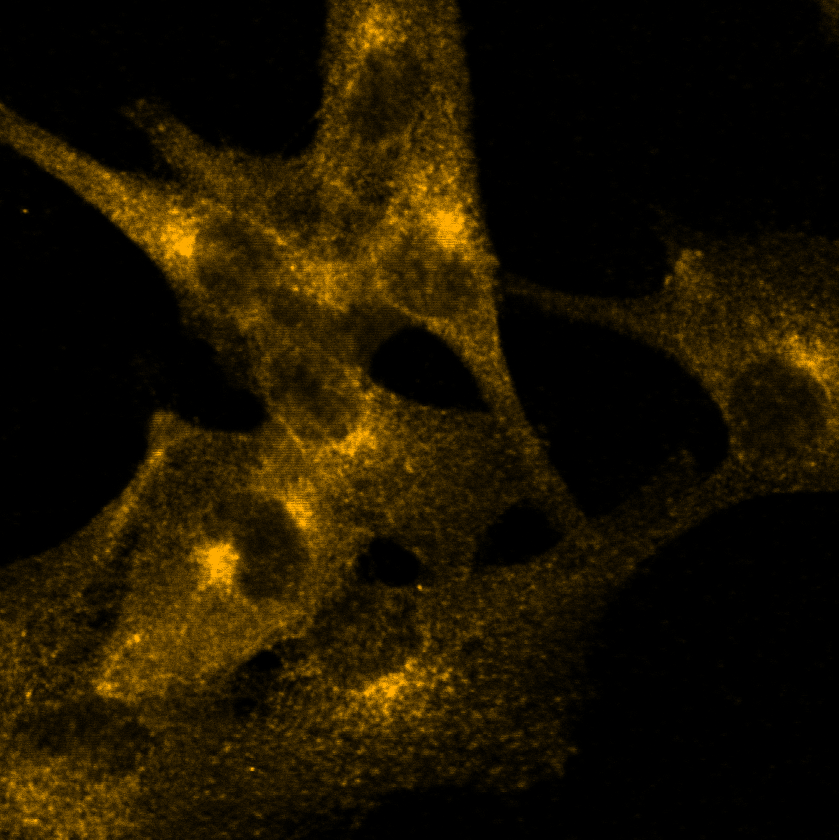

Supplement: Supplementary file 6 — Source data Fig. 5 [file 44319_2025_451_MOESM6_ESM.zip › Figure 5/Figure 5A/mTOR shp16 shCont cholesterol.tif]

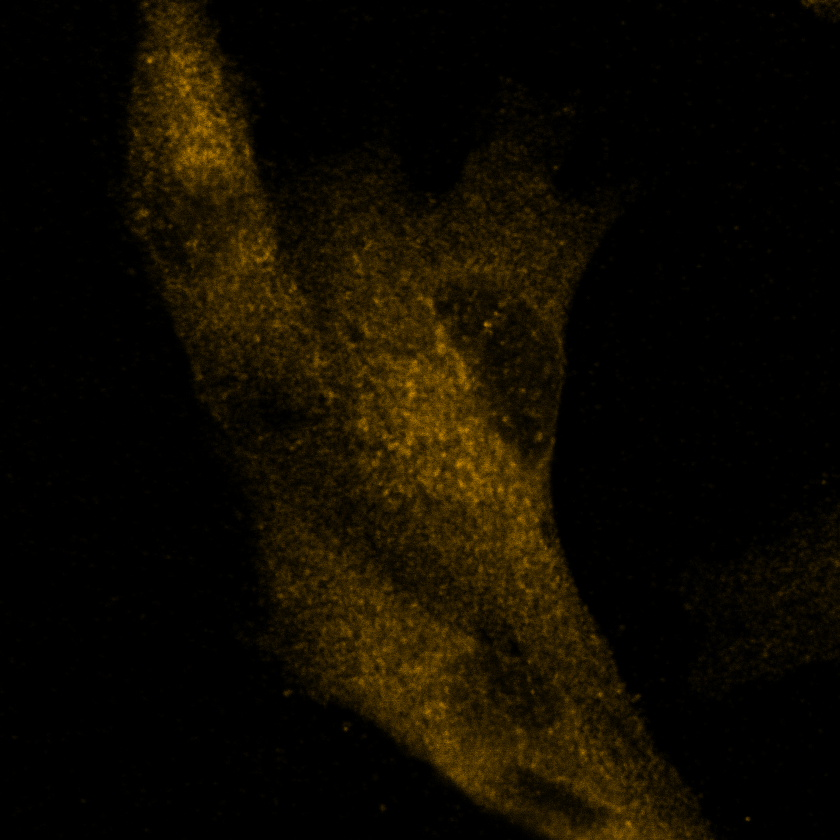

Supplement: Supplementary file 6 — Source data Fig. 5 [file 44319_2025_451_MOESM6_ESM.zip › Figure 5/Figure 5A/mTOR shp16 shATR.tif]

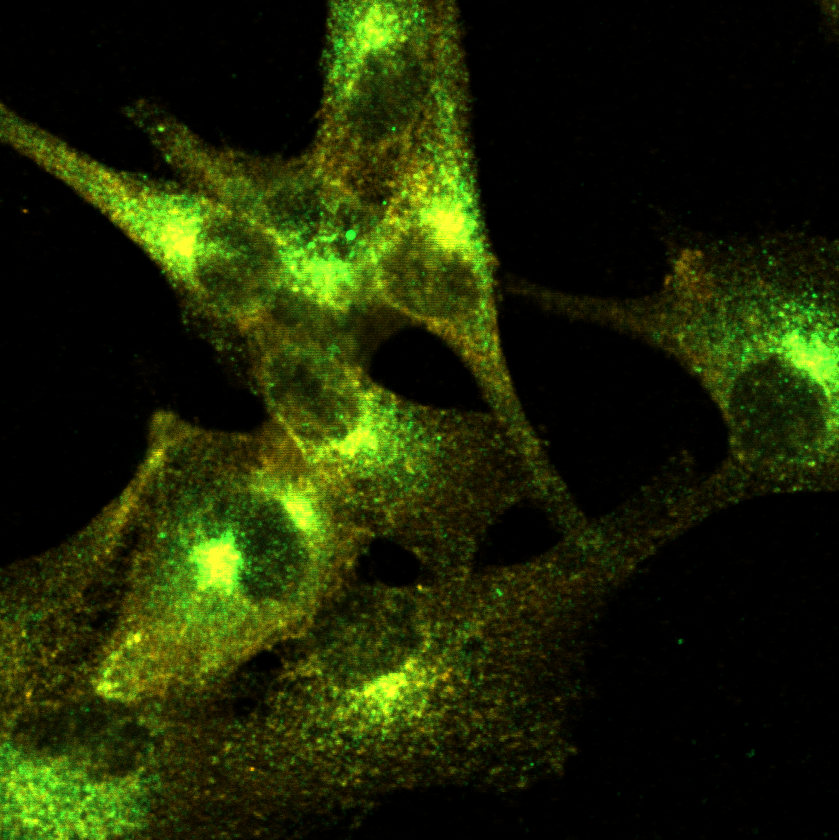

Supplement: Supplementary file 6 — Source data Fig. 5 [file 44319_2025_451_MOESM6_ESM.zip › Figure 5/Figure 5A/Merge shp16 shCont cholesterol.tif]

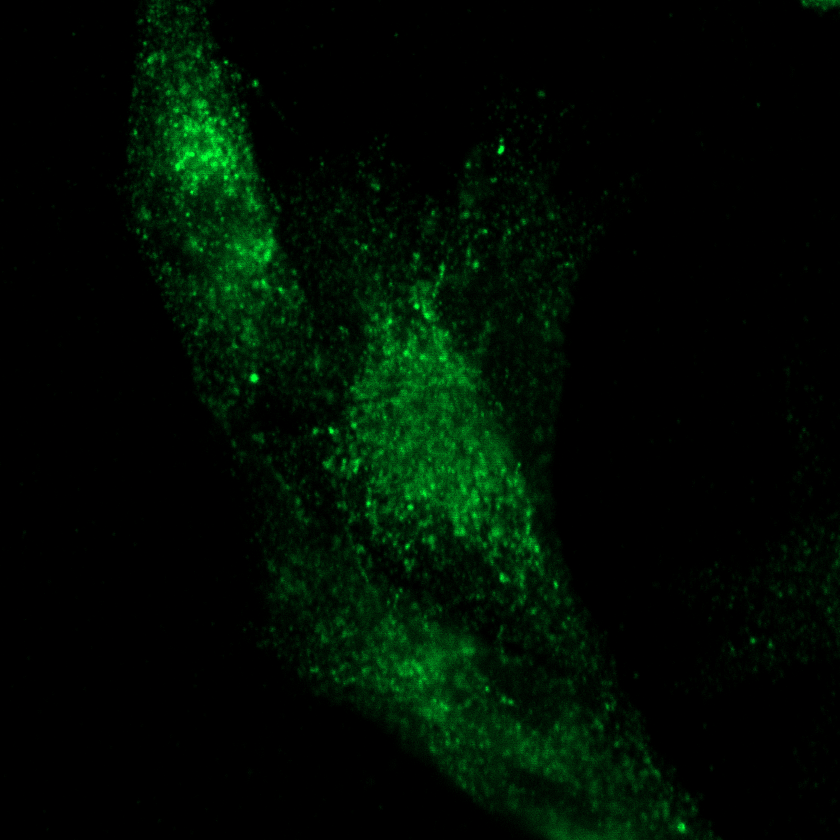

Supplement: Supplementary file 6 — Source data Fig. 5 [file 44319_2025_451_MOESM6_ESM.zip › Figure 5/Figure 5A/LAMP2 shp16 shATR.tif]

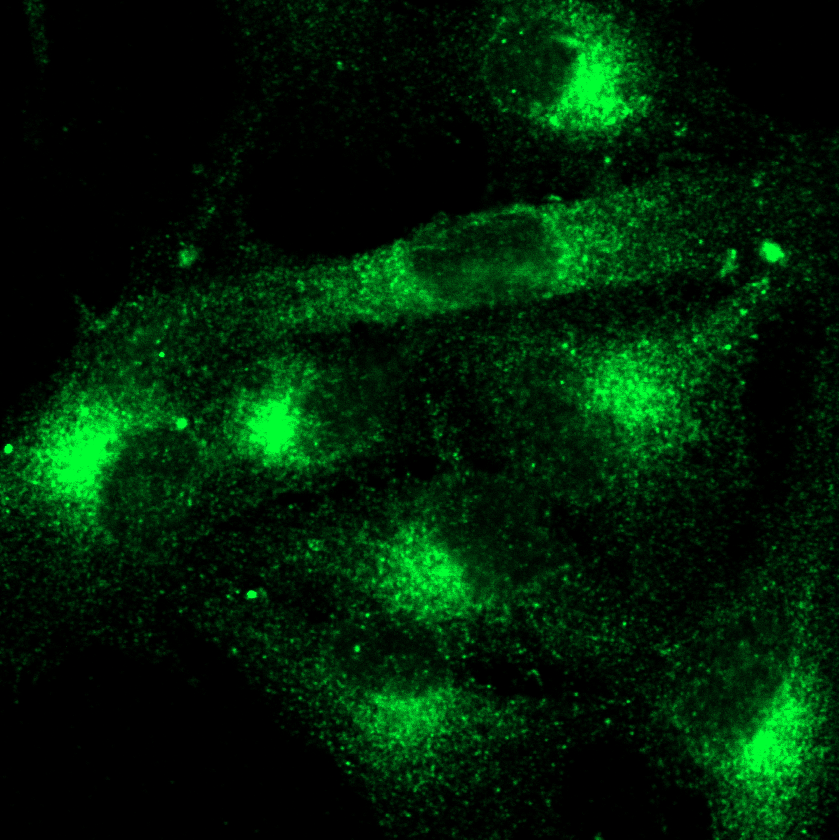

Supplement: Supplementary file 6 — Source data Fig. 5 [file 44319_2025_451_MOESM6_ESM.zip › Figure 5/Figure 5A/LAMP2 shp16 shCont.tif]

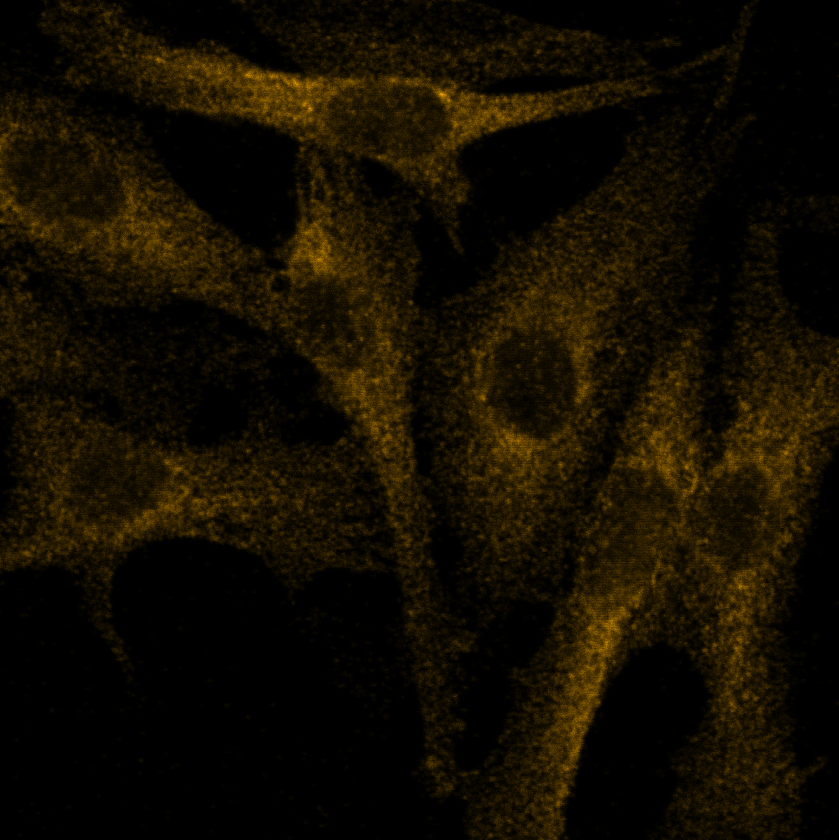

Supplement: Supplementary file 6 — Source data Fig. 5 [file 44319_2025_451_MOESM6_ESM.zip › Figure 5/Figure 5B/TSC2 shp16 shCont Cholesterol.tif]

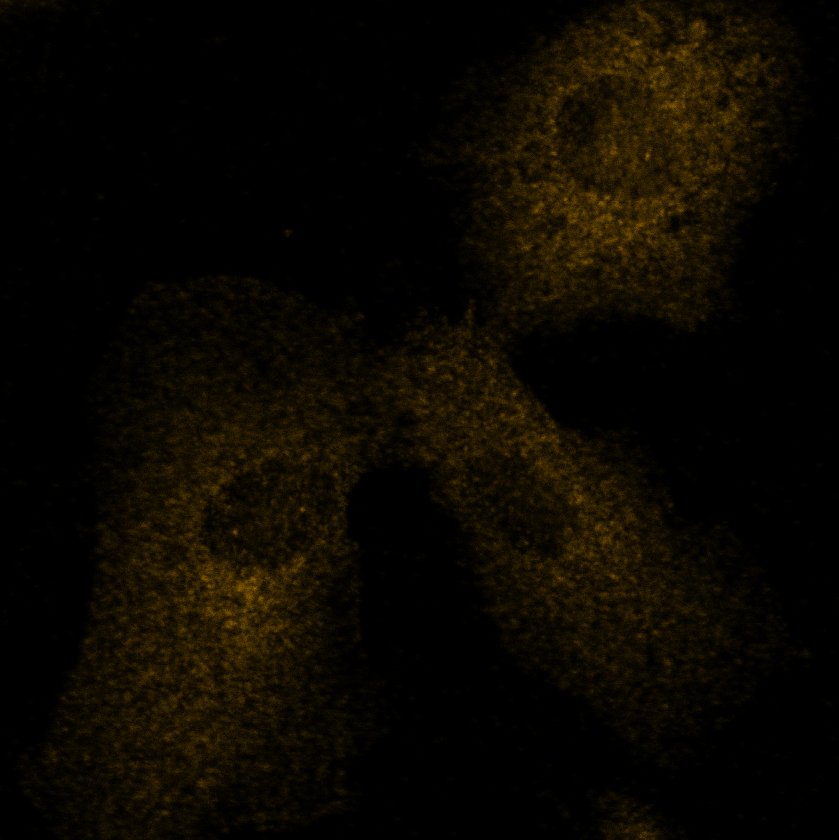

Supplement: Supplementary file 6 — Source data Fig. 5 [file 44319_2025_451_MOESM6_ESM.zip › Figure 5/Figure 5B/TSC2 shp16 shATR Cholesterol.tif]

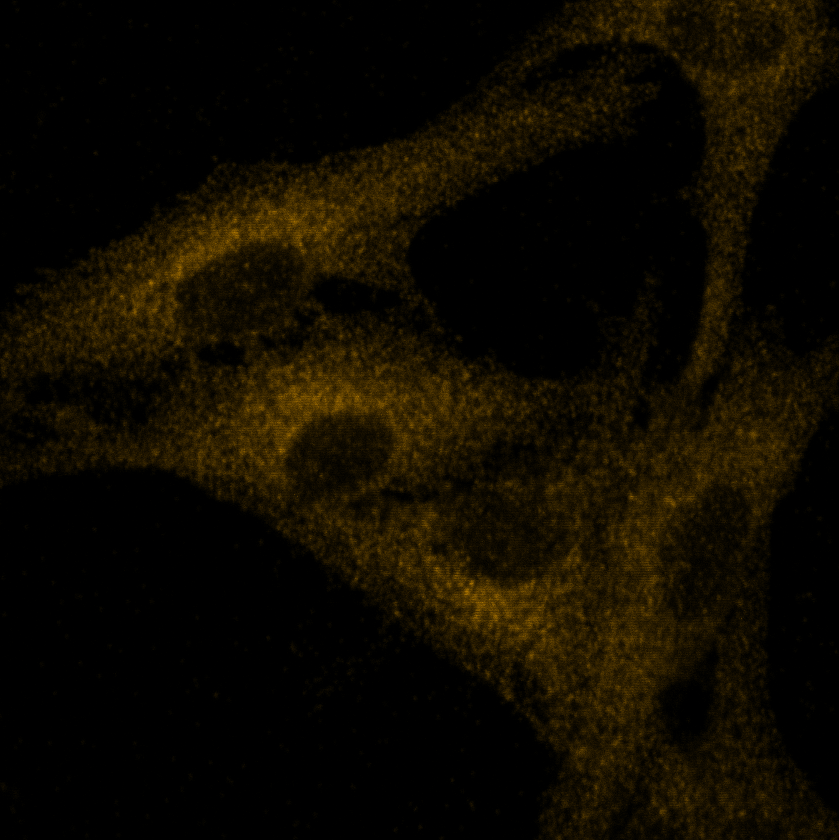

Supplement: Supplementary file 6 — Source data Fig. 5 [file 44319_2025_451_MOESM6_ESM.zip › Figure 5/Figure 5B/TSC2 shp16 shCont.tif]

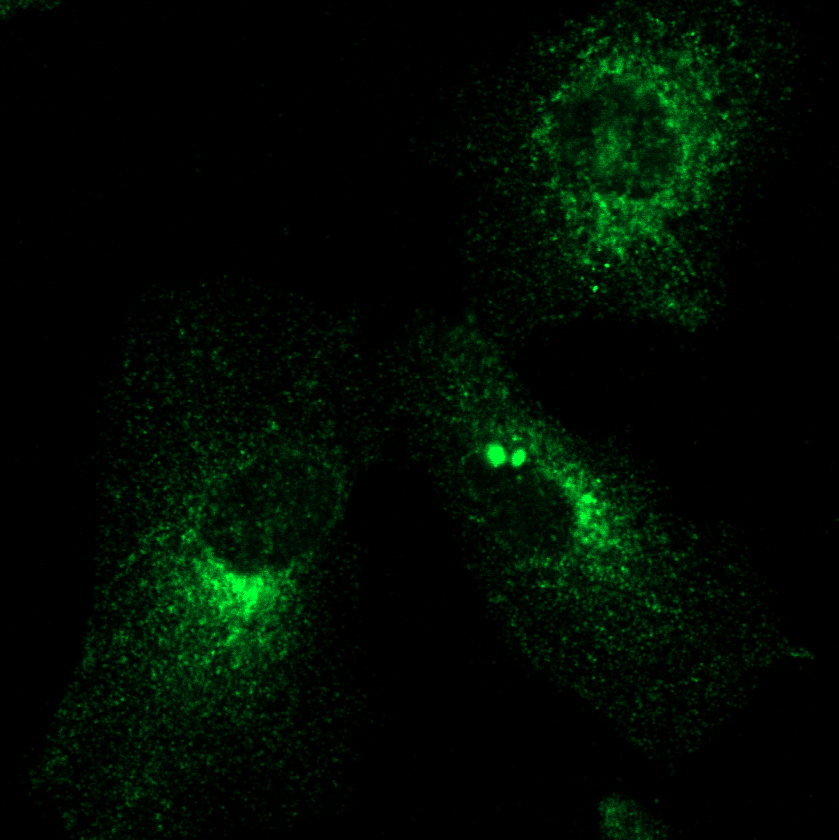

Supplement: Supplementary file 6 — Source data Fig. 5 [file 44319_2025_451_MOESM6_ESM.zip › Figure 5/Figure 5B/LAMP2 shp16 shATR Cholesterol.tif]

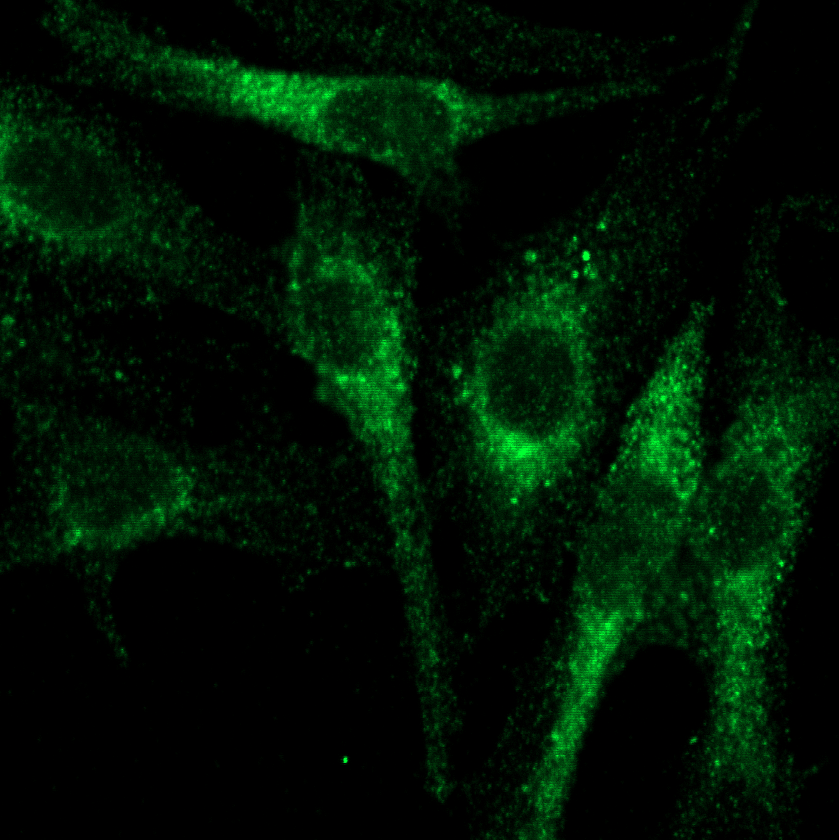

Supplement: Supplementary file 6 — Source data Fig. 5 [file 44319_2025_451_MOESM6_ESM.zip › Figure 5/Figure 5B/LAMP2 shp16 shCont Cholesterol.tif]

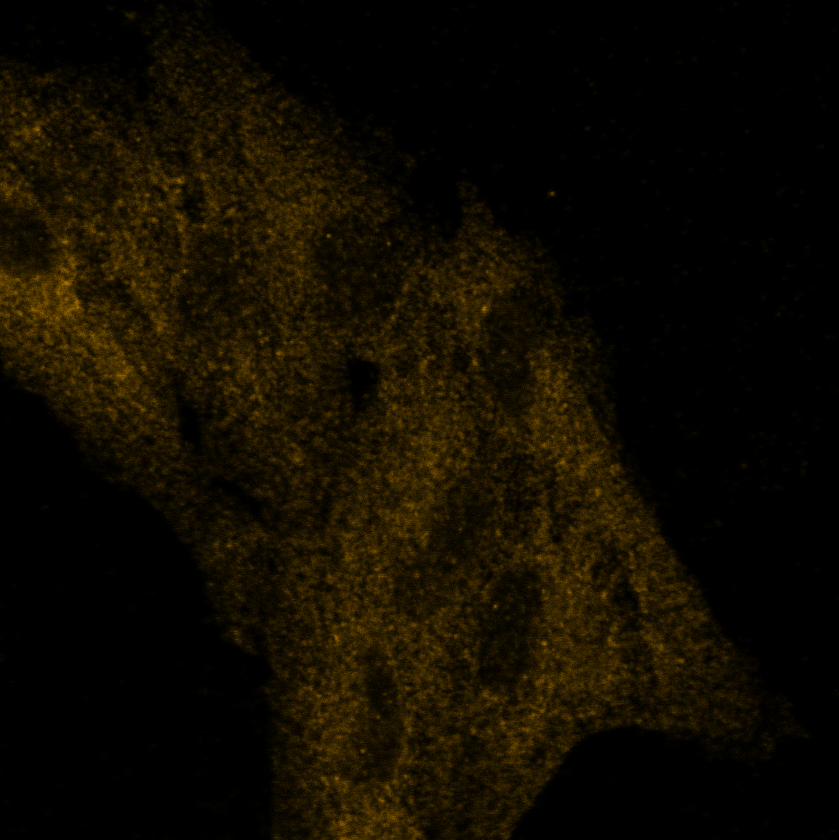

Supplement: Supplementary file 6 — Source data Fig. 5 [file 44319_2025_451_MOESM6_ESM.zip › Figure 5/Figure 5B/TSC2 shp16 shATR.tif]

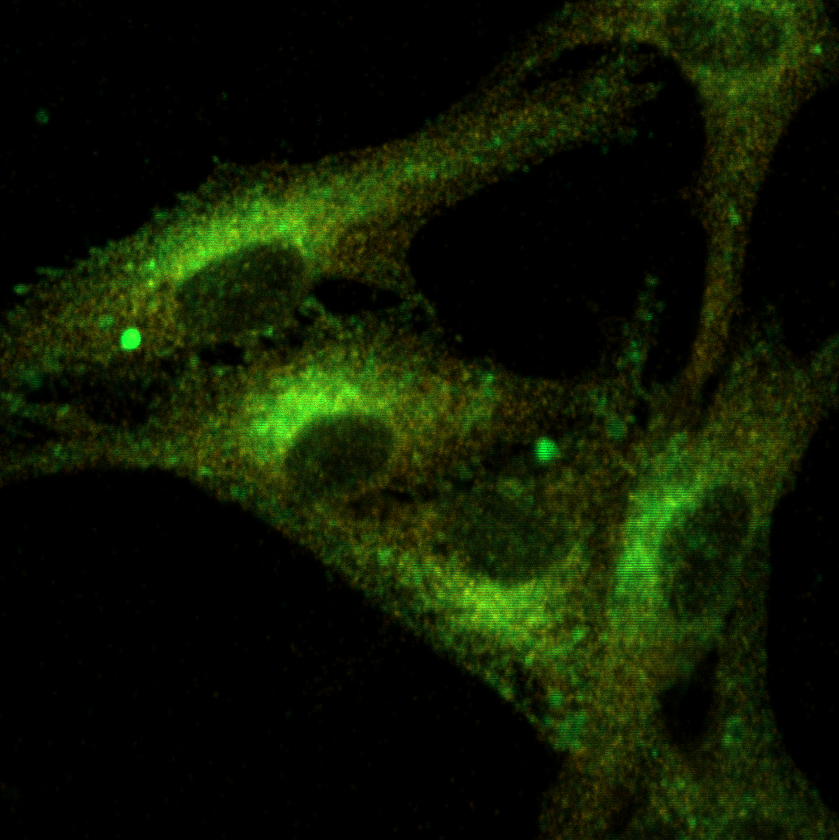

Supplement: Supplementary file 6 — Source data Fig. 5 [file 44319_2025_451_MOESM6_ESM.zip › Figure 5/Figure 5B/Merge shp16 shCont.tif]

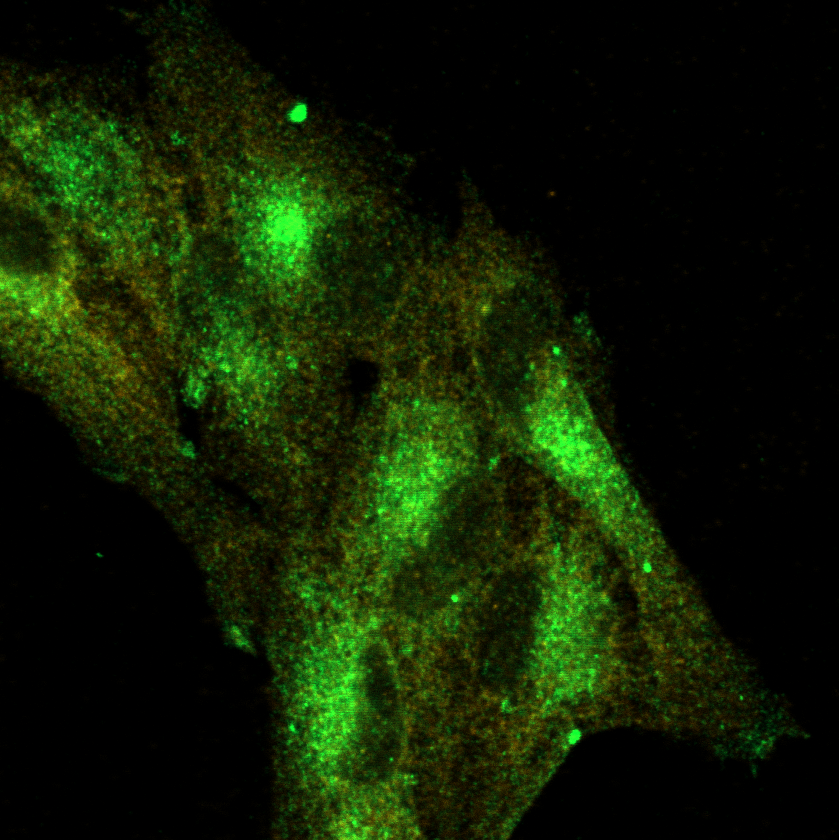

Supplement: Supplementary file 6 — Source data Fig. 5 [file 44319_2025_451_MOESM6_ESM.zip › Figure 5/Figure 5B/Merge shp16 shATR.tif]

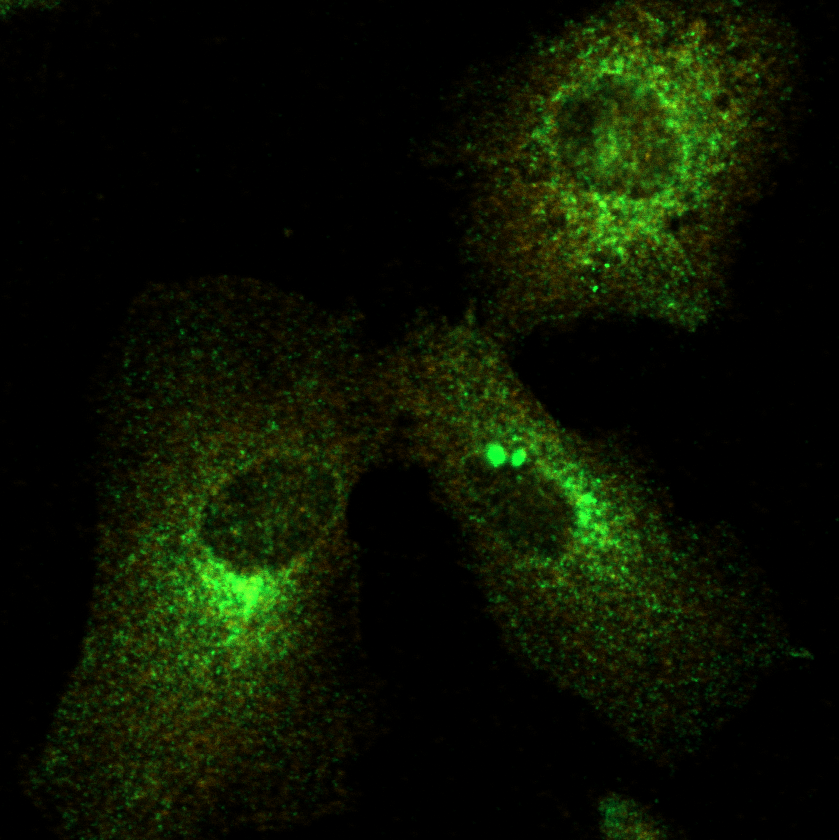

Supplement: Supplementary file 6 — Source data Fig. 5 [file 44319_2025_451_MOESM6_ESM.zip › Figure 5/Figure 5B/Merge shp16 shATR Cholesterol.tif]

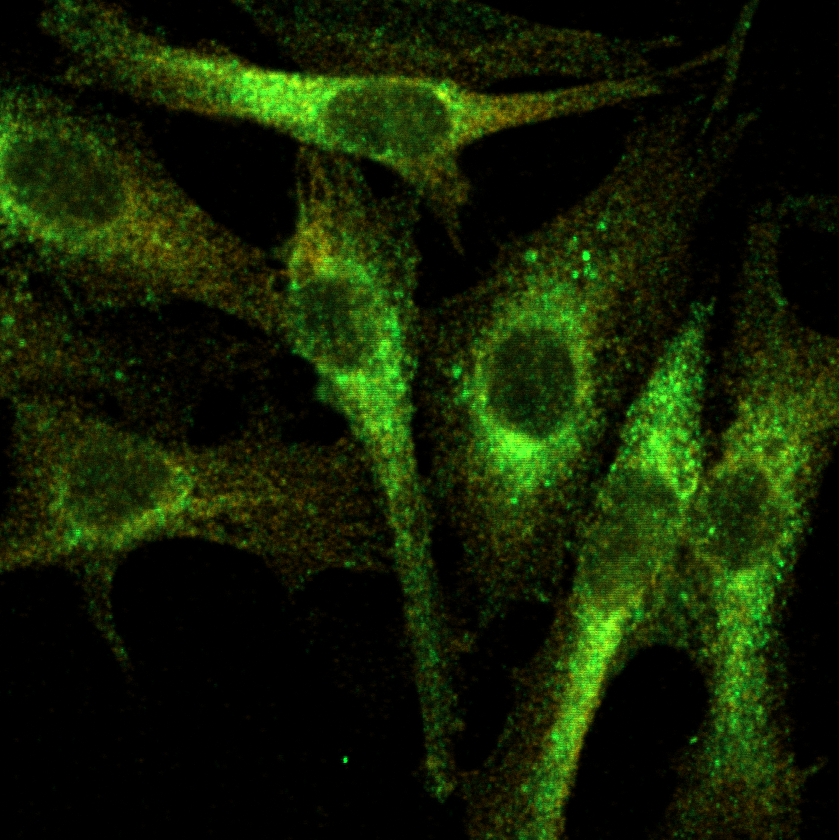

Supplement: Supplementary file 6 — Source data Fig. 5 [file 44319_2025_451_MOESM6_ESM.zip › Figure 5/Figure 5B/Merge shp16 shCont Cholesterol.tif]

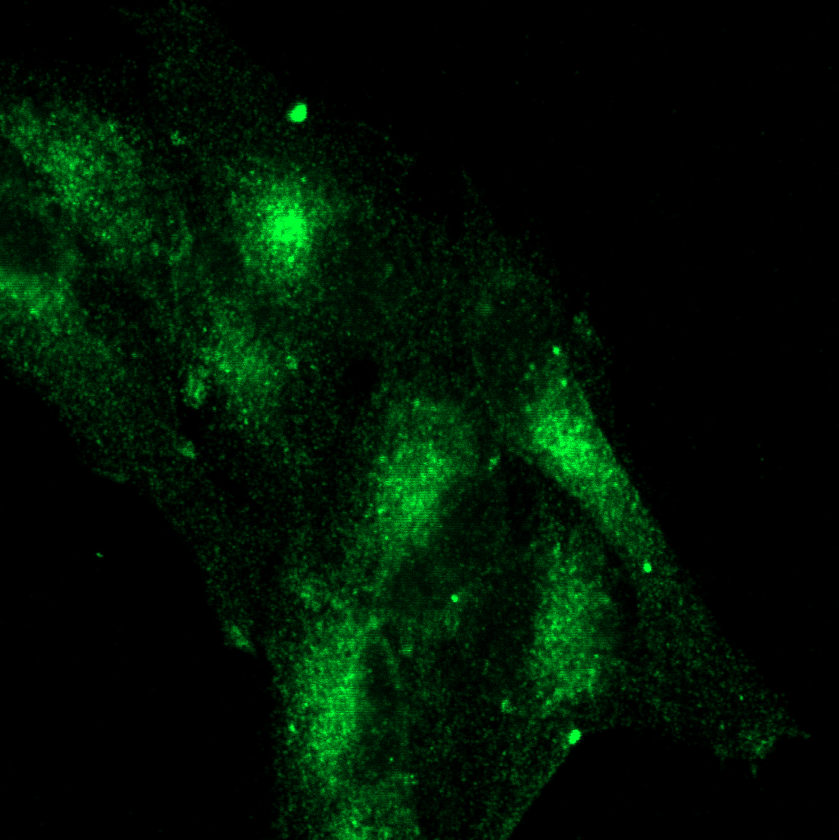

Supplement: Supplementary file 6 — Source data Fig. 5 [file 44319_2025_451_MOESM6_ESM.zip › Figure 5/Figure 5B/LAMP2 shp16 shATR.tif]

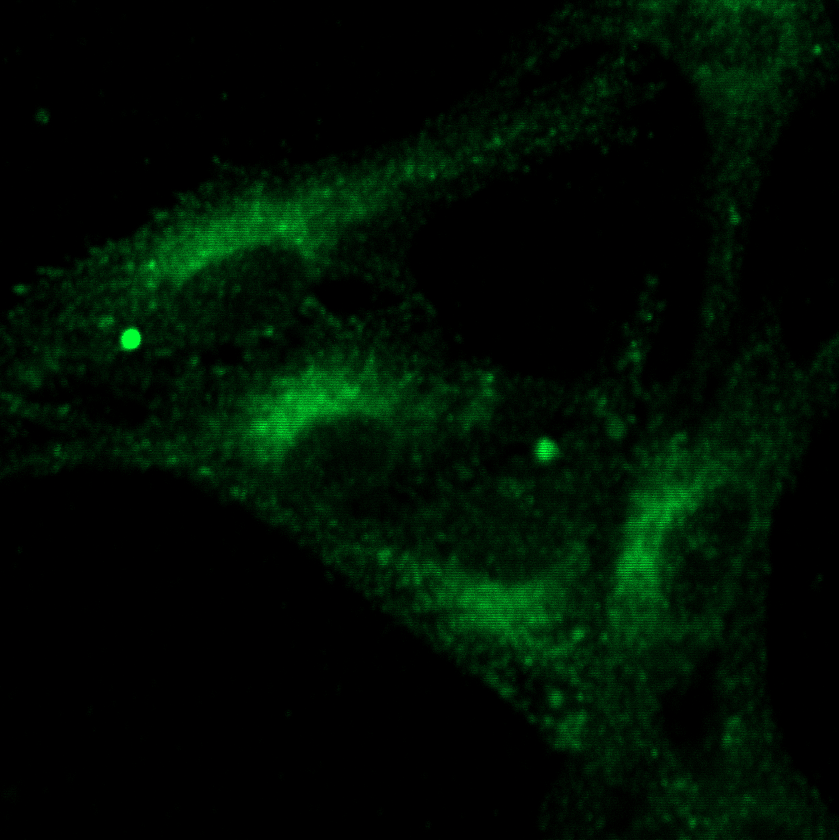

Supplement: Supplementary file 6 — Source data Fig. 5 [file 44319_2025_451_MOESM6_ESM.zip › Figure 5/Figure 5B/LAMP2 shp16 shCont.tif]
