# Supplementary material for: ATR promotes mTORC1 activity via de novo cholesterol synthesis
Source: EMBO Rep. 2025 Jun 13;26(14):3574–93. doi: 10.1038/s44319-025-00451-3 (PMC12287318; doi:10.1038/s44319-025-00451-3)
Supplement: Supplementary file 7 — Expanded View Figures [file 44319_2025_451_MOESM7_ESM.pdf]

# Expanded View Figures

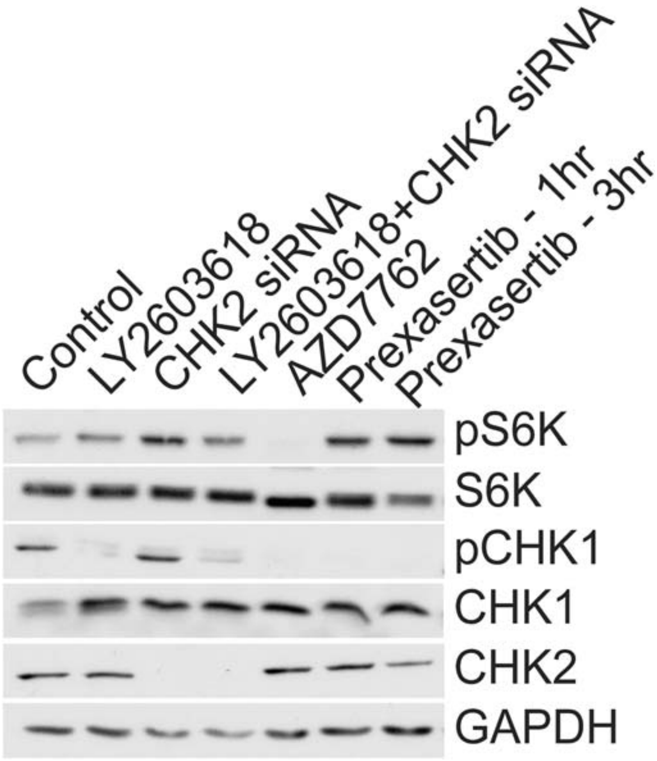


**Figure EV1. Suppression of CHK1 and CHK2 do not markedly decrease mTORC1 activity.**

HeLa cells were treated with the CHK1 inhibitor LY2603618 (2 μM, 3 h), CHK2 siRNA (50 nM, 48 h), the dual CHK1/CHK2 inhibitor AZD7762 (2 μM, 3 h), or the dual CHK1/CHK2 inhibitor Prexasertib [100 nM, 1 (lane 6) or 3 (lane 7) hrs], and the indicated proteins were assessed by western blotting. GAPDH was used as a loading control. Data information: Western blot data are representative of data from at least three independent experiments.

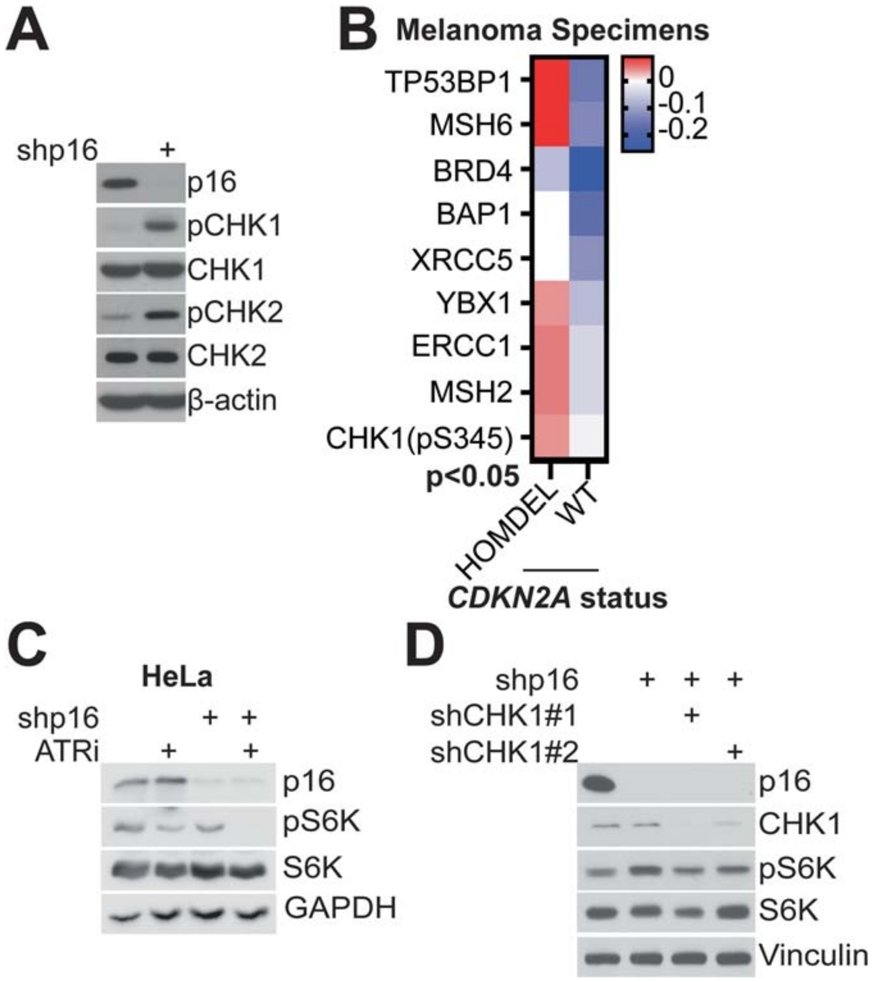


**Figure EV2. p16 knockdown increases activation of the ATR/ATM pathways; ATR-mediated mTORC1 activation in p16 knockdown cells is independent of CHK1.**

(**A**) SKMEL28 cells were transduced with a lentivirus expressing a short hairpin targeting GFP as a control or p16 (shp16), and the indicated proteins were assessed by western blotting. β-actin was used as loading controls. (**B**) RPPA results from Melanoma patient samples show upregulation of proteins related to the DNA damage response and repair in tumors with homozygous deletion (HOMDEL) of *CDKN2A* (encoding p16) compared to wildtype (WT) *CDKN2A* (all proteins $p < 0.05$). (**C**) HeLa cells were transduced with a lentivirus expressing a short hairpin targeting GFP as a control or p16 (shp16) and treated with 0.5 μM AZD6738 (ATRi) for 30 min. The indicated proteins were assessed by western blotting. GAPDH was used as a loading control. (**D**) Same as (**A**), but shp16 cells were transduced with a lentivirus expressing a short hairpin targeting GFP or two short hairpins targeting CHK1 (shCHK1 #1 and #2), and the indicated proteins were assessed by western blotting. Vinculin was used as a loading control. Data information: Western blot data in (**A**, **C**, **D**) are representative data from at least three independent experiments.

 © The Author(s)

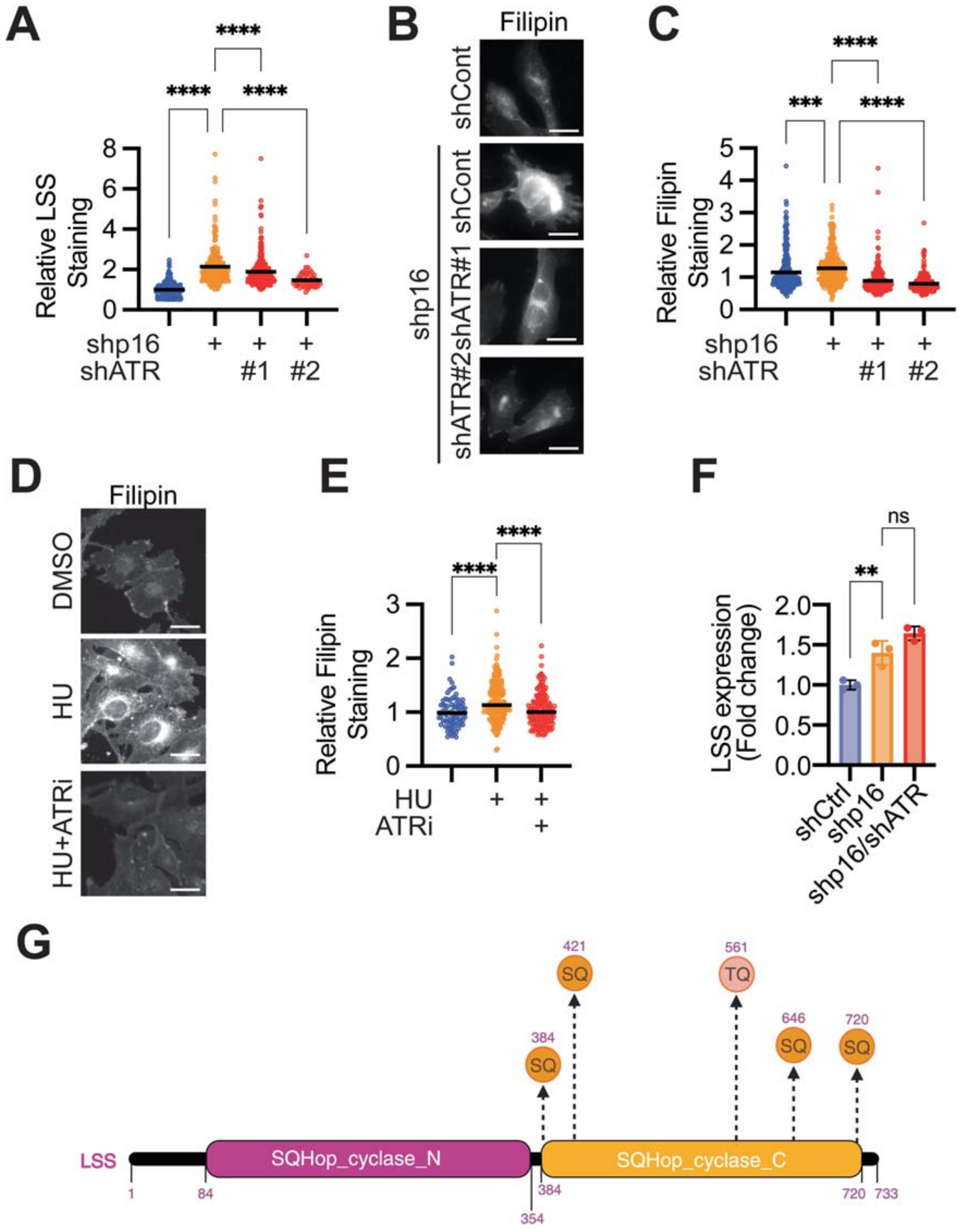
A
****
****
****
Relative LSS Staining
8
6
4
2
0
shp16 + + +
shATR #1 #2
B
Filipin
shCont
shp16
shCont
shATR#1
shATR#2
C
****
***
****
Relative Filipin Staining
5
4
3
2
1
0
shp16 + + +
shATR #1 #2
D
Filipin
DMSO
HU
HU+ATRi
E
****
****
Relative Filipin Staining
3
2
1
0
HU + +
ATRi +
F
ns
**
LSS expression (Fold change)
2.0
1.5
1.0
0.5
0.0
shCtrl
shp16
shp16/shATR
G
421
SQ
561
TQ
384
SQ
646
SQ
720
SQ
LSS
SQHop_cyclase_N
SQHop_cyclase_C
1
84
354
384
720
733

◀ **Figure EV3. LSS and cholesterol are downstream of ATR.**

(**A–C**) RPMI-7951 cells were transduced with lentivirus expressing shRNA targeting GFP as a control or p16 (shp16) with or without lentivirus expressing shRNA targeting ATR (shATR #1 and #2). (**A**) LSS expression was assessed by immunofluorescence staining and quantified. The graph represents individual normalized values and the mean. ****$p < 0.0001$. (**B**, **C**) Cholesterol abundance was assessed by filipin staining (**B**) and quantified (**C**). The graph represents individual normalized values and the mean. Scale bar = 20 μm. ***$p = 0.0007$, ****$p < 0.0001$. (**D**, **E**) SKMEL28 control cells were treated with hydroxyurea (HU, 500 μM for 24 h) in the presence or absence of the ATRi (AZD6738, 63 nM for 24 h). Cholesterol abundance was assessed by filipin staining (**D**) and quantified (**E**). The graph represents individual normalized values and the mean. Scale bar = 20 μm. ****$p < 0.0001$. (**F**) Fold change of *LSS* mRNA expression in the indicated groups. The graph represents mean ± SD. ns not significant, **$p = 0.007$. (**G**) Schematic of LSS protein with potential ATR phosphorylation sites. Data information: Data in (**A**–**E**) are representative data from one of three independent experiments. Graphs in (**A**, **C**, **D**) represent individual values and the mean. Data in (**F**) represent one experiment with three technical replicates. Statistical analysis in (**A**, **C**, **E**, **F**) was performed using one-way ANOVA with Šídák's multiple comparisons test.

 © The Author(s)

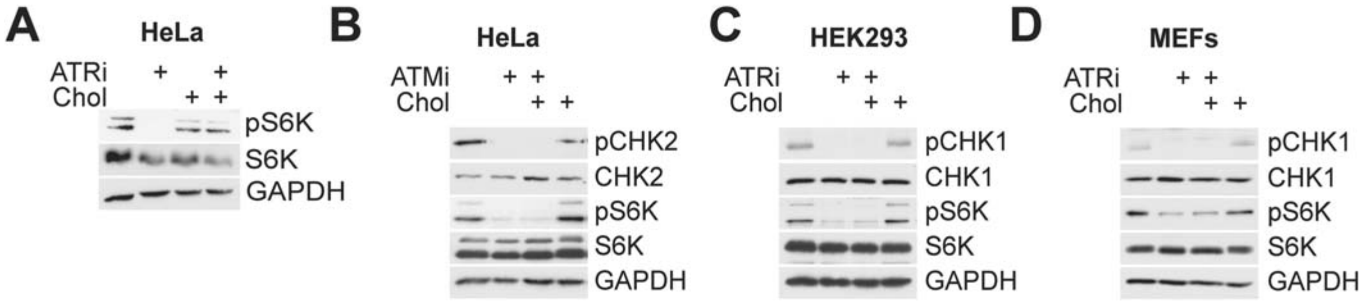


**Figure EV4. LSS and cholesterol are downstream of ATR.**

(**A**, **B**) shp16 HeLa cells were treated with ATRi (**A**) or ATMi (**B**) for 30 min in the presence or absence of supplementation with 50 μM cholesterol. (**C**, **D**) HEK293 cells (**C**) or MEFs (**D**) were treated vehicle or 0.5 μM AZD6738 (ATRi) for 30 min in the presence or absence of supplementation with 50 μM cholesterol. Data information: Western blot data in (**A–D**) are representative data from at least three independent experiments.

© The Author(s)

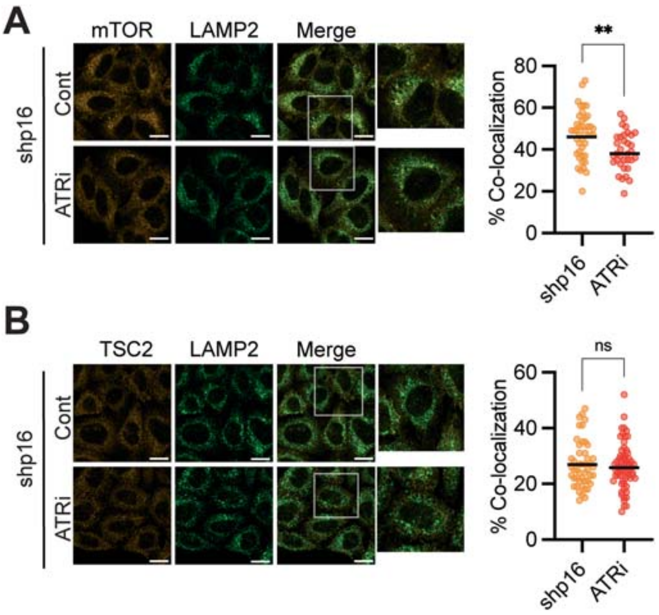


**Figure EV5. ATR inhibition decreases mTOR at the lysosome but has no effect on TSC2.**

(**A**, **B**) shp16 HeLa cells were treated for 30 min with 0.5 μM AZD6738 ATR inhibitor (ATRi) or vehicle. (**A**) Representative images of mTOR and LAMP2 immunofluorescence staining (left), which is quantified on the right. Scale bar = 20 μm. $^{**}p = 0.0015$. (**B**) Representative images of TSC2 and LAMP2 immunofluorescence staining (left), which is quantified on the right. Scale bar = 20 μm. Scale bar = 20 μm. ns not significant. Data information: Representative data from one of three independent experiments is shown. Graphs represent individual normalized values and the mean. Statistical analysis in (**A**, **B**) was performed using an unpaired two-sided Student's *t*-test. $^{**}p = 0.0015$, ns not significant.

 © The Author(s)
